# Discovery of Macrocyclic Myeloid Cell Leukemia 1 (Mcl-1) Inhibitors that Demonstrate Potent Cellular Efficacy and In Vivo Activity in a Mouse Solid Tumor Xenograft Model

**DOI:** 10.1021/acs.jmedchem.5c01376

**Published:** 2025-08-27

**Authors:** James C. Tarr, Kyuok Jeon, Nagarathanam Veerasamy, Martin Aichinger, James M. Salovich, Bin Zhao, John L. Sensintaffar, Heribert Arnhof, Tobias Wunberg, Danielle Sgubin, Allison Arnold, Rakesh H. Vekariya, Plamen P. Christov, Kwangho Kim, Julian Emanuel Fuchs, Pol Karier, Bodo Betzemeier, Mayme Van Meveren, Nagaraju Miriyala, Edward T. Olejniczak, Harald Engelhardt, Taekyu Lee, Darryl McConnell, Stephen W. Fesik

**Affiliations:** † Department of Biochemistry, 12327 Vanderbilt University School of Medicine, 2215 Garland Avenue, 607 Light Hall, Nashville, Tennessee 37232-0146, United States; ‡ Discovery Research, Boehringer Ingelheim Regional Center Vienna GmbH & Co KG, Vienna 1120, Austria; § Molecular Design and Synthesis Center, Vanderbilt Institute of Chemical Biology, Vanderbilt University, Nashville, Tennessee 37323-0146, United States; ∥ Chemical Development Germany, Boehringer Ingelheim Pharma GmbH & Co. KG, Birkendorfer Straße 65, Biberach an der Riß 88397, Germany

## Abstract

The B cell lymphoma 2 (Bcl-2) family of proteins are key regulators of intrinsic apoptosis. The antiapoptotic protein myeloid cell leukemia 1 (Mcl-1), which is associated with high tumor grade, poor survival, and resistance to treatment, has emerged as a promising candidate for treating hematological and solid cancers. Herein, we report the structure-guided design of small molecule macrocyclic Mcl-1 inhibitors based on the (*R*)-methyl-dihydropyrazinoindolone scaffold our group has previously disclosed. The macrocyclic inhibitors bind Mcl-1 with subnanomolar affinity and offer improved potency in cell culture growth inhibition assays. Inhibitor **13** achieved tumor regression in a lung cancer-derived tumor xenograft model in mice as a monotherapy. The improved potency of the macrocyclic series allowed replacement of heretofore conserved indole carboxylic acid moiety, resulting in neutral inhibitors. Amide inhibitor **25** displayed a >10-fold increase in oral bioavailability as compared to acid-containing macrocyclic or acyclic inhibitors.

## Graphical abstract

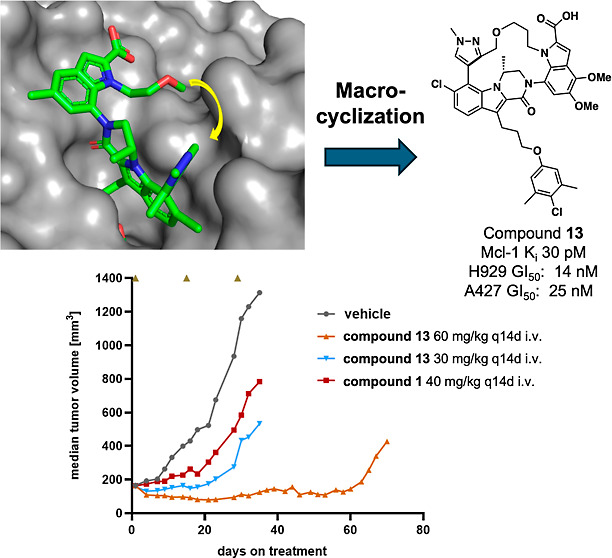

## Introduction

Apoptosis, or programmed cell death, is a highly regulated process that plays a crucial role in maintaining homeostasis and eliminating aged, excessive, or damaged cells.^1,2^ Evasion of apoptosis is a hallmark of cancer and frequently caused by dysregulation of the Bcl-2 family proteins, which regulate the intrinsic apoptosis pathway.^3,4^ The Bcl-2 family is comprised of antiapoptotic (Bcl-2, Mcl-1, Bcl-xL) and pro-apoptotic members.^5–10^ The pro-apoptotic members include the Bcl-2 homology domain 3 (BH3) only proteins (BIM, BID, PUMA, NOXA) and the multidomain effector proteins (BAK, BAX). In normal cells, antiapoptotic Bcl-2 family members bind to and sequester the pro-apoptotic members to prevent apoptotic cell death. In response to a cellular stress signal, such as DNA damage or growth factor deprivation, upregulation of the pro-apoptotic family members neutralizes the pro-survival members to liberate BAK and BAX. These effector proteins may then oligomerize and induce mitochondrial outer membrane permeabilization (MOMP), releasing cytochrome *c* from the mitochondria, and committing the cell to the caspase cascade and apoptosis. Thus, aberrant expression of pro-survival Bcl-2 family members can render the cell insensitive to death signals and enable cancer cells to evade apoptosis.^1,5,8,9^

The therapeutic utility of inhibiting pro-survival members of the Bcl-2 family proteins was first demonstrated with Venetoclax, a Bcl-2-selective inhibitor approved by the FDA for treatment of chronic lymphocytic leukemia (CLL).^11,12^ Tissues differentially express antiapoptotic Bcl-2 family members, thus different tumor types have varying degrees of sensitivity to inhibition by a given Bcl-2 family protein.^13,14^ Recently, Mcl-1 inhibition has emerged as another promising target for inducing apoptosis in cancer cells.^15–18^ MCL1 is one of the most commonly and highly overexpressed genes in a variety of cancers,^19–22^ including both hematological malignancies (leukemia, lymphoma, myeloma)^23–29^ and solid tumors (lung, breast, pancreatic, cervical, hepatic, and ovarian).^30–35^ Overexpression of Mcl-1 in mice is associated with increased risk of MYC-driven B-cell lymphomas, acute myeloid leukemia (AML), and breast cancer.^36–39^ Heterozygous loss of Mcl-1 results in inhibition of lymphomagenesis in >80% of ±mice.^40^ Furthermore, Mcl-1 upregulation is responsible for resistance development to several existing drugs including vincristine, taxol, gemcitabine, and cisplatin.^41–44^

Due to the need to closely regulate apoptosis, the antiapoptotic Bcl-2 family members bind exceptionally tightly to their respective BH3-only binding partners.^45^ The binding interaction is driven by the four hydrophobic residues (L210, L213, V216, and V220 in Mcl-1) in the conserved BH3 domain which bind to four corresponding hydrophobic pockets (P1-4) on the antiapoptotic members.^10,45–49^ To achieve potent cellular efficacy, Mcl-1 inhibitors require exceptionally low binding affinities (subnanomolar). Despite this challenge, several reported Mcl-1 inhibitors have entered clinical trials targeting both hematological cancers (AML, multiple myeloma (MM), diffuse large B cell lymphoma (DLBCL)) and solid tumors (breast, nonsmall cell lung cancer (NSCLC), small cell lung cancer (SCLC), cervical, esophageal) in both single agent and combination therapies.^50–58^ Our group has pursued an Mcl-1 inhibitor program,^59–65^ and recently reported a series of tricyclic (*R*)-methyl-dihydropyrazinoindolone inhibitors bearing an indole 2-carboxylic acid, exemplified by compound **1** (Figure 1).^66^ Compound **1** exhibited picomolar binding affinity to Mcl-1, excellent clearance in both mouse and dog, and demonstrated tumor regressions in mouse xenograft models as a single agent in multiple myeloma-derived tumors (NCI-H929 cell line) and in combination with docetaxel in Mcl-1 sensitive NSCLC A427 cell line-derived tumors. However, compound **1** proved unsuitable for clinical development. In addition to challenges finding a suitable dosing formulation for safety studies and injection site hemolysis, **1** was unable to achieve tumor regressions as a single agent in solid tumor models. Given that solid tumors (breast, lung) are generally less sensitive to Mcl-1 inhibition than hematological cancers,^13^ improved cellular potency would likely be necessary to achieve broader clinical relevance for our series of Mcl-1 inhibitors. Thus, we sought to improve the cellular and in vivo potency of **1** to identify (*R*)-methyl-dihydropyrazinoindolone inhibitors capable of achieving regressions in solid tumors as a monotherapy.

**Figure 1:**
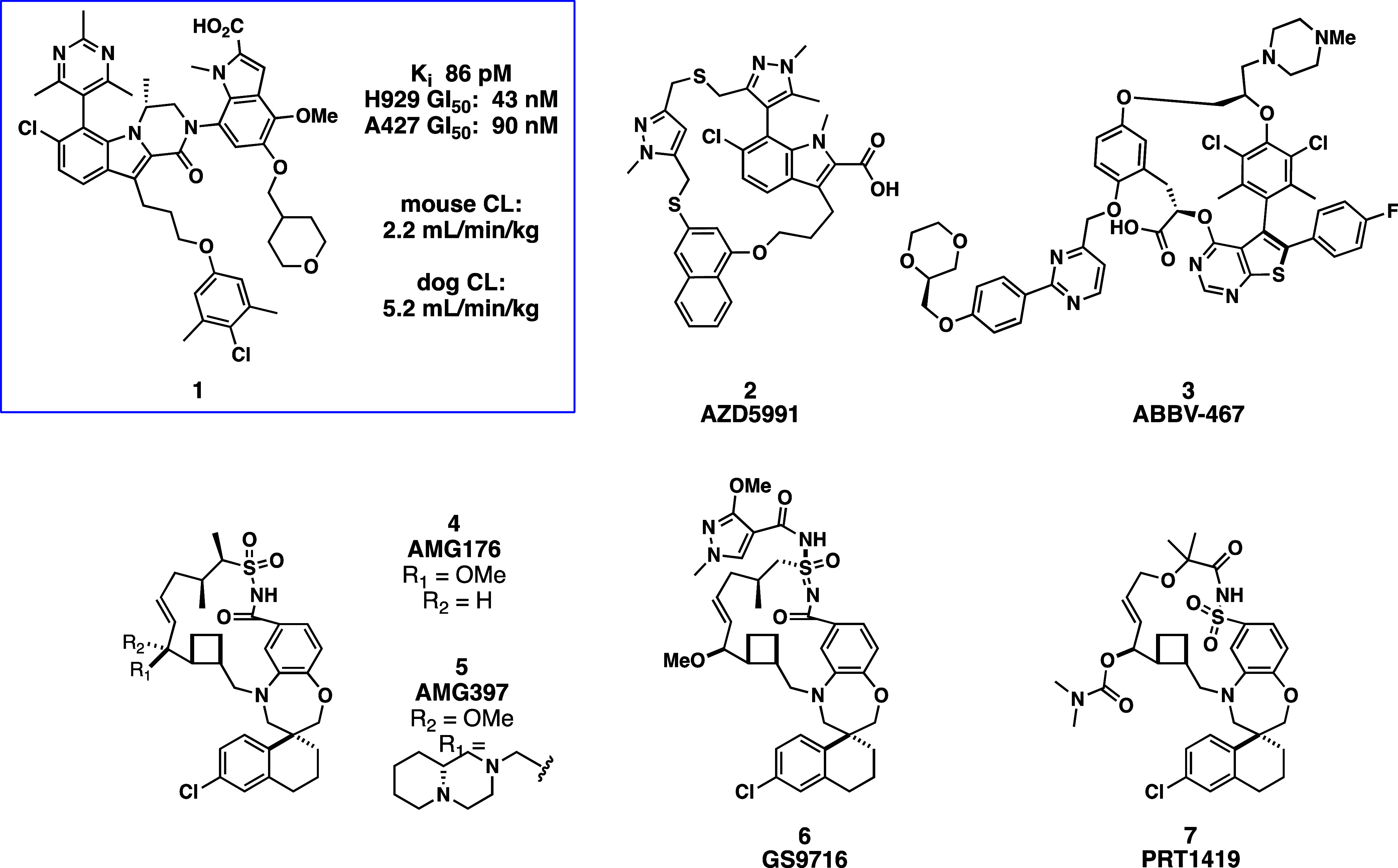
VU series inhibitor and macrocyclic clinical compounds.

One strategy that has emerged in many of the Mcl-1 clinical candidates to achieve tight binding and still maintain a favorable ADME disposition is the introduction of a macrocyclic constraint (Figure 1, compounds **2**–**7**). To overcome the tight binding affinity between Mcl-1 and pro-apoptotic Bcl-2 family members, all reported Mcl-1 inhibitors make several interactions with the protein, resulting in high molecular weight compounds (MW range from 612 to 1036 for compounds in Figure 1). Examples of macrocycles in the literature have shown improved solubility, permeability, and oral bioavailability compared to acyclic comparator compounds; characteristics that are especially important for high MW beyond rule of 5 compounds.^67–71^ In addition to potentially improving the physiochemical properties of the compounds, constraining the conformational flexibility via introduction of a macrocyclic tether may improve the binding affinity of the ligand by reducing the entropic cost of adopting the requisite binding pose. Thus, we sought to introduce a macrocyclic constraint in the (*R*)-methyl-dihydropyrazinoindolone series of inhibitors.

## Results and Discussion

### Design of Macrocyclic Mcl-1 Inhibitors

We turned to the X-ray cocrystal structure of our inhibitors bound to Mcl-1 to guide our efforts to introduce a macrocyclic tether in the (*R*)-methyl-dihydropyrazinoindolone inhibitor series. An X-ray cocrystal structure of acyclic inhibitor **8** and Mcl-1 was obtained (Figure 2) and is illustrative of the potential benefits of macrocyclization in this series. Two copies of compound **8** bound to Mcl-1 were present in the asymmetric unit, with unambiguous electron density maps of the ligand molecules suggesting two different binding poses of compound **8** (Figure 2B,C). The superimposed structures of these two ligand binding poses (Figure 2D) show a different orientation of the indole 2-carboxylic acid moiety while the other parts of compound **8** remains the same. Figure 2B,B1 show the favored binding pose, which represents the conformation adopted by the indole 2-carboxylic acid moiety of the ligand in the majority of the cocrystal structures we have obtained in the program. This binding pose provides optimal overlap between the Arg263 residue and the indole ring resulting a cation–π interaction. Additionally, the 2-carboxylic acid moiety forms a polar interaction with the Asn260 side chain (Figure 2B1). However, as we occasionally observed in our X-ray cocrystals, the ligand may also adopt a binding pose where the indole 2-carboxylic acid is rotated 180° around the C–N amide bond (Figure 2C,C1). This binding pose is accompanied by a modest shift in the left-hand portion of the Mcl-1 loop region to accommodate the 2-carboxylic acid moiety, which now forms a hydrogen bonding interaction with the Val258 backbone NH. This pose also results in a less ideal overlap between the indole ring and Arg263 for the cation–π interaction. We hypothesized that if we could fix the indole 2-carboxylic acid moiety into the more favored binding pose (Figure 2B2), we would increase the binding affinity and thereby cellular potency of our inhibitors. Inspection of the cocrystal structure of **8** and Mcl-1 shows there is an accessible shelf region that is already partially occupied by the indole 2-carboxylic acid *N*-substituent (*N*-ethyl methyl ether in the case of **8**) that the tether could occupy (Figure 2B2). We envisioned extending the indole nitrogen substituent and linking it to the pyrazole C3 or C5 position. Due to synthetic tractability, we planned to use a substituted pyrazole, rather than pyrimidine, as the aryl substituent at the indole C7-position. We anticipated that cyclization should occur from the lactam face opposite the (*R*)-methyl group, precluding formation of the undesired macrocyle (Figure 2C2). Substitution of the indole nitrogen position with a variety of substituents (alkyl, heteroalkyl, aryl, heteroaryl) had been well tolerated in previous SAR, giving us confidence that a suitable linker could be accommodated. Analysis of the crystal structure of **8** indicated that a 5-atom linker between the nitrogen of the indole 2-carboxylic acid moiety and the pyrazole ring should be optimal for maintaining a similar binding pose with the proposed macrocyclic ligand as observed in the acyclic inhibitor series. Glide docking studies further validated this design, indicating that the proposed macrocyclic ligand could maintain optimal interactions within the binding site (see Supporting Information S2).

**Figure 2:**
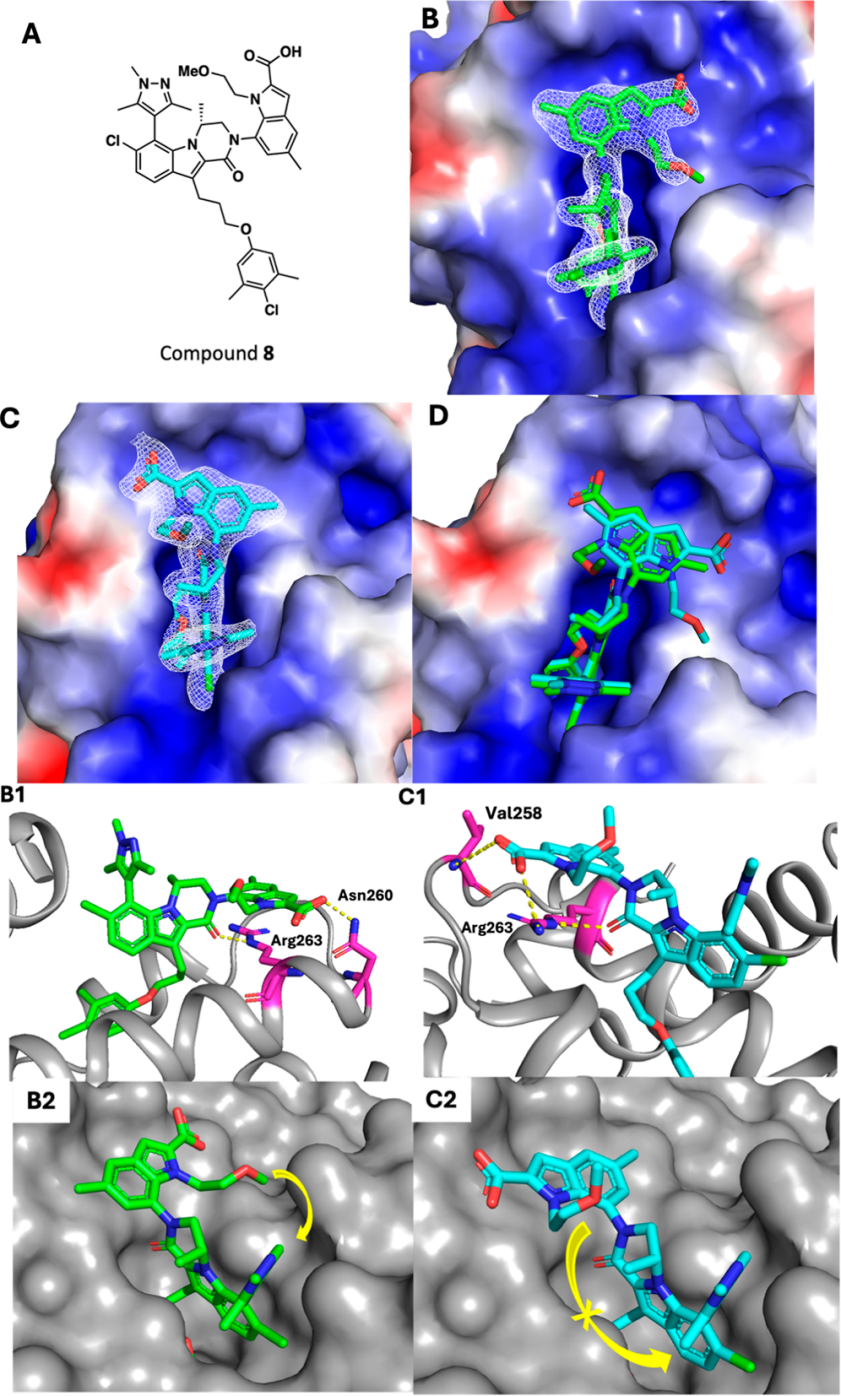
X-ray co-crystal structure of compound **8** and Mcl-1 (9PW6). (A) Structure of compound **8**. (B) First binding pose of **8** (green) cocrystallized with Mcl-1 and its 2F_o_-F_c_ electron density map. (C) Second binding pose observed in **8** (cyan) cocrystallized with Mcl-1 and its 2F_o_-F_c_ electron density map. (D) Overlaid cocrystallized Mcl-1 structures of two binding poses of compound **8**. B1. Key interactions between indole carboxylic acid of **8** (green) and Mcl-1 residues Asn260 and Arg263 in first binding pose. C1. Interactions between indole carboxylic acid of **8** (cyan) and Mcl-1 residues Val258 and Arg263 in second binding pose. B2. Tethering opportunity between indole nitrogen substituent and pyrazole 3-position substituent. C2. Undesired macrocyclization tethering possibility, disfavored by steric clash with lactam methyl group.

### Initial Macrocyclic Inhibitors

To test our hypothesis, macrocycle **10** was synthesized incorporating a 5-atom ether tether between the indole nitrogen and pyrazole C3 position (Figure 3). The compound was evaluated and compared to the analogous acyclic inhibitor **9** in a time-resolved fluorescence energy transfer (TR-FRET) binding assay and cell growth inhibition in H929 and A427 cell lines, which have both been shown to be sensitive to Mcl-1 inhibition.^64,72,73^ Comparison of our initial macrocycle **10** to the analogous acyclic compound **9** shows a 2-fold improvement in both H929 and A427 cellular potency. In addition to showing improved potency, compound **10** also showed a 10-fold reduction in mouse i.v. clearance (CL) relative to **9**. The impact of macrocyclization on molecular properties between **9** and **10** was also compared. Tethering results in a small increase in molecular weight (MW = 772 (**9**), 814 (**10**)), but reduces the number of rotatable bonds from 10 to 8. The macrocycle **10** shows an increase of 10 Å^2^ in total polar surface area (TPSA), but a half-log decrease in the calculated distribution coefficient (clog *D* = 7.968 (**9**) vs 7.586 (**10**)). Synthesis of the regioisomeric compound **11** showed that tethering from the pyrazole C3- or C5-position had no impact on potency. Finally, variation of the linker length confirmed that the 5-atom tether was optimal, as predicted by modeling and analysis of the cocrystal structure (compound **8**, Figure 2). Homologation to the 6-atom tether (**12**) resulted in a 2-fold decrease in potency. While encouraged by these initial results, further exploration of this series was hampered by low overall synthetic yields (e.g., <2% for **10**). To address these limitations, a second-generation synthetic route was developed to access the pyrazole C5-des-methyl analog (**13**). In addition to the improved synthetic tractability, compound **13** also showed a modest improvement in potency over the C5 methyl analog **10**. A minimal difference in mouse i.v. CL was observed between **10** and **13**; however, the des-methyl series offered significantly better aqueous solubility compared to the C5 methyl series (**10**: 2 mg/mL kinetic solubility at pH 6.8, **13**: 85 mg/mL kinetic solubility at pH 6.8). The oral bioavailability (F) of compound **13** was assessed, and showed no improvement over the acyclic series (*F* = 1%, AUC_IV_ = 60,900 nM*h at 20 mg/kg; AUC_PO_ = 625 nM*h at 20 mg/kg). Phospholipid and cyclodextrin additions to the formulations did not increase the oral bioavailability of **13**.

**Figure 3:**
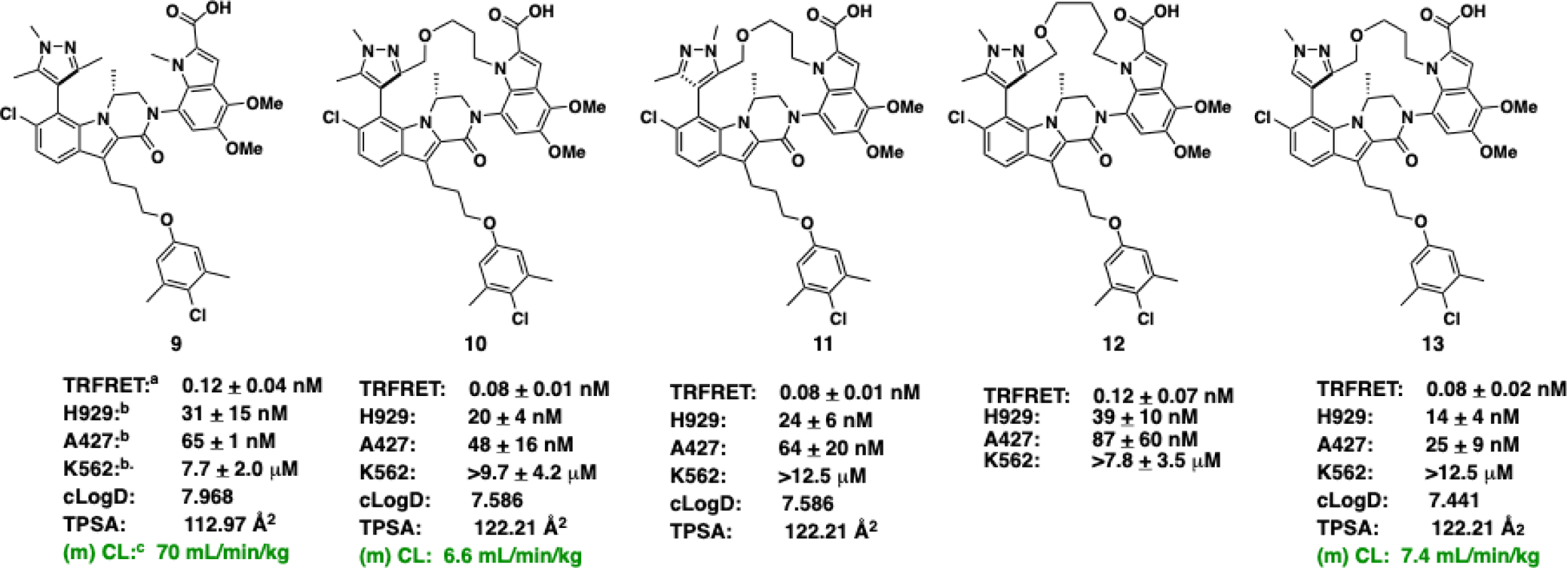
SAR of initial macrocyclic Mcl-1 inhibitors. ^a^TR-FRET binding affinity to Mcl-1, *K*_i_ measured in the presence of 1% fetal bovine serum. All data points are an average of at least *n* = 2, run in triplicate. ^b^Cellular growth inhibition data points are an average of at least *n* = 2, run in triplicate. ^c^i.v. mouse CL measured at 20 mg/kg.

To demonstrate that the observed antiproliferative activity was due to on-target inhibition of Mcl-1 rather than off-target interactions, the compounds were counterscreened using the Mcl-1 insensitive K562 cell line (Figure 3). In each of the examples, the compounds maintained excellent selectivity for Mcl-1-sensitve cell lines over the K562 control (>900-fold for compound **13**), indicating that the compounds continue to exert their effects via on-target Mcl-1 inhibition as previously established for the acyclic (*R*)-methyl-dihydropyrazinoindolone inhibitor series.^63,66^ To further corroborate that compound **1** induced cell death via the apoptotic pathway, its EC_50_ for caspase 3/7 induction in H929, A427, and K562 was measured (Figure 4). The EC_50_’s of caspase 3/7 induction for both heme (H929) and solid tumor (A427) Mcl-1 sensitive cell lines tracked closely with the observed GI_50_’s, and showed a 13- to 16-fold increase over the vehicle. The Mcl-1 insensitive K562 cell line showed no caspase induction. Compound **13** also maintained high selectivity (>50,000-fold) for Mcl-1 over other antiapoptotic Bcl-2 family members Bcl-2 and Bcl-xL.

**Figure 4:**
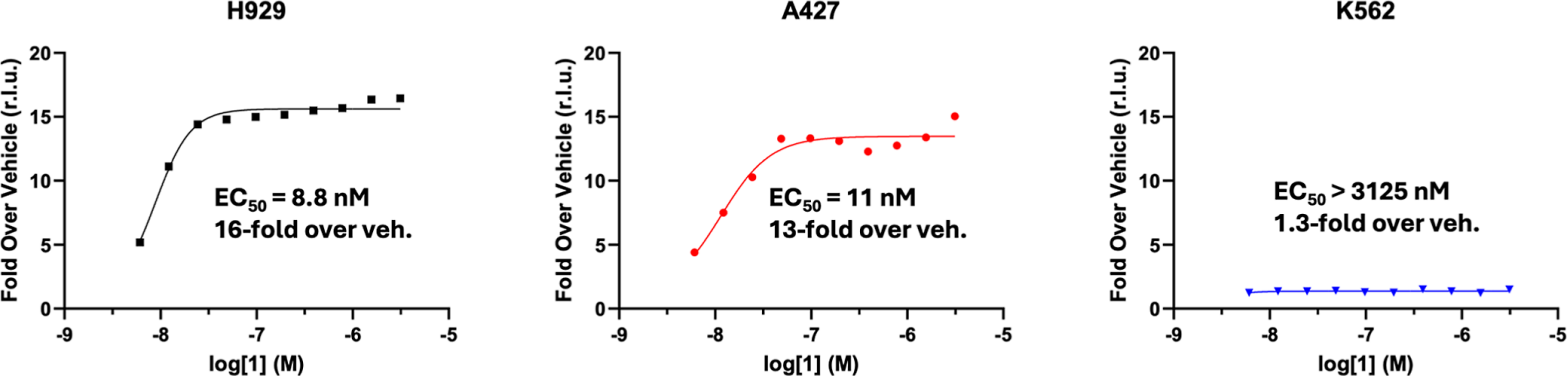
Caspase 3/7 induction in H929, A427, and K562 cell lines.

### Analysis of X-ray Co-Crystal Structure of Mcl-1 Bound Macrocyclic Inhibitor

To understand the binding interactions of the macrocyclic inhibitors, the X-ray cocrystal structure of **13** bound to Mcl-1 was obtained (Figure 5). Additionally, the X-ray cocrystal structure of the acyclic direct comparator **9** was also collected.^66^ The X-ray crystal structure of **13** (Figure 5B) confirms our expectations that the single macrocyclic diastereomer produced cyclizes from the top face, avoiding the steric clash with the lactam (*R*)-methyl group on the opposite face. Compound **13** maintains a very similar binding pose as acyclic inhibitor **9** (Figure 5C), preserving the same key interactions that drive the exceptionally tight binding to Mcl-1 in the acyclic series (face-to-edge interaction between the 4-chloro-3,5-dimethylphenyl group and Phe270 (Figure 5B3), cation–π stacking between the dimethoxy indole moiety and Arg263, and hydrogen bonding of indole carboxylic acid with Asn260 (Figure 5B1). The introduction of the 5-atom tether results in an ∼8° shift in the dihedral angle between the core indole and the pyrazole ring, with the tethered pyrazole substituent of **13** angled toward nitrogen of the indole carboxylic acid (Figure 5C1). There is also a slight repositioning of the Mcl-1 loop portion, with the loop region positioned further away from the indole 5-position substituent in the cocrystal structure of **13** as compared to **9**. In the cocrystal structure of **13**, as well as each other macrocyclic cocrystal structure we have obtained, the oxygen atom of the tether is positioned away from the surface of Mcl-1, presumably to avoid a repulsive interaction with Thr266 (Figure 5B2).

**Figure 5:**
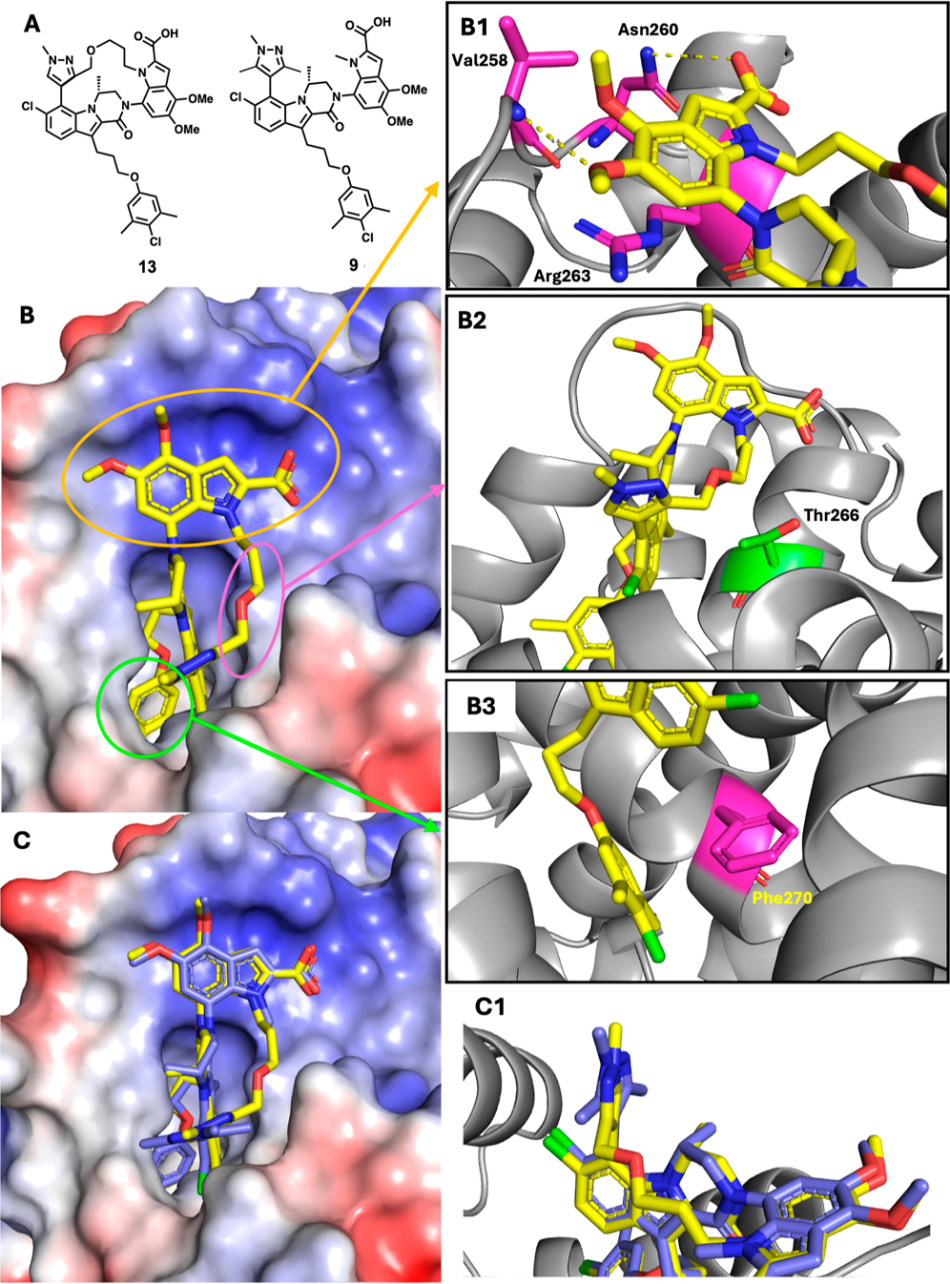
X-ray cocrystal structures of compounds **13** and **9** bound to Mcl-1. (A) Structure of compound **13** and **9**. (B) X-ray cocrystal structure of compound **13** (yellow) bound to Mcl-1 (9PW7). B1. Orange inset. Interactions between indole moiety and Val258, Asn260, and Arg263. B2. Pink inset. Position of the ether in 5-atom tether relative to Thr266. B3. Green inset. π–π interaction between 3,5-dimethyl-4-chlorophenol moiety and Phe270. (C) Overlay of compounds **13** (yellow) and **9** (blue) bound to Mcl-1. C1. Comparison of dihedral angle between the tricyclic core and the C7 pyrazole in **13** (yellow) and **9** (blue).

### SAR of Second-Generation Macrocyclic Inhibitors

We next profiled a library of analogs based on the scaffold of compound **13** and varying the substitution pattern at the indole 4- and 5-positions. The compounds were evaluated in a TR-FRET binding assay, cell proliferation assays using H929 and A427 tumor cell lines, and mouse PK (Table 1). The SAR trend for the binding affinity and cellular potency is generally consistent with the acyclic series in that varying the indole substituents results in relatively modest changes in binding affinity and cellular potency. The TR-FRET binding assay provided little texture, as all compounds that were tested exhibited sub-100 picomolar binding affinity, approaching the lower limit of linear range for the assay (*K*_i_ below 0.080 nM). Thus, the growth inhibition in the cellular assays were a more meaningful method to differentiate analogs. As was the case in the acyclic inhibitor series, disubstitution is preferrable with regards to potency, with monosubstituted compounds **14**, **15**, and **16** showing a 2–5-fold decrease in cellular potency. Electron donating indole substitutions continue to result in higher cellular potency, as **14**, which bears a single methyl substituent on the indole, exhibits in the weakest cellular potency in both the H929 and A427 cell lines. Replacement of one of the methoxy substituents of **13** with a methyl group resulted in a ∼1.5–2.5-fold loss of potency (**17** and **18**), with retention of the 5-position methyl ether being better tolerated. Finally, varying the 5-position ether substituent also exerts a modest impact on potency, with the trend generally tracking the acyclic series. For example, the methoxy ethyl ether in **19** results in improved potency, whereas the methyl tetrahydropyran in **20** results in a ∼2-fold decrease in potency. Although subtle in the indole-2-carboxylic acid examples, an emerging trend noted was that suboptimal indole substitution patterns on the macrocyclic scaffold (e.g., **14**, **15**, **17**, and **20**) resulted in smaller decreases in potency than that observed for the same substitution patterns on the acyclic series.^66^ To monitor off-target effects, the GI_50_ in K562 was measured, and caspase 3/7 induction in H929, A427, and K562 was measured. In all instances, the K562 GI_50_ is 2–3 orders of magnitude higher than the sensitive cell lines and the caspase induction EC_50_ closely tracks the observed GI_50_. The H929 caspase induction EC_50_ and fold over vehicle are reported in Table 1, and the K562 GI_50_’s and caspase induction in A427 and K562 are available in the Supporting Information.

**Table 1:**
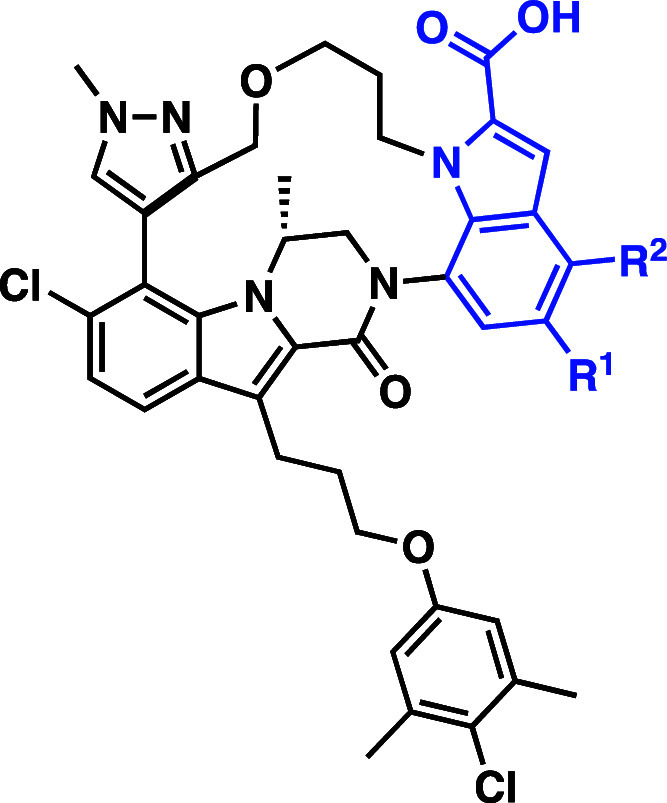

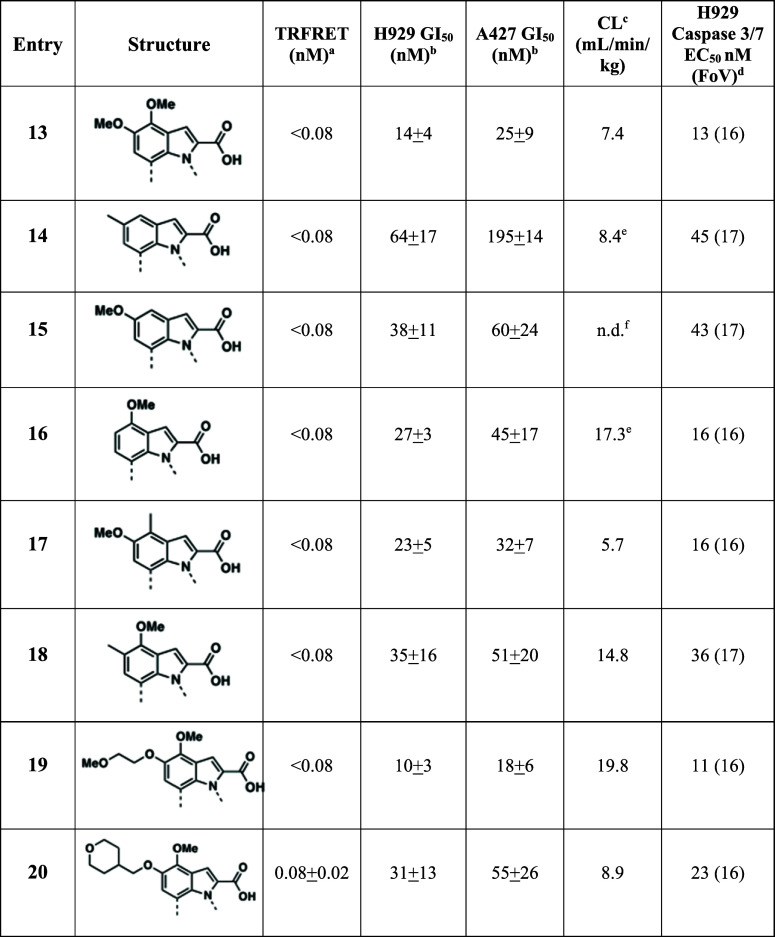
Mcl-1 Inhibitors Evaluated in Cell Lines and Mouse PK

a Mcl-1 *K*_i_ in the presence of 1% fetal bovine serum. All data points are an average of at least *n* = 2, run in triplicate.

b All data points are an average of at least *n* = 2, run in triplicate.

c Dosed at 20 mg/kg unless otherwise indicated.

d EC_50_ of caspase 3/7 activation after compound treatment for 3 h. Maximum activity reported as fold increase over vehicle treated cells (FoV).

e Dosed at 10 mg/kg.

f Compound not evaluated.

While the potency SAR closely tracks with the acyclic series, notable deviations were observed in the mouse PK. The des-methyl macrocycle **13** possessed an i.v. clearance in mice of 7.4 mL/min/kg, similar to **10** (6.6 mL/min/kg). While the acyclic 4,5-dimethoxy indole **9** showed significantly higher CL than other indole substitutions, the macrocycles showed less variation. All compounds examined displayed a CL within a factor of ∼2 of compound **13**. The only compound that resulted in an improved CL was **17**, replacing the indole 4-OMe group with a methyl substituent. However, the improvement to CL was quite modest, and aqueous solubility of **17** was half that of **13** (**17**: 39 μg/mL kinetic solubility at pH 6.8). While introduction of an methoxyethyl ether substituent at the 5-position in the acyclic series^66^ results in a minimal change to CL, in the macrocyclic series this substituent (**19**) results a 2-fold increase in CL. Most importantly, in the acyclic series the 4-methyl tetrahydrofuran ether resulted in a 20-fold reduction in mouse CL;^66^ however, the same ether substituent (**20**) on the macrocyclic scaffold resulted in no improvement from the 4,5-dimethoxy analog **13**. Given that none of the substitution patterns evaluated presented a clear advantage to the compound **13**, the 4,5-dimethoxy substituents were used to benchmark the macrocyclic series in vivo and a starting point for future analogs.

In addition to exploring indole substitution patterns, we examined additional modifications to the macrocyclic scaffold (Table 2). Replacement of the pyrazole moiety with pyridine was evaluated with analogs **21** and **22**. Compound **21** maintained similar binding affinity, cellular potency and CL as **13**; however, compound **22** led to improved mouse CL of 3.5 mL/min/kg. Unfortunately, compound **22** suffered from low aqueous solubility (11 μg/mL kinetic solubility at pH 6.8), which precluded its progression. We also further explored the previously noted trend that less optimal indole substitution patterns were better tolerated in terms of cellular potency in the macrocyclic series. We prepared the 6-des chloro analog **23**, which showed nearly equal potency to **13**. By comparison, removal of the 6-Cl group in the acyclic series resulted in a 5-fold reduction of potency. Encouraged by these results, we looked to modify the indole 2-carboxylic acid moiety, preservation of which had previously been required for maintaining tight binding to Mcl-1 and robust cellular efficacy. Despite its contribution to the binding affinity to Mcl-1, the carboxylic acid moiety plays a key role in the clearance and oral bioavailability of the macrocyclic series of compounds. Biliary clearance is the major route of metabolism for the acyclic inhibitor **1**, with the very low hepatic clearance resulting in excellent overall CL in mouse (2.2 mL/min/kg). While the rate of biliary clearance of **13** is comparable to that of **1**, the hepatic clearance is ∼6-fold higher, primarily driven by acyl glucuronide formation on the carboxylic acid. Thus, we hypothesized replacement of the carboxylic acid could have a profound impact on the compound’s clearance. A pair of dimethyl amides, **24** and **25**, were synthesized by HATU coupling reactions from carboxylic acids **1** and **13**, respectively. As predicted, loss of the indole 2-carboxylic acid in acyclic analog **24** resulted in a significant loss of cellular potency, 25-fold in H929 and 40-fold in A427. Gratifyingly, macrocyclic dimethyl amide **25** exhibited only a 2–3 fold loss of potency in cellular antiproliferative assays, with a GI_50_ of 39 nM in the H929 cell line and 105 nM in the A427 line. These potencies are in line with our previous lead molecule (**1**) from the acyclic inhibitor series. While introduction of the dimethyl amide had a minimal impact on the mouse clearance (CL = 6.4 mL/min/kg), it had a profound impact on the oral bioavailability. Compound **25** achieved an oral bioavailability of 22%, greater than a 10-fold improvement to the <2% bioavailability observed for both our acyclic and macrocyclic carboxylic acid-containing inhibitors.

**Table 2:**
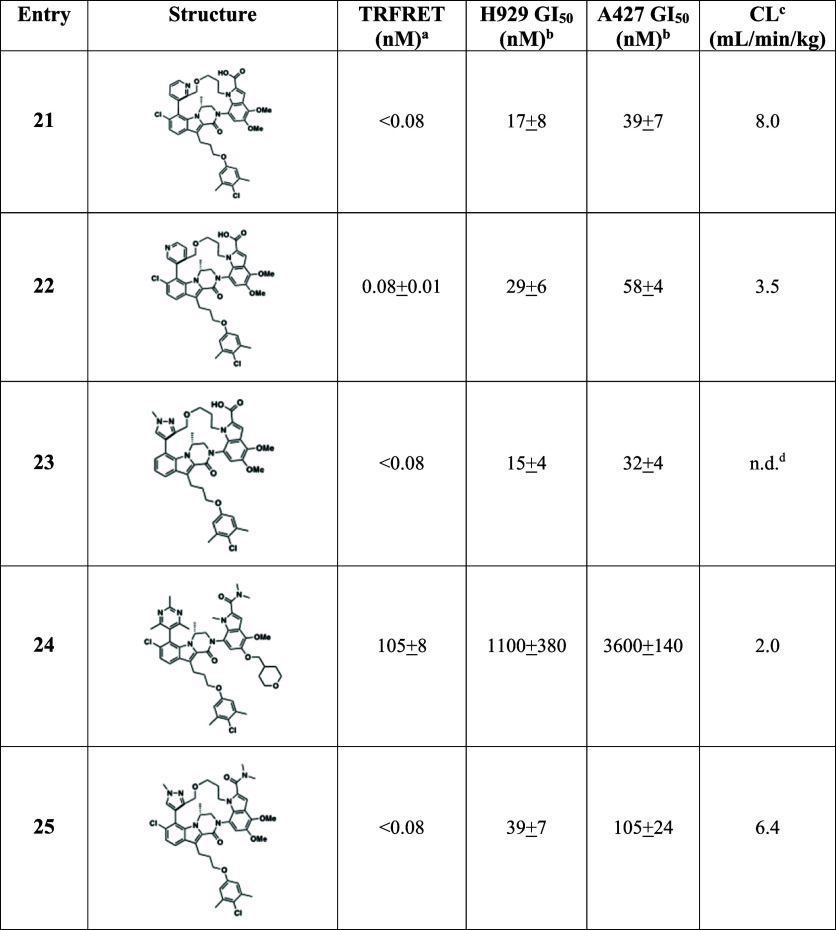
Mcl-1 Inhibitors Evaluated in Cell Lines and Mouse PK

a Mcl-1 *K*_i_ in the presence of 1% fetal bovine serum. All data points are an average of at least *n* = 2, run in triplicate.

b All data points are an average of at least *n* = 2, run in triplicate.

c Dosed at 20 mg/kg.

d Compound not evaluated.

### Activity in Solid Tumor Xenograft Model

The in vivo efficacy of the macrocyclic inhibitor series was benchmarked against the acyclic Mcl-1 inhibitors using compounds **1** and **13** in a subcutaneous NSCLC xenograft model with A427-derived tumors (Figure 6). Compound **1** was dosed at 40 mg/kg every 14 days, which was projected to be the minimum dose expected to achieve a tumor growth inhibition (TGI) of 60%. We have previously shown with compound **1** that the (*R*)-methyl-dihydropyrazinoindolone inhibitors exert their biological activity via on-target inhibition of Mcl-1 by disruption of the Mcl-1/BIM complex and caspase 3/7 induction in vivo.^66^ At study day 28, animals dosed with compound **1** showed a 57% TGI with 0/8 animals showing tumor regressions. Compound **13** was dosed at 30 and 60 mg/kg. At the 30 mg/kg dose, the exposure of **13** was ∼75% that achieved with compound **1**. However, the increased cellular potency of **13** translated to the in vivo setting, with **13** displaying a TGI of 85% despite the lower exposure. No animals experienced tumor regressions at this dose of **13**. Increasing the dose of compound **13** to 60 mg/kg resulted in a higher exposure (481,000 nM*h), and a corresponding higher TGI (109%). At this dose, 7/8 animals exhibited tumor regressions at day 28, marking a significant breakthrough for in vivo efficacy for this series. Importantly, this increased activity did not result in tolerability problems, and body weight behaved similarly to the vehicle control cohort. Our acyclic series of Mcl-1 inhibitors had been able to achieve tumor regressions in hematopoietic xenograft models as a single agent, but only growth inhibition in solid (lung and breast) xenograft models (Figure 6A and data not shown).^66^ In addition to achieving tumor regressions, the duration of response to compound **13** was substantially longer than previously observed in this model for compound **1** (compound **26** in ref 66). When **13** was dosed at 60 mg/kg, outgrowth occurred around study day 50, approximately 20 days after the last dose of the inhibitor.

**Figure 6:**
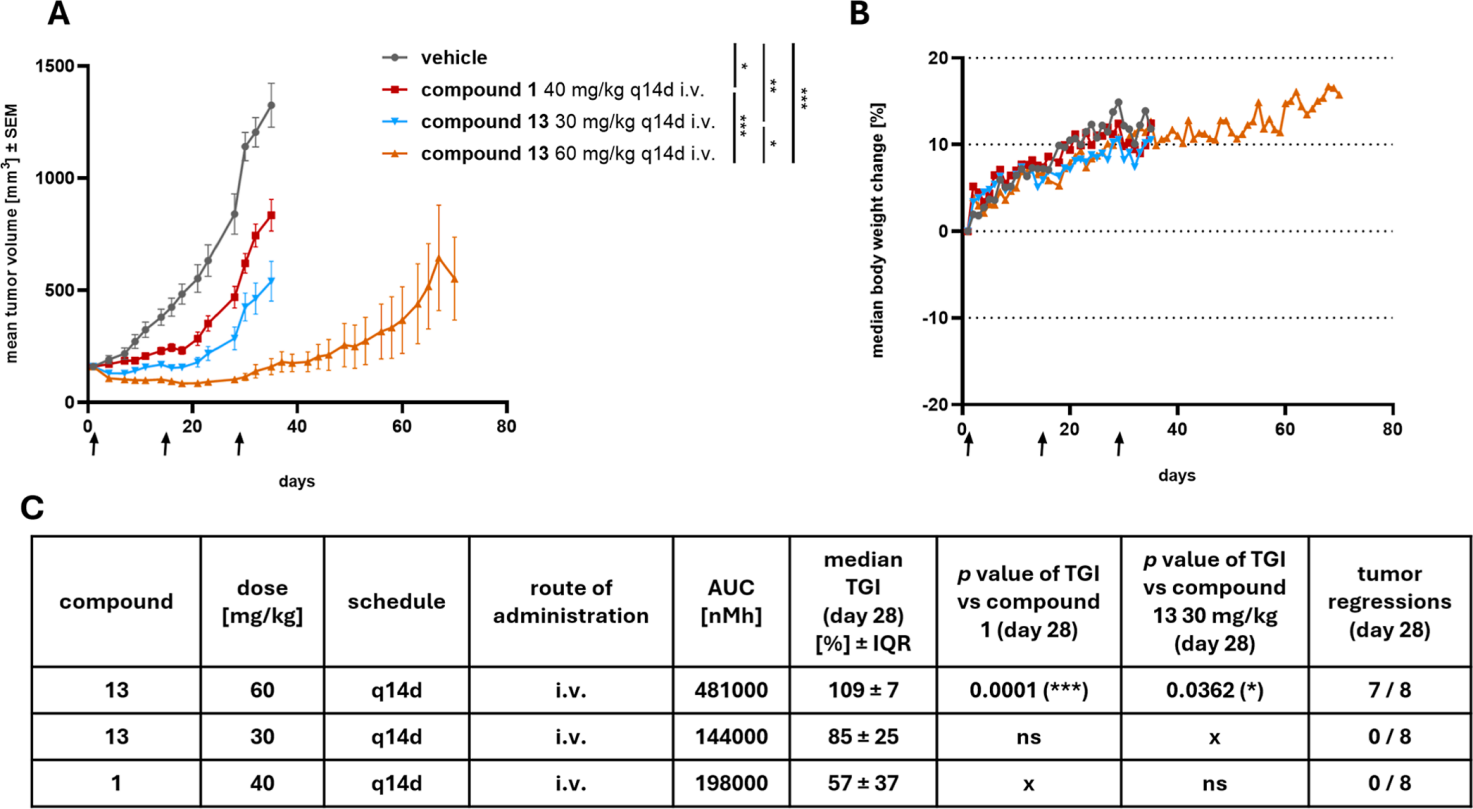
Efficacy study of **13** in A427 cell line-derived NSCLC xenograft model (*n* = 4–8 per group). (A) Mean tumor volume of control (gray), **1** dosed at 40 mg/kg (red), **13** dosed at 30 mg/kg (blue), **13** dosed at 60 mg/kg (orange) administered q14d i.v. Statistical analysis was performed on day 28 (last measurement day of *n* = 8 per group) using a mixed-effects analysis with Tukey’s multiple comparisons test (*, *P* < 0.05; **, *P* < 0.01; ***, *P* < 0.001). Arrows indicate the day of treatment. Initial average tumor volume at the treatment start was 160 mm^3^. Error bars represent SEM. (B) Body weight change compared to the baseline (initial body weight at the treatment start) of control (gray), **1** dosed at 40 mg/kg (red), **13** dosed at 30 mg/kg (blue), **13** dosed at 60 mg/kg (orange) test groups. Body weight change is shown as median percentage. Arrows indicate the day of treatment. (C) Tabulation of dose, AUC, tumor growth inhibition (TGI), and tumor regression. Statistical analysis of TGI was performed on day 28 (last measurement day of *n* = 8 per group) using a Kruskal–Wallis test with Dunn’s multiple comparisons (*, *P* < 0.05; ***, *P* < 0.001). Error bars of median TGI represent IQR (interquartile range).

### Initial Macrocyclic Inhibitor Synthesis

The synthesis of macrocyclic inhibitor **10** is detailed in Scheme 1. To introduce the macrocyclic tether, we envisioned using a methyl alcohol as a synthetic handle at the pyrazole C3-position, which could then be linked to the indole nitrogen via a 3-carbon spacer. The benzyl-protected pyrazole alcohol is introduced in the molecule via Suzuki coupling between bromide **26** and pinacol borane **27**. Alkylation of the indole nitrogen with *tert*-butyl (*S*)-5-methyl-1,2,3-oxathiazolidine-3-carboxylate 2,2-dioxide (**29**), Boc deprotection, and cyclization affords the (*R*)-methyl dihydropyrazinoindolone core **30**. Importantly, once the lactam ring is installed, the steric hindrance around the fully substituted biaryl linkage between the dihydropyrazinoindolone core and the pyrazole ring precludes rotation, even under prolonged heating. Due to the lack of stereocontrol for the facial orientation of the benzyl alcohol, a ∼1:1 mixture of diastereomeric atropisomers are formed (*M*-**30** and *P*-**30**, Scheme 1), only one of which is oriented to cyclize to the desired product (cyclization from the opposite face results in the unfavored conformation shown in Figure 2C). We reasoned that steric hindrance from the chiral (*R*)-methyl group would prevent cyclization from the undesired face. Introduction of the indole 2-carboxylic acid moiety was accomplished by Ullmann with intermediate **31** using CuI/(*trans*)-*N*,*N*′-dimethylaminocyclohexane as the catalyst. Hydrogenolysis of the benzyloxy ether **32** affords compound **33**. Closing the macrocycle was accomplished via stepwise alkylation of 1,3-dibromopropane. Alkylation of the indole NH was first carried out by heating with Cs_2_CO_3_, followed by alkylation of the pyrazole C3 methyl alcohol with NaH. As predicted, cyclization only occurs from the face opposite the (*R*)-methyl group, and no undesired macrocycle was observed. In addition to the desired cyclization step, a significant amount of elimination byproduct was also observed. During the methyl alcohol alkylation, concomitant saponification of the ethyl ester affords macrocyclic acid **10**.

**Scheme 1:**
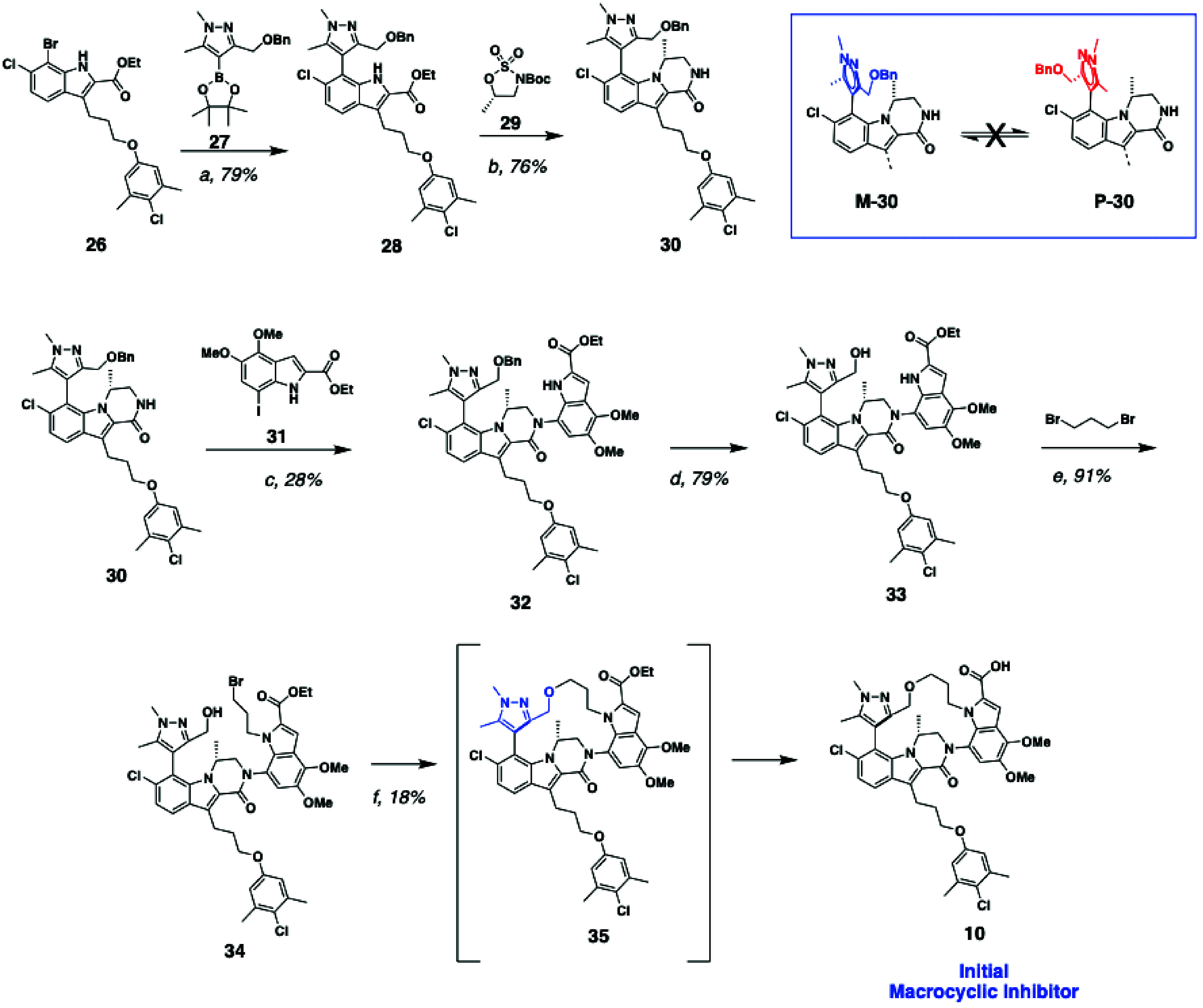
Synthesis of Initial Macrocyclic Acid^a^

a Conditions (a) Pd­(PPh_3_)_4_, K_2_CO_3_, dioxane/water, 140 °C, MW irradiation, 30 min (b) (1) **29**, Cs_2_CO_3_, MeCN, 80 °C, 24 h. (2) TFA, DCM, RT, 1 h. (3) K_2_CO_3_, MeOH, RT, 60 h. (c) CuI, (*trans*)-*N*,*N*′-dimethylaminocyclohexane, K_2_CO_3_, toluene, 100 °C, 48 h. (d) Pd/C, H_2_, MeOH, 30 °C. (e) Cs_2_CO_3_, DMF, 40 °C, 30 h. (f) NaH, DMF, RT, 96 h.

### Second-Generation Macrocyclic Inhibitor Synthesis

It was necessary to refine the synthetic route (Scheme 2, top panel) to our macrocyclic inhibitors before we could fully explore the SAR of this new series, as the yield of compound **10** from **26** was prohibitively low (2% yield) for a discovery campaign. Major contributors to the low overall yield are (a) only half of the material (*M* atropisomer) from intermediate **34** can productively cyclize, (b) elimination of the bromide of intermediate **34** rather than productive cyclization to **35**, and (c) low and variable yields in the cross coupling reaction between **30** and **31**. We first addressed the hindered rotation around the biaryl linkage in intermediates **30**–**34**. In our previously reported acyclic Mcl-1 inhibitors, bis­(ortho) substitution on the pyrazole ring was necessary for good binding affinity, as it enforced the preferred nearly orthogonal dihedral angle between the pyrazole ring and the (*R*)-dihydropyrazinoindole core. However, we postulated that introduction of the macrocyclic tether may be sufficient to maintain the desired orientation without requiring the additional methyl group at the pyrazole C5 position. Studies on model systems demonstrated that removal of the pyrazole C5 methyl group allows for rotation around the biaryl axis, allowing the C3 methyl alcohol to rotate to the desired face and achieve complete conversion of the starting material to product (Figure 7). Thus, we introduced a pyrazole moiety lacking substitution at the C5 position (Scheme 2).

**Scheme 2:**
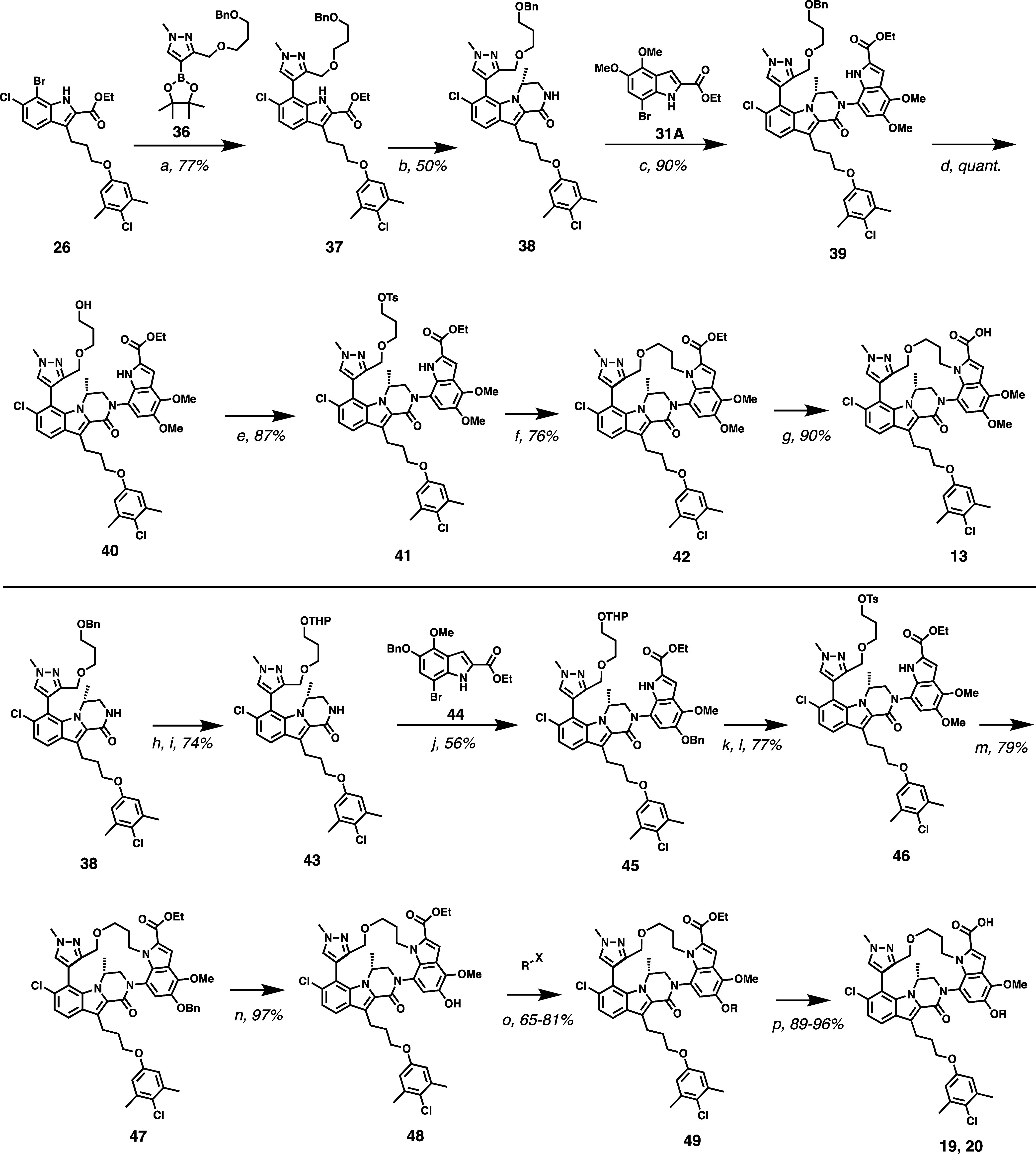
Second Generation Improved Synthesis of Macrocyclic Acids^a^

a Conditions: (a) Pd­(PPh_3_)_4_, K_2_CO_3_, dioxane/water 100 °C, 8 h. (b) (1). **29**, Cs_2_CO_3_, MeCN, 80 °C, 24 h. (2) TFA, DCM, RT, 2 h, (3) K_2_CO_3_, MeOH, 50 °C, 3 h. (c) **31A**, [Pd­(cinnamyl)­Cl]_2_, ^*t*^Bu-BrettPhos, Cs_2_CO_3_, toluene, 100 °C, 2 h. (d) Pd/C, Pd­(OH)_2_/C, H_2_, THF/*i*PrOH, 30 °C, 3 h. (e) Ts_2_O, DMAP, DIPEA, DCM, RT, 2 h. (f) Cs_2_CO_3_, DMF, 60 °C, 16 h. (g) LiOH, THF/MeOH/H_2_O, 40 °C, 16 h. (h) Pd/C, Pd­(OH)_2_/C, H_2_, THF/*i*PrOH. (i) THP, TsOH, THF. (j) **44**, [Pd­(cinnamyl)­Cl]_2_, ^*t*^Bu-BrettPhos, Cs_2_CO_3_, toluene. (k) TsOH, MeOH, H_2_O. (l) Ts_2_O, DMAP, DIPEA, DCM. (m) Cs_2_CO_3_, DMF. (n) Pd/C, Pd­(OH)_2_/C, H_2_, THF/*i*PrOH. (o) R–X, Cs_2_CO_3_, DMF. (p) LiOH, THF/MeOH/H_2_O.

**Figure 7:**
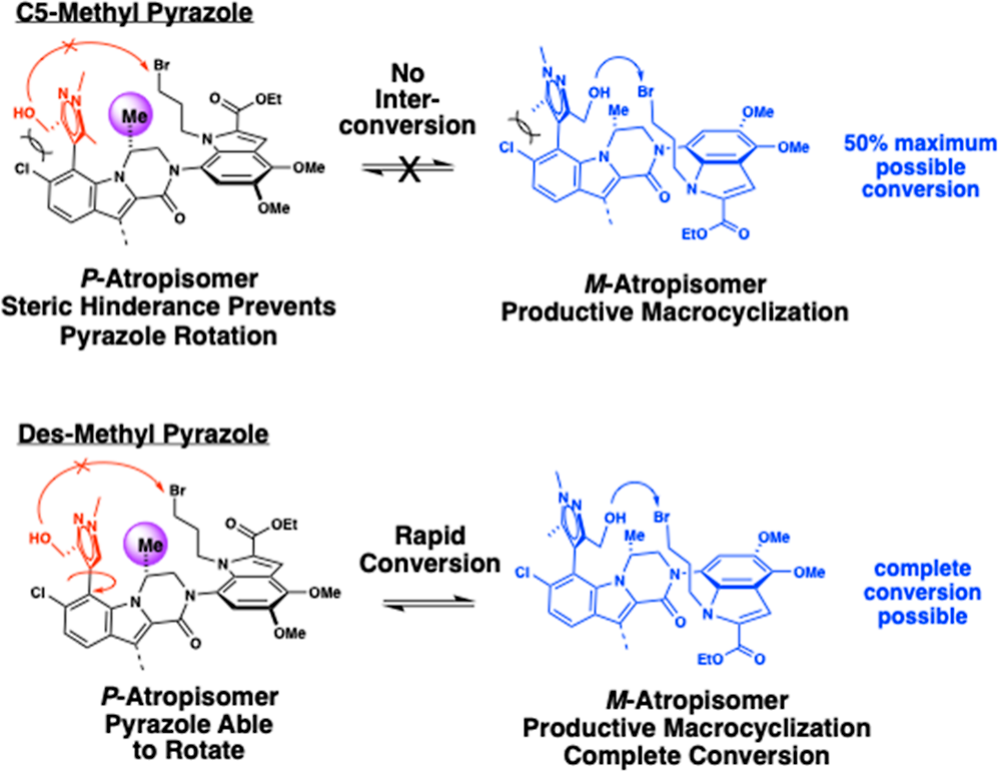
Impact of des-methyl pyrazole on cyclization.

We next sought to minimize unproductive elimination of intermediate **34** to the terminal alkene. This competing side reaction is largely due to the poor nucleophilicity of the methyl alcohol at the pyrazole 3-position. We reasoned that incorporation of the propyl portion of the macrocyclic tether on the pyrazole moiety first and then relying on the more nucleophilic indole nitrogen to close the macrocycle would proceed in better yield. To this end, pyrazole boronic ester **36** was synthesized in good yield, and reacted with compound **26** via a Suzuki coupling to afford **37**. From intermediate **37**, formation of the lactam ring proceeded in good yield with no modifications from the conditions described in Scheme 1. After cross coupling with indole **31A**, the resultant benzyl alcohol **39** was deprotected to form intermediate **40**. Initially, the hydroxyl group of **40** was converted to a bromide leaving group via Appel reaction (not shown). Despite replacing the weak hydroxyl nucleophile from Scheme 1 with the more nucleophilic indole nitrogen, a significant amount of elimination byproduct was still formed. However, conversion of alcohol to the tosylate **41** (Scheme 2) followed by S_N_2 displacement with the indole nitrogen provided macrocycle **42** in 76% yield, with no elimination byproduct observed. The cyclization step still proceeded with complete facial selectivity away from the (*R*)-methyl group, from milligram up to 20 g-scale. Ester saponification provided compound **13**.

Finally, we looked to improve upon the amide arylation cross coupling reaction (**30** to **32** from Scheme 1). The CuI/*trans*-*N* ^1^ *N* ^2^-dimethylaminocyclohexane catalyst system required high loadings, long reaction times, and variable yields. The near stoichiometric catalyst loadings also contributed to more difficult purifications of this step. Screening of various cross coupling catalysts and conditions identified [Pd­(cinnamyl)­Cl]_2_/^*t*^BuBrettPhos as a highly efficient catalyst, with high yields (>90%), short reaction times (<1 h), and low catalyst loadings (as low as 3 mol %). This second-generation synthesis affords final compound **13** in 39% yield over 5 steps from intermediate **38**, enabling the ready synthesis of multigram batches of **13**.

Analogs (**14–20**) of compound **13** were prepared analogously, substituting the appropriately functionalized indole in place of **31**. Compounds **19** and **20** were synthesized via a modified route (Scheme 2, bottom panel). From intermediate **38**, the benzyl protecting group was cleaved under hydrogenation conditions and the orthogonally reactive THP group was installed. Cross coupling the 5-benzyloxy indole **43** under the Buchwald conditions described for **39** provided compound **45**. THP deprotection under acidic conditions and tosylation afforded compound **46**, which was then cyclized by treating with Cs_2_CO_3_ in DMF. The resultant macrocyclic benzyloxy compound **47** was deprotected with Pd/C, Pd­(OH)_2_, and H_2_ to furnish **48**. Phenol **48** was then alkylated with a variety of electrophiles (2 examples shown), where X is a leaving group (**19** X = Br, **20** X = OTs).

## Conclusions

Here we report on a novel series of macrocyclic Mcl-1 inhibitors based on the dihydropyrazinoindolone series of inhibitors previously disclosed from our lab. This new series introduces a 5-atom tether between the 3-position of the pyrazole ring and the indole nitrogen. This macrocyclic tether constrains the indole substituent to the favored binding conformation, where the 2-carboxylic acid moiety forms a hydrogen bonding interaction with Asn260. Due to steric hindrance from the (*R*)-methyl group on the dihydropyrazinoindolone core, the cyclization proceeds with complete facial selectivity opposite the methyl group. Additionally, we found that with the macrocyclic tether in place, C5 substitution on the pyrazole was not necessary. Removal of the C5-substituent allowed for rotation around the pyrazole-indole bond, solving the previous synthetic challenge of obtaining a 1:1 mixture of noninterconverting atropisomers with nonsymmetrical indole C7 substituents (such as the trimethyl pyrazole). The SAR of the macrocyclic series was explored by varying the length and composition of the tether between the C7 pyrazole and indole nitrogen, the heterocycle at the C7 position, and modification of the indole 2-carboxylic acid substituents. These compounds exhibit superior cellular potency to the acyclic series, and compound **13** showed a significantly improved clearance to its acyclic comparator, **9**. However, introduction of the 4-methyl tetrahydropyranyl ether at the indole 5-position (**20**) failed to improve the mouse CL, despite this modification resulting in a 20-fold improvement in the acyclic series. Compound **13** was evaluated in a NSCLC A427-derived xenograft model, where it induced tumor regression as a single agent. The improved cellular potency of the macrocyclic series enabled us to replace the 2-carboxylic acid with a nonacidic moiety, a substitution that had previously resulted in a near total loss of potency. The nonacidic macrocycle **25** retained a cellular GI_50_ of 39 nM and 105 nM in H929 and A427 cells, respectively, and showed good oral bioavailability (% *F* = 22, AUC_IV_ = 136,000 nM*h, AUC_PO_ = 14,000 nM*h) on this scaffold for the first time in our program.

Despite the improvement to in vivo efficacy afforded by the macrocyclic series, progression of any Mcl-1 inhibitor to the clinic remains a challenge. Multiple Mcl-1 inhibitors have entered the clinic and resulted in troponin increases in patients,^55,58^ suggesting a mechanism-based cardiotoxicity. The potential of cardiotoxic risk from Mcl-1 inhibition is also supported by the genetic deletion of Mcl-1 in cardiomyocytes, which is lethal.^74^ In addition, treatment of hiPSC-cardiomyocytes with small molecule inhibitors also results in loss of function and cell death.^75^ Interestingly, deletion of BAK and BAX^74^ or codosing with a caspase inhibitor^75^ were insufficient to rescue normal function, indicating a nonapoptotic role of Mcl-1 in cardiac tissue. Recently, Wright et al. have implicated a role of Mcl-1 in the fatty acid oxidation through interaction with ACSL1.^76^

Other groups developing Mcl-1 inhibitors have postulated that tailoring the PK profile may provide a therapeutic window for Mcl-1 inhibition.^55,77^ For example, by employing a short half-life compound, it may be possible to achieve sufficient exposure to induce apoptosis in primed tumor cells but spare cardiac tissues. Our macrocyclic inhibitor platform allows us to maintain potency in the absence of an acidic functional group (compound **25**). This key feature allows us to explore potent, neutral Mcl-1 inhibitors; whereas, all previously reported Mcl-1 inhibitors that reached the clinic or late-stage development have contained acidic functional groups. The flexibility of the tolerated substitution patterns on our macrocyclic scaffold provides us a unique opportunity to explore a novel chemical space to identify compounds with a suitable PK profile to achieve a therapeutic window for Mcl-1 inhibition.

## Experimental Section

### Chemistry General

All NMR spectra were recorded at room temperature on a 400 MHz AMX Bruker spectrometer. ^1^H chemical shifts are reported in δ values in ppm downfield with the deuterated solvent as the internal standard. Data are reported as follows: chemical shift, multiplicity (s = singlet, d = doublet, t = triplet, q = quartet, br = broad, m = multiplet), integration, coupling constant (Hz). Low-resolution mass spectra were obtained on an Agilent 1200 series 6140 mass spectrometer with electrospray ionization. All samples were of ≥95% purity as analyzed by HPLC. Analytical HPLC was performed on an Agilent 1200 series with UV detection at 214 and 254 nm along with ELSD detection. LC/MS parameters were as follows: method 1: Phenomenex-C18 Kinetex column, 50 × 2.1 mm, 2 min gradient, 5% (0.1% TFA/MeCN)/95% (0.1% TFA/H_2_O) to 95% (0.1% TFA/MeCN)/5% (0.1% TFA/H_2_O), method 2: Phenomenex-C18 Kinetex column, 50 × 2.1 mm, 2 min gradient, 50% (0.1% TFA/MeCN)/50% (0.1% TFA/H_2_O) to 95% (0.1% TFA/MeCN)/5% (0.1% TFA/H_2_O), method 3: Phenomenex-C18 Kinetex column, 50 × 2.1 mm, 1 min gradient, 5% (0.1% TFA/MeCN)/95% (0.1% TFA/H_2_O) to 93% (0.1% TFA/MeCN)/7% (0.1% TFA/H_2_O). Preparative reverse phase purification was performed on a Gilson HPLC (Phenomenex-C18, 100 × 30 mm, 10 min gradient, 5 to 95% MeCN/H_2_O with 0.1% TFA). Normal phase purification was performed with Combi-flash Rf (plus-UV) Automated Flash Chromatography System. Solvents for reactions, extraction, and washing were ACS reagent grade, and solvents for chromatography were HPLC grade. All reagents were purchased from chemical suppliers and used without purification. Compounds **1**,^66^ **9**,^66^ **26**,^60^ **31**,^66^ **31A**,^66^ and **44** ^66^ were synthesized as previously reported. LCMS traces for tested compounds are available in Supporting Information.

### Protein Expression and Purification for Assays and X-ray Structures

Protein preparation was described previously.^59^ Briefly, a previously reported construct was subcloned into an expression vector (pDEST-HisMBP) expressed in *Escherichia coli* BL21 CodonPlus (DE3) RIL (Stratagene) and purified through nickel-column and size-exclusion chromatography sequentially.

### Protein Crystallization, Data Collection, and Structure Refinement

Structural studies were performed as previously described.^59–63^ Briefly, Mcl-1 protein (15 mg/mL) was mixed with a 1.2× excess of ligand in solution (25–30% PEG 3350, 0.1 M Bis-TRIS pH 6.5, 0.2 M MgCl_2_) by hanging drop followed by flash freezing after cryo-protection using 10–20% glycol. Data were collected at Life Sciences Collaborative Access Team (LS-CAT) 21-ID-G beamline, Advanced Photon Source (APS), Argonne National Laboratory. Indexing, integration and scaling were performed with HKL2000 (HKL Research),^78^ phasing by molecular replacement with Phaser (CCP4)^79,80^ using the structure (PDB: 9BCG) as a model, refinement used Phenix.^81^ Structural statistics are given in the Supporting Information. Figures were prepared with PyMOL (Schrödinger, LLC: New York, 2010).

### TR-FRET Assay Conditions

A fluorescein isothiocyanate (FITC)-labeled BH3 peptide derived from Bak (FITC-Bak; FITC-AHx-GQVGRQLAIIGDDINR-NH2) was purchased from Genscript and used without further purification. TR-FRET measurements were made using 384-well, white, flat-bottom plates (Corning) containing 300 nM FITC labeled BAK peptide, 1 nM Mcl-1 6HIS fusion protein, 1 nM anti 6HIS-terbium (LanthaScreen Elite Tb-anti-HIS Antibody [Thermo Fisher]) and compound incubated in a buffer containing 4.5 mM monobasic potassium phosphate, 15.5 mM dibasic potassium phosphate, 1 mM EDTA, 50 mM NaCl, 1 mM DTT, 0.05% Pluronic F-68, pH 7.5. Mixtures containing vehicle without compound served as a negative control, while mixtures containing no protein served as a positive control. The mixtures were incubated for 3 h and luminescence (Delta F) was measured on the Biotek Cytation 3 equipped with a filter cube containing an Ex 340/30 nM Em 620/10 filter and an Ex 340/30 Em 520/10 filter. The ratio of 520/620 wavelengths was used to generate the TR-FRET signal. The TR-FRET signal was plotted versus compound concentration using XLFit (IDBS) curve fitting software to generate a four-parameter sigmoidal dose response (XLfit eq 205) to obtain an IC_50_. The IC_50_ was converted to a *K*_i_ using the following equation

$$K_{i}=I_{50}/\left(L_{50}{/K}_{d}\right)+\left(P_{0}{/K}_{d}\right)+1]$$

where [*I*]_50_ is the concentration of the free inhibitor at 50% inhibition I_50_ = IC_50_ – *P*_0_ + PL_50_[1 + (*K*_d_/*L*_50_)], *L*_50_ is the concentration of free ligand at 50% inhibition, *K*_d_ is the binding constant of the Bak BH3 peptide, *P*_0_ is the free protein concentration at 0% inhibition, PL_50_ is the protein–ligand complex concentration at 50% inhibition.^82^ Two or more repeats were obtained and average *K*_*i*_ values are reported. The *Z*′-factor for the TR-FRET assay was 0.842 ± 0.05, and the *K*_i_-IC_50_ correlation is linear to IC_50_ = 2 nM/*K*_i_ = 80 pM.

### Cell Culture

NCI-H929, A427, and K562 cell lines were obtained from ATCC. NCI-H929 cells were cultured in RPMI1640 (ATCC-formulated, Catalog No. 30-2001) + 10% fetal calf serum (FCS, GIBCO BRL, Cat. No. 26140) + 0.05 mM mercaptoethanol. A-427 cells were cultured in RPMI1640 (ATCC-formulated, Catalog No. 30-2001) + 10% fetal calf serum (FCS, GIBCO BRL, Cat. No. 26140). K562 cell lines were cultured in Iscove’s Modified Dulbecco Medium (Gibco-formulated, purchased from Thermo-Fisher, Cat. No. 12440-053) + 10% fetal bovine serum (FBS, Sigma-Aldrich, F2442). All cell lines were cultured at 37 °C with 5% CO_2_ in a humidified incubator and have been regularly tested negatively for mycoplasm contamination.

### In Vitro Proliferation Assay

Cells were seeded at 750–1500 cells per well (depending on growth kinetics) in 384-well plates (Corning, Catalog No. 3707) or at 3000 cells per well in 96-well plates in the respective growth medium and allowed to settle overnight. Suspension cells were plated immediately before compound addition. Adherent cell lines were incubated overnight at 37 °C in a tissue culture incubator prior to compound addition. Compounds were added in triplicates using an HP D300e Digital Dispenser (Hewlett-Packard) or manually at the concentrations indicated and total volumes were normalized using DMSO backfill, with final DMSO concentration of 0.5%. Control wells were treated with DMSO only. Following incubation for 72 or 96 h, a Cell Titer-Glo assay was conducted following the manufacturer recommendations (Promega, G9243) and luminescence was measured using a Victor X5 (PerkinElmer) or a Cytation 3 (Biotek) plate reader. % Viability was defined as relative luminescence units (RLU) of each well divided by the RLU of cells on day 0. Four parameter sigmoidal dose–response curves were generated and IC_50_ values were determined using XLFit (IDBS) software (eq 205) or in-house software. Drug combination experiments were performed in 96-well or 384-well format and drug synergy was determined using the BLISS model (PMID: 26171228).

### Caspase 3/7 Induction Assays

Cells were dispensed in 96-well plates (Thermo Scientific #136101) with cell culture medium RPMI 1640 containing 5% FBS, cell concentration of 100,000 cells per mL, and a volume of 100 μL per well. Cells were treated with a 10-point compound titration and incubated at 37 °C for 3 h. The final concentration ranged from 3125 nM to 24 nM in 0.5% DMSO. At 3 h, 50 μL of Caspase-Glo (Promega #G8090) was added to each well, and the mixture was incubated at room temperature in the dark for 1 h. Luminescence was measured (Biotek Cytation 3) and analyzed using GraphPad Prism to generate EC_50_ values.

### In Vivo Xenograft Experiments

Female BomTac:NMRI-Foxn1nu mice and CB17/Icr-Prkdc­(scid)/IcrCrl and were obtained from Taconic Denmark at an age of 6–8 weeks. After arrival of the local animal facility at Boehringer Ingelheim RCV GmbH & Co KG (institutional approval—Amt der Wiener Landesregierung, Magistratsabteilung 58, GZ: 135147/2013/4) mice were allowed to adjust to housing conditions at least for 5 days before the start of the experiment. Mice were group-housed under pathogen-free and controlled environmental conditions and handled according to the institutional, governmental and European Union guidelines (Austrian Animal Protection Laws, GV-SOLAS and FELASA guidelines). Animal studies were approved by the internal ethics committee and the local governmental committee (Amt der Wiener Landesregierung, Magistratsabteilung 58). To establish subcutaneous tumors mice were injected with 5 × 10^6^ A427 cells in Matrigel (CB17/Icr-Prkdc­(scid)/IcrCrl. Tumor diameters were measured with a caliper three times a week. The volume of each tumor [in mm^3^] was calculated according to the formula “tumor volume = length*diameter^2^*π/6.” To monitor side effects of treatment, mice were inspected daily for abnormalities and body weight was determined. Animals were sacrificed when the tumors reached a size of 1500 mm^3^. Mice were dispatched randomly into treatment groups when the tumor size was 160 mm^3^. Tumors were reported as regressing when the tumor volume at a given day was below the tumor volume at treatment start.

### Pharmacokinetic Analyses

For PK analysis, mice were administered intravenously or orally with compound formulated in 10% ethanol and 10% Cremophor EL. Compound **13** dosing was also screened with 20% Phosphal IPG, 20% Phosphal MCT, 10% HP-β-CD, and 40% HP-β-CD. Plasma samples were obtained at predefined time points and compound concentrations in plasma were measured by quantitative HPLC–MS/MS using an internal standard. Calibration and quality control samples were prepared using blank plasma from untreated animals. Samples were precipitated with acetonitrile and injected into a HPLC system (Agilent 1200). Separation was performed by gradients of 5 mmol/L ammonium acetate pH 5.0 and acetonitrile with 0.1% formic acid on a Luna C8 reversed-phase column with 2.5 μm particles (Phenomenex). The HPLC was interfaced by ESI operated in positive ionization mode to a triple quadrupole mass spectrometer (6500+ Triple Quad System, SCIEX) operated in multiple reaction monitoring mode. Chromatograms were analyzed with Analyst (SCIEX) and pharmacokinetic parameters were calculated by noncompartmental analysis using proprietary software.

## General Procedures

### General Procedure **A**

#### Ullmann Cross Coupling

In a reaction vessel, the (*R*)-methyl-dihydropyrazinoindolone core (e.g., **30**, **38**, or **43**) (1.0 equiv), 7-bromoindole or 7-iodoindole (1.5 equiv), CuI (0.5 equiv), (*trans*)-1,2-*N*,*N*′-dimethylaminocyclohexane (0.5 equiv), and K_2_CO_3_ (2.0 equiv) were weighed. The reaction vessel was charged with toluene (0.4 M) and sparged with argon for 5 min. The vessel was then sealed and heated to 100 °C for 48 h. The reaction was cooled to room temperature and diluted with 1:1 EtOAc/H_2_O. The aqueous layer was separated and extracted with EtOAc (2×). The combined organic layers were washed with sat. NH_4_Cl, sat. NaHCO_3_, water, and brine. The organic layer was dried over MgSO_4_, filtered, and concentrated. The crude residue was purified by flash column chromatography eluting with EtOAc/hexanes to afford the desired compound.

### General Procedure **B**

#### Buchwald Cross Coupling

In a reaction vessel, the (*R*)-methyl-dihydropyrazinoindolone core (e.g., **30**, **38**, or **43**) (1.0 equiv), 7-bromoindole or 7-iodoindole (1.2 equiv), [Pd­(cinnamyl)­Cl]_2_ (0.05 equiv), ^*t*^Bu-BrettPhos (0.1 equiv), and Cs_2_CO_3_ (4.0 equiv) were weighed. The reaction vessel was charged with toluene (0.2 M) and sparged with argon for 5 min. The reaction was sealed and heated to 100 °C for 2–24 h. The reaction was cooled to room temperature and diluted with 1:1 EtOAc/H_2_O. The aqueous layer was separated and extracted with EtOAc (2×). The combined organic layers were washed with sat. NH_4_Cl, sat. NaHCO_3_, water, and brine. The organic layer was dried over MgSO_4_, filtered, and concentrated. The crude residue was purified by flash column chromatography eluting with EtOAc/hexanes to afford the desired compound.

### General Procedure **C**

#### Hydrogenolysis of Benzyl Ether

In a reaction vessel, the benzylic ether (e.g., **32**, **38**, **39**, **47**) (1.0 equiv) was dissolved in THF/^*i*^PrOH (3:1) and the resultant mixture was sparged with argon for 5 min. Pd/C (10% wt., 0.1 equiv) and Pd­(OH)_2_/C (20% wt., 0.1 equiv) were added to the reaction vessel, and the reaction was flushed with argon. The reaction mixture was allowed to stir under an atmosphere of H_2_ at 40 °C until complete by LCMS. The reaction mixture was filtered through a pad of Celite, rinsed with DCM, and concentrated. The crude residue was used without further purification.

### General Procedure **D**

#### Tosylation of Alcohol

In a reaction vessel, the alcohol (e.g., **40**) (1.0 equiv) was dissolved in DCM (0.08 M). TEA (10 equiv) and DMAP (0.10 equiv) were added and the reaction was stirred for 5 min at RT. The reaction was cooled to 0 °C and *p*-toluenesulfonic anhydride (4.0 equiv) was added. The reaction was allowed to stir at 0 °C for 10 min and then warmed to room temperature for 2 h. The reaction was extracted with DCM, washed with brine, dried over MgSO_4_, filtered, and concentrated. The crude residue was purified by flash column chromatography eluting with 0 to 100% EtOAc in hexanes to afford the desired product.

### General Procedure **E**

#### Macrocyclization

In a reaction vessel, the tosylate (e.g., **41**, **46**) was dissolved in DMF (0.01 M) under an atmosphere of argon. Cs_2_CO_3_ (3.0 equiv) was added, and the reaction was heated to 60 °C and stirred for 16 h. The reaction was extracted with EtOAc, washed with H_2_O, washed with brine, dried over MgSO_4_, filtered, and concentrated. The crude residue was purified by flash column chromatography eluting with 0 to 50% 95:5 EtOAc/MeOH in hexanes to afford the desired product.

### General Procedure **F**

#### Saponification of Indole Ester

In a reaction vessel, the ester (e.g., **42**, **49**) was dissolved in THF/MeOH/H_2_O (5:1:1, 0.2 M). LiOH (10 equiv) was added, and the reaction was heated at 50 °C for 3–24 h until the LCMS shows complete conversion. The reaction was extracted with DCM, acidified with 1 M HCl, washed with H_2_O, washed with brine, dried over MgSO_4_, filtered, and concentrated. The crude residue was purified by reverse phase HPLC eluting with MeCN/H_2_O with 0.1% TFA additive. The resultant compound was concentrated, dissolved in DCM, washed with aq NaHCO_3_, dried with MgSO_4_, filtered, and concentrated to afford the desired product.

### Synthesis of Intermediates

#### 3-((Benzyloxy)­methyl)-1,5-dimethyl-4-(4,4,5,5-tetramethyl-1,3,2-dioxaborolan-2-yl)-1*H*-pyrazole (**27**)

##### Step A: 3-((Benzyloxy)­methyl)-4-bromo-1,5-dimethyl-1*H*-pyrazole

(1,5-Dimethyl-1*H*-pyrazol-3-yl)­methanol (840 mg, 6.67 mmol, 1.0 equiv) was dissolved in DMF (15 mL) and cooled to 0 °C. NBS (1.4 g, 7.86 mmol, 1.15 equiv) was added and the reaction was allowed to stir at room temperature for 1 h. The reaction was diluted into DCM (50 mL), washed with saturated aq Na_2_S_2_O_3_, brine, and then dried with MgSO_4_, filtered, and concentrated. LCMS (ESI) method 1: RT = 0.799 min, *m*/*z* = 205.0 [M + H]. The crude residue was taken up in DMF (15 mL) and cooled to 0 °C. Sodium hydride (300 mg, 1.87 mmol, 1.9 equiv) was added, and the reaction was allowed to stir for 15 min. Benzyl bromide (1.5 g, 8.8 mmol, 1.3 equiv) was added, and the reaction was allowed to stir at room temperature until complete by LCMS analysis. The reaction was quenched with H_2_O (10 mL), extracted with DCM, washed with H_2_O, brine, dried over MgSO_4_, filtered, and concentrated. The crude residue was purified by flash column chromatography eluting with 0 to 100% EtOAc in hexanes to afford the title compound (1.72 g, 87% yield). LCMS (ESI) method 1: RT = 1.594 min, *m*/*z* = 295.0 [M + H].

##### Step B: 3-((Benzyloxy)­methyl)-1,5-dimethyl-4-(4,4,5,5-tetramethyl-1,3,2-dioxaborolan-2-yl)-1*H*-pyrazole

3-((Benzyloxy)­methyl)-4-bromo-1,5-dimethyl-1*H*-pyrazole (1.6 g, 5.4 mmol, 1.0 equiv) was dissolved in THF (40 mL) under an atm. of argon. The reaction was cooled to −78 °C. *n*-BuLi (2.5 M, 2.6 mL, 6.5 mmol, 1.2 equiv) was added, and the reaction was allowed to stir for 1 h. Bis­(pinacalato)­diboron (1.5 g, 8.0 mmol, 1.5 equiv) was added, and the reaction was allowed to stir at room temperature for 5 h. The reaction was concentrated, dissolved in EtOAc, filtered through a pad of Celite, and concentrated. The crude residue was purified by flash column chromatography eluting with 0 to 100% EtOAc in hexanes to afford the title compound (1.52 g, 82% yield). LCMS (ESI) method 1: RT = 1.802 min, *m*/*z* = 343.1 [M + H]^+^.

#### 3-((3-(Benzyloxy)­propoxy)­methyl)-1-methyl-4-(4,4,5,5-tetramethyl-1,3,2-dioxaborolan-2-yl)-1*H*-pyrazole (**36**)

##### Step A: 3-((3-(Benzyloxy)­propoxy)­methyl)-4-bromo-1-methyl-1*H*-pyrazole

In a dry round-bottom (4-bromo-1-methyl-1*H*-pyrazol-3-yl)­methanol (5.00 g, 26.2 mmol, 1.0 equiv) was dissolved in DMF (32 mL). The reaction mixture was cooled to 0 °C and NaH (1.67 g, 41.9 mmol, 1.6 equiv) was added. The reaction was allowed to stir for 20 min. ((3-Bromopropoxy)­methyl)­benzene (7.40 mL, 41.90 mmol, 1.6 equiv) was then added, and the reaction was allowed to stir for 16 h at room temperature. Upon completion, the reaction was cooled to 0 °C, quenched with MeOH (20 mL), and diluted into H_2_O (200 mL). The reaction was extracted with EtOAc (3 × 100 mL), washed with brine, dried over MgSO_4_, filtered, and then concentrated. The crude product was purified by flash column chromatography eluting with 0 to 70% EtOAc in hexanes to afford the title compound (5.78 g, 65% yield). LCMS (ESI) method 3: RT = 1.01 min, *m*/*z* = 339.3 [M + H]^+^.

##### Step B: 3-((3-(Benzyloxy)­propoxy)­methyl)-1-methyl-4-(4,4,5,5-tetramethyl-1,3,2-dioxaborolan-2-yl)-1*H*-pyrazole

In a dry round-bottomed flask, 3-((3-(benzyloxy) propoxy)­methyl)-4-bromo-1-methyl-1*H*-pyrazole (5.78 g, 17.1 mmol, 1.0 equiv) was dissolved in anhydrous THF (85 mL) and cooled to −78 °C. 2-Isopropoxy-4,4,5,5-tetramethyl-1,3,2-dioxaborolane (10.5 mL, 51.3 mmol, 3 equiv) was added, followed by addition of *n*-BuLi (11.7 mL, 18.7 mmol, 1.1 equiv) over 20 min. The reaction was allowed to stir for 1 h at −78 °C, then quenched with MeOH. The reaction was extracted with EtOAc, washed with brine, dried over MgSO_4_, filtered, and concentrated. The crude product was purified by flash column chromatography eluting with 0 to 100% EtOAc in hexanes to afford the title compound (6.6 g, quantitative yield). LCMS (ESI) method 3: RT = 1.13 min, *m*/*z* = 387.4 [M + H]^+^.

#### (*R*)-6-(3-((3-(benzyloxy)­propoxy)­methyl)-1-methyl-1*H*-pyrazol-4-yl)-7-chloro-10-(3-(4-chloro-3,5-dimethylphenoxy)­propyl)-4-methyl-3,4-dihydropyrazino­[1,2-*a*]­indol-1­(2*H*)-one (**38**)

##### Step A: Ethyl (*R*)-7-(3-((3-(Benzyloxy)­propoxy)­methyl)-1-methyl-1*H*-pyrazol-4-yl)-1-(1-((*tert*-butoxycarbonyl)­amino)­propan-2-yl)-6-chloro-3-(3-(4-chloro-3,5-dimethylphenoxy) propyl)-1*H*-indole-2-carboxylate (**37**)

In a heavy wall vial, **26** (5.67 g, 11.4 mmol, 1 equiv), **36** (5.94 g, 15.3 mmol, 1.35 equiv), K_2_CO_3_ (4.72 g, 34.2 mmol, 3 equiv), and Pd­(PPh_3_)_4_ (1.30 g, 3.4 mmol, 0.1 equiv) were added and dissolved in dioxane (90 mL) and H_2_O (20 mL). The solution was sparged with argon for 5 min, and the reaction was then sealed and heated to 100 °C for 8 h. The reaction was then cooled to room temperature, extracted with EtOAc, dried over MgSO_4_ and then concentrated. The crude material was purified by flash column chromatography, eluting with 0 to 100% EtOAc in hexanes to afford the title compound (8 g, 77% yield). LCMS (ESI) method 2: RT = 1.19 min, *m*/*z* = 678.6 [M + H]^+^.

##### Step B: (*R*)-6-(3-((3-(Benzyloxy)­propoxy)­methyl)-1-methyl-1*H*-pyrazol-4-yl)-7-chloro-10-(3-(4-chloro-3,5-dimethylphenoxy)­propyl)-4-methyl-3,4-dihydropyrazino­[1,2-*a*]­indol-1­(2*H*)-one (**38**)

In a round-bottomed flask, compound **37** (8 g, 11.8 mmol, 1 equiv) was dissolved in MeCN (60 mL). *tert*-Butyl (*S*)-5-methyl-1,2,3-oxathiazolidine-3-carboxylate 2,2-dioxide (4.19 g, 17.6 mmol, 1.5 equiv) and Cs_2_CO_3_ (8.45 g, 25.9 mmol, 2.2 equiv) were added, and the reaction was heated to 70 °C for 16 h after which time the reaction was determined to be complete. The reaction mixture was concentrated, extracted with EtOAc, washed with H_2_O, dried over MgSO_4_, filtered, and concentrated. The product was dissolved in DCM (60 mL). Trifluoroacetic acid (9 mL) was added, and the reaction was allowed to stir at room temperature for 3 h. The reaction was concentrated, and the crude residue was dissolved in ethanol (50 mL). Potassium carbonate (8.2 g, 59 mmol, 5.0 equiv) was added, and the reaction was heated to 60 °C for 2 h. The solvent was removed and the crude residue was extracted with EtOAc, washed with brine, dried over MgSO_4_, filtered, and concentrated. The crude product was purified by flash column chromatography eluting with 0 to 5% MeOH in DCM to afford the title compound (4.1 mg, 50% yield over 3 reactions). LCMS (ESI) method 2: RT = 1.06 min, *m*/*z* = 689.6 [M + H]^+^.

#### (4*R*)-7-Chloro-10-(3-(4-chloro-3,5-dimethylphenoxy)­propyl)-4-methyl-6-(1-methyl-3-((3-((tetrahydro-2*H*-pyran-2-yl)­oxy)­propoxy)­methyl)-1*H*-pyrazol-4-yl)-3,4-dihydropyrazino [1,2-*a*]­indol-1­(2*H*)-one (**43**)

##### Step A: (*R*)-7-Chloro-10-(3-(4-chloro-3,5-dimethylphenoxy)­propyl)-6-(3-((3-hydroxy propoxy)­methyl)-1-methyl-1*H*-pyrazol-4-yl)-4-methyl-3,4-dihydropyrazino­[1,2-*a*]­indol-1­(2*H*)-one

Compound **38** (200 mg, 0.29 mmol) was dissolved in THF/^*i*^PrOH (8 mL, 3:1). The reaction was sparged with argon, followed by addition of Pd/C (10 wt %, 45 mg, 0.045 mmol, 0.15 equiv) and Pd­(OH)_2_/C (20% wt., 31 mg, 0.045 mmol, 0.15 equiv). The vessel was evacuated and backfilled with argon (3×), followed by introduction of H_2_. The reaction was allowed to stir under a balloon atmosphere of H_2_ at 35 °C for 2 h until no starting material was detected by LCMS. Upon completion, the reaction was filtered through a plug of Celite and rinsed with DCM. The filtrate was concentrated to afford the desired product (170 mg, 98% yield) and used without further purification. LCMS (ESI) method 2: RT = 1.029 min, *m*/*z* = 599.0 [M + H].

##### Step B: (4*R*)-7-Chloro-10-(3-(4-chloro-3,5-dimethylphenoxy)­propyl)-4-methyl-6-(1-methyl-3-((3-((tetrahydro-2*H*-pyran-2-yl)­oxy)­propoxy)­methyl)-1*H*-pyrazol-4-yl)-3,4-dihydro pyrazino­[1,2-*a*]­indol-1­(2*H*)-one (**43**)

The product from step A (100 mg, 0.17 mmol, 1.0 equiv) was dissolved in DCM (1 mL). 3,4-Dihydro-2H-pyran (56 mg, 0.66 mmol, 4.0 equiv) and PPTS (12 mg, 0.048 mmol, 0.3 equiv) were added and the reaction was allowed to stir at room temperature for 20 h. The reaction was extracted with EtOAc, washed with NaHCO_3_, dried over Na_2_SO_4_, filtered, and concentrated. The crude material was purified by flash column chromatography eluting with 0 to 10% MeOH in DCM to afford the title compound (85 mg, 75% yield). LCMS (ESI) method 2: RT = 1.603 min, *m*/*z* = 705.0 [M + N*a*].

#### Ethyl (2^6^3^4^ *S*_*a*_,1^7^2^2^ *R*_*a*_,2^4^ *R*)-2^7^-Chloro-2^10^-(3-(4-chloro-3,5-dimethylphenoxy)­propyl)-1^5^-hydroxy-1^4^-methoxy-2^4^,3^1^-dimethyl-2^1^-oxo-2^1^,2^2^,2^3^,2^4^-tetrahydro-1^1^ *H*,3^1^ *H*-5-oxa-2­(2,6)­pyrazino [1,2-*a*]­indola-1­(7,1)­indola-3­(4,3)­pyrazolacyclooctaphane-12-carboxylate (**48**)

##### Step A: Ethyl 5-(Benzyloxy)-7-((4*R*)-7-chloro-10-(3-(4-chloro-3,5-dimethylphenoxy)­propyl)-4-methyl-6-(1-methyl-3-((3-((tetrahydro-2*H*-pyran-2-yl)­oxy)­propoxy)­methyl)-1*H*-pyrazol-4-yl)-1-oxo-3,4-dihydropyrazino­[1,2-*a*]­indol-2­(1*H*)-yl)-4-methoxy-1*H*-indole-2-carboxylate (**45**)

The title compound (4.14 g, 56% yield) was prepared following general procedure B using **43** (5.04 g, 7.37 mmol, 1.0 equiv) and compound **44** (7.2 g, 16.0 mmol, 2.2 equiv), palladium π-cinnamyl chloride dimer (400 mg, 0.77 mmol, 0.10 equiv), *t*BuBrettPhos (400 mg, 0.825 mmol, 0.11 equiv), and Cs_2_CO_3_ (11.9 g, 37 mmol, 5.0 equiv) and allowing the reaction to stir 30 h. The crude residue was purified by flash column chromatography eluting with 0 to 100% EtOAc in hexanes. LCMS (ESI) method 2: RT = 2.027 min, *m*/*z* = 922.0 [M + H-THP]^+^.

##### Step B: Ethyl (*R*)-5-(Benzyloxy)-7-(7-chloro-10-(3-(4-chloro-3,5-dimethylphenoxy)­propyl)-6-(3-((3-hydroxypropoxy)­methyl)-1-methyl-1*H*-pyrazol-4-yl)-4-methyl-1-oxo-3,4-dihydropyrazino­[1,2-*a*]­indol-2­(1*H*)-yl)-4-methoxy-1*H*-indole-2-carboxylate

Compound **45** (4.14 g, 4.11 mmol, 1.0 equiv) was dissolved in MeOH (120 mL) and THF (30 mL) at room temperature. Tosic acid monohydrate (250 mg, 1.31 mmol, 0.32 equiv) was added, and the reaction was allowed to stir at room temperature for 2 h. The reaction was extracted with EtOAc, washed with H_2_O, dried over MgSO_4_, filtered, and concentrated. The crude residue was purified by flash column chromatography eluting with 0 to 10% MeOH in DCM to afford the title compound (3.84 g, quant. yield). LCMS (ESI) method 2: RT = 1.751 min, 1.805 min, *m*/*z* = 922.0 [M + H]^+^.

##### Step C: Ethyl (*R*)-5-(Benzyloxy)-7-(7-chloro-10-(3-(4-chloro-3,5-dimethylphenoxy)­propyl)-4-methyl-6-(1-methyl-3-((3-(tosyloxy)­propoxy)­methyl)-1*H*-pyrazol-4-yl)-1-oxo-3,4-dihydro pyrazino­[1,2-*a*]­indol-2­(1*H*)-yl)-4-methoxy-1*H*-indole-2-carboxylate (**46**)

The title compound (1.80 g, 77% yield) was prepared following general procedure D using the product from step B (2.0 g, 2.17 mmol, 1.0 equiv). The crude product was purified by flash column chromatography eluting with 0 to 100% 95:5 EtOAc/MeOH in hexanes. LCMS (ESI) method 2: RT = 1.970 min, *m*/*z* = 1075.8 [M + H]^+^.

##### Step D: Ethyl (2^6^3^4^ *S*_*a*_,1^7^2^2^ *R*_*a*_,2^4^ *R*)-1^5^-(Benzyloxy)-2^7^-chloro-2^10^-(3-(4-chloro-3,5-dimethylphenoxy)­propyl)-1^4^-methoxy-2^4^,3^1^-dimethyl-2^1^-oxo-2^1^,2^2^,2^3^,2^4^-tetrahydro-1^1^ *H*,3^1^ *H*-5-oxa-2­(2,6)-pyrazino­[1,2-*a*]­indola-1­(7,1)-indola-3­(4,3)-pyrazolacyclooctaphane-1^2^-carboxylate (**47**)

The title compound (1.20 g, 79% yield) was prepared following general procedure E using compound **46** (1.80 g, 1.67 mmol, 1.0 equiv). The crude product was purified by flash column chromatography eluting with 0 to 100% EtOAc in hexanes. LCMS (ESI) method 2: RT = 2.069 min, *m*/*z* = 904.0 [M + H]^+^.

##### Step E: Ethyl (2^6^3^4^ *S*_*a*_,1^7^2^2^ *R*_*a*_,2^4^ *R*)-2^7^-Chloro-2^10^-(3-(4-chloro-3,5-dimethylphenoxy) propyl)-1^5^-hydroxy-1^4^-methoxy-2^4^,3^1^-dimethyl-2^1^-oxo-2^1^,2^2^,2^3^,2^4^-tetrahydro-1^1^ *H*,3^1^ *H*-5-oxa-2­(2,6)-pyrazino­[1,2-*a*]­indola-1­(7,1)-indola-3­(4,3)-pyrazolacyclooctaphane-1^2^-carboxylate (**48**)

The title compound (1.05 g, 97% yield) was prepared following general procedure C using compound **47** (1.20 g, 1.32 mmol, 1.0 equiv). The crude material was used without further purification. LCMS (ESI) method 2: RT = 1.664 min, *m*/*z* = 814.0 [M + H]^+^.

### Synthesis of Final Compounds

#### (*R*)-7-(7-Chloro-10-(3-(4-chloro-3,5-dimethy|phenoxy)­propyl)-4-methyl-1-oxo-6-(1,3,5-trimethyl-1*H*-pyrazol-4-yl)-3,4-dihydropyrazino|1,2-*a*|indol-2­(1*H*)-yl)-1-(2-methoxyethyl)-5-methyl-1*H*-indole-2-carboxylic Acid (**8**)

##### Step A. Methyl 7-((4*R*)-7-Chloro-10-(3-(4-chloro-3,5-dimethylphenoxy)­propyl)-4-methyl-1-oxo-6-(1,3,5-trimethyl-1*H*-pyrazol-4-yl)-3,4-dihydropyrazino­[1,2-*a*]­indol-2­(1*H*)-yl)-5-methyl-1*H*-indole-2-carboxylate

The title compound (110 mg, 82% yield) was prepared following general procedure A using (4*R*)-7-chloro-10-(3-(4-chloro-3,5-dimethylphenoxy)­propyl)-4-methyl-6-(1,3,5-trimethyl-1*H*-pyrazol-4-yl)-3,4-dihydropyrazino [1,2-*a*]­indol-1­(2*H*)-one^53^ (100 mg, 0.186 mmol, 1.0 equiv) and methyl 7-bromo-5-methyl-1*H*-indole-2-carboxylate (100 mg, 0.37 mmol, 2.0 equiv).

##### Step B. Methyl 7-((4*R*)-7-Chloro-10-(3-(4-chloro-3,5-dimethylphenoxy)­propyl)-4-methyl-1-oxo-6-(1,3,5-trimethyl-1*H*-pyrazol-4-yl)-3,4-dihydropyrazino­[1,2-*a*]­indol-2­(1*H*)-yl)-1-(2-methoxyethyl)-5-methyl-1*H*-indole-2-carboxylate

In a reaction vessel, the product from step A (70 mg, 0.10 mmol, 1.0 equiv) was dissolved in DMF (2 mL). Cs_2_CO_3_ (94 mg, 0.29 mmol, 3.0 equiv) was added, followed by 2-bromoethyl methyl ether (20 mg, 0.14 mmol, 1.5 equiv). The reaction was heated to 70 °C and allowed to stir for 3 h. The reaction was extracted with DCM, washed with H_2_O, dried over MgSO_4_, filtered, and concentrated. The crude residue was purified by flash column chromatography eluting with 0 to 100% EtOAc in hexanes.

##### Step C: (*R*)-7-(7-Chloro-10-(3-(4-chloro-3,5-dimethy|phenoxy)­propyl)-4-methyl-1-oxo-6-(1,3,5-trimethyl-1*H*-pyrazol-4-yl)-3,4-dihydropyrazino|1,2-*a*|indol-2­(1*H*)-yl)-1-(2-methoxyethyl)-5-methyl-1*H*-indole-2-carboxylic Acid (**8**)

The title compound (35 mg, 61% yield over steps B and C) was prepared following general procedure F using the product from step B. LCMS: RT = 2.06 min, MS (ES) 770 (M + H); H′ NMR (400 MHz, DMSO-*d*_6_): δ 7.76 (d, *J* = 8.4 Hz, 1H), 7.50 (s, 0.5H), 7.45 (s, 0.5H), 7.33 (m, 1H), 7.27 (m, 1H), 6.96 (s, 0.5H), 6.94 (s, 0.5H), 6.71 (s, 1H), 6.70 (s, 1H), 4.94 (m, 1H), 4.68 (m, 2H), 4.43 (m, 2H), 4.18 (m, 1H), 3.99 (t, *J* = 5.5 Hz, 2H), 3.79 (s, 1.5H), 3.77 (s, 1.5H), 3.71 (m, 1H), 3.35 (m, 2H), 3.22 (m, 2H), 3.00 (s, 1.5H), 2.95 (s, 1.5H), 2.39 (s, 1.5H), 2.36 (s, 1.5H), 2.24 (s, 6H), 2.14 (m, 1H), 1.88–2.07 (m, 5H), 1.17 (d, *J* = 6.4 Hz, 1.5H), 1.06 (d, *J* = 6.4 Hz, 1.5H).

#### (2^6^3^4^ *S*_*a*_,1^7^2^2^ *R*_*a*_,2^4^ *R*)-2^7^-Chloro-2^10^-(3-(4-chloro-3,5-dimethylphenoxy)­propyl)-1^4^,1^5^-dimethoxy-2^4^,3^1^,3^5^-trimethyl-2^1^-oxo-2^1^,2^2^,2^3^,2^4^-tetrahydro-1^1^ *H*,3^1^ *H*-5-oxa-2­(2,6)-pyrazino­[1,2-*a*]­indola-1­(7,1)-indola-3­(4,3)-pyrazolacyclooctaphane-1^2^-carboxylic Acid (**10**)

##### Step A: Ethyl 7-(3-((Benzyloxy)­methyl)-1,5-dimethyl-1*H*-pyrazol-4-yl)-6-chloro-3-(3-(4-chloro-3,5-dimethylphenoxy)­propyl)-1*H*-indole-2-carboxylate (**28**)

In a microwave vial, compound **26** (3.5 g, 7.0 mmol, 1.0 equiv), compound **27** (3.6 g, 10.5 mmol, 1.5 equiv), K_2_CO_3_ (2.9 g, 21.0 mmol, 3.0 equiv), and Pd­(PPh_3_)_4_ (560 mg, 0.07 mmol, 0.07 equiv) were combined and dissolved in dioxane (60 mL) and H_2_O (15 mL). The reaction was sparged with argon for 5 min and then the reaction was irradiated in the microwave at 140 °C for 30 min. The reaction was extracted with EtOAc, washed with H_2_O, dried over MgSO_4_, filtered, and concentrated. The crude residue was purified by flash column chromatography eluting with 0 to 100% EtOAc in hexanes to afford the title compound (3.5 g, 79% yield).

##### Step B: Ethyl (*R*)-7-(3-((Benzyloxy)­methyl)-1,5-dimethyl-1*H*-pyrazol-4-yl)-1-(1-((*tert*-butoxycarbonyl)­amino)­propan-2-yl)-6-chloro-3-(3-(4-chloro-3,5-dimethylphenoxy)­propyl)-1*H*-indole-2-carboxylate

Compound **28** (1.30 g, 2.05 mmol, 1.0 equiv) and compound **29** (778 mg, 3.28 mmol, 1.6 equiv) were dissolved in MeCN (18 mL). Cesium carbonate (1.33 g, 4.1 mmol, 2.0 equiv) was added, and the reaction was stirred at 80 °C for 24 h. The crude reaction was extracted with EtOAc, washed with H_2_O, dried over MgSO_4_, filtered, and concentrated. The crude residue was purified by flash column chromatography eluting with 0 to 100% EtOAc in hexanes to afford the title compound (1.62 g, quant. yield).

##### Step C: (*R*)-6-(3-((Benzyloxy)­methyl)-1,5-dimethyl-1*H*-pyrazol-4-yl)-7-chloro-10-(3-(4-chloro-3,5-dimethylphenoxy)­propyl)-4-methyl-3,4-dihydropyrazino­[1,2-*a*]­indol-1­(2*H*)-one (**30**)

The product from step B (1.62 g, 2.05 mmol, 1.0 equiv) was dissolved in DCM (10 mL) and TFA (1.6 mL) was added. The reaction was allowed to stir for 2 h. The crude residue was taken up in MeOH (10 mL), and K_2_CO_3_ (5.65 g, 40.9 mmol, 20 equiv) was added. The reaction was stirred at RT for 60 h. The reaction mixture was extracted with DCM, washed with aq NH_4_Cl, dried over MgSO_4_, filtered, and concentrated. The crude residue was purified by flash column chromatography to afford **30** (1.0 g, 76% yield).

##### Step D: Ethyl (*R*)-7-(6-(5-((Benzyloxy)­methyl)-1,3-dimethyl-1*H*-pyrazol-4-yl)-7-chloro-10-(3-(4-chloro-3,5-dimethylphenoxy)­propyl)-4-methyl-1-oxo-3,4-dihydropyrazino­[1,2-*a*]­indol-2­(1*H*)-yl)-4,5-dimethoxy-1*H*-indole-2-carboxylate (**32**)

The title compound was prepared following general procedure A using compounds **30** (780 mg 1.21 mmol) and **31** (906 mg, 2.42 mmol). The crude residue was purified by reverse phase HPLC to afford **32** (302 mg, 28% yield). LCMS (ESI) method 2: RT = 1.912, *m*/*z* = 891.9 (M + H), and the undesired atropisomer (312 mg, 29% yield, LCMS (ESI) method 2: RT = 1.856 min, *m*/*z* = 891.9 (M + H)) was also isolated separately.

##### Step E: Ethyl (*R*)-7-(7-Chloro-10-(3-(4-chloro-3,5-dimethylphenoxy)­propyl)-6-(5-(hydroxymethyl)-1,3-dimethyl-1*H*-pyrazol-4-yl)-4-methyl-1-oxo-3,4-dihydropyrazino­[1,2-*a*]­indol-2­(1*H*)-yl)-4,5-dimethoxy-1*H*-indole-2-carboxylate (**33**)

Compound **33** (216 mg, 79% yield) was prepared following general procedure C using compound **32** (302 mg, 0.34 mmol). LCMS (ESI) method 2: RT = 1.173 min, MS (ES) 801.9 (M + H).

##### Step F: Ethyl (2^6^3^4^ *S*_*a*_,1^7^2^2^ *R*_*a*_,2^4^ *R*)-2^7^-Chloro-2^10^-(3-(4-chloro-3,5-dimethylphenoxy) propyl)-1^4^,1^5^-dimethoxy-2^4^,3^1^,3^5^-trimethyl-2^1^-oxo-2^1^,2^2^,2^3^,2^4^-tetrahydro-1^1^ *H*,3^1^ *H*-5-oxa-2­(2,6)-pyrazino­[1,2-*a*]­indola-1­(7,1)-indola-3­(4,3)-pyrazolacyclooctaphane-1^2^-carboxylate (**34**)

Compound **33** (110 mg, 0.137 mmol) was dissolved in DMF (1.5 mL). Cs_2_CO_3_ (89.3 mg, 0.274 mmol) and 1,3-dibromopropane (36.0 mg, 18.0 μL, 0.178 mmol) were added, followed by stirring at 40 °C for 30 h. The reaction was extracted with EtOAc, washed with H_2_O, dried over Na_2_SO_4_, filtered, and concentrated. The crude residue was purified by flash column chromatography eluting with 0 to 100% EtOAc in hexanes to afford **34** (115 mg, 91% yield). LCMS (ESI) method 2: RT = 1.732 min, MS (ES) 921.9 (M + H).

##### Step G: (2^6^3^4^ *S*_*a*_,1^7^2^2^ *R*_*a*_,2^4^ *R*)-2^7^-Chloro-2^10^-(3-(4-chloro-3,5-dimethylphenoxy)­propyl)-1^4^,1^5^-dimethoxy-2^4^,3^1^,3^5^-trimethyl-2^1^-oxo-2^1^,2^2^,2^3^,2^4^-tetrahydro-1^1^ *H*,3^1^ *H*-5-oxa-2­(2,6)-pyrazino­[1,2-*a*]­indola-1­(7,1)-indola-3­(4,3)-pyrazolacyclooctaphane-1^2^-carboxylic Acid (**10**)

Sodium hydride (3.2 mg, 0.131 mmol, 90% dry powder) was added to a solution of **34** (110 mg, 0.119 mmol) in DMF (1.0 mL) at 0 °C. The reaction mixture was then warmed to room temperature over 30 min and stirred for 4 days. The reaction was quenched with sat. aq NH_4_Cl solution (2.0 mL) and the mixture was extracted with EtOAc (3 × 10 mL). The combined organic phases were dried over Na_2_SO_4_ and concentrated under reduced pressure. The residue was purified by reverse phase HPLC (Phenomenex Gemini C18, H_2_O/CH_3_CN gradient 35–95% MeCN, 0.1% TFA). The fractions were neutralized with sat. aq NaHCO_3_ solution and concentrated to afford the title compound (17 mg, 18%). LCMS (ESI) method 1: RT = 2.287 min, *m*/*z* = 813.9 (M + H). ^1^H NMR (400 MHz, CDCl_3_): δ 7.71 (d, *J* = 8.4 Hz, 1H), 7.42 (s, 1H), 7.31 (d, *J* = 8.8 Hz, 1H), 6.97 (s, 1H), 6.59 (s, 2H), 4.79 (dd, *J* = 12.8, 6.0 Hz, 1H), 4.61–4.53 (m, 1H), 4.24–4.13 (m, 3H), 4.08 (s, 3H), 4.01–3.89 (m, 7H), 3.70 (d, *J* = 10.0 Hz, 1H), 3.60–3.55 (m, 1H), 3.45–3.35 (m, 3H), 3.08 (t, *J* = 9.6 Hz, 1H), 2.37–2.31 (m, 4H), 2.30 (s, 6H), 2.27–2.10 (m, 3H), 1.66 (q, *J* = 11.6 Hz, 1H), 1.12 (d, *J* = 6.8 Hz, 3H).

#### (2^6^3^4^ *S*_*a*_,1^7^2^2^ *R*_*a*_,2^4^ *R*)-2^7^-Chloro-2^10^-(3-(4-chloro-3,5-dimethylphenoxy)­propyl)-1^4^,1^5^-dimethoxy-2^4^,3^1^,3^3^-trimethyl-2^1^-oxo-2^1^,2^2^,2^3^,2^4^-tetrahydro-1^1^ *H*,3^1^ *H*-5-oxa-2­(2,6)-pyrazino­[1,2-*a*]­indola-1­(7,1)-indola-3­(4,5)-pyrazolacyclooctaphane-1^2^-carboxylic Acid (**11**)

##### Step A: Ethyl 7-(5-((3-(Benzyloxy)­propoxy)­methyl)-1,3-dimethyl-1*H*-pyrazol-4-yl)-6-chloro-3-(3-(4-chloro-3,5-dimethylphenoxy)­propyl)-1*H*-indole-2-carboxylate

In a microwave vial, compound **26** (3.0 g, 6.0 mmol, 1.0 equiv), 4-bromo-1,3-dimethyl-1*H*-pyrazol-5-yl)­methanol (3.6 g, 9.0 mmol, 1.5 equiv), K_2_CO_3_ (2.5 g, 18.0 mmol, 3.0 equiv), and Pd­(PPh_3_)_4_ (480 mg, 0.06 mmol, 0.01 equiv) were combined and dissolved in dioxane (60 mL) and H_2_O (15 mL). The reaction was sparged with argon for 5 min and then the reaction was irradiated in the microwave at 140 °C for 30 min. The reaction was extracted with EtOAc, washed with H_2_O, dried over MgSO_4_, filtered, and concentrated. The crude residue was purified by flash column chromatography eluting with 0 to 50% EtOAc in DCM to afford the title compound (3.74 g, 90% yield). LCMS (ESI) method 2: RT = 1.251 min, *m*/*z* = 692.6 [M + H]^+^. ^1^H NMR (CDCl_3_, 400 MHz): δ (ppm) 8.78 (s, 1H, –NH), 7.61 (d, *J* = 8.8 Hz, 2H), 7.36–7.28 (m, 5H), 7.24 (d, *J* = 8.8 Hz, 2H), 6.65 (s, 2H), 4.44 (s, 2H), 4.42–4.33 (m, 2H), 4.28 (d, *J* = 12.0 Hz, 1H), 4.17 (d, *J* = 12.0 Hz, 1H), 3.99 (t, *J* = 6.0 Hz, 1H), 3.92 (s, 3H), 3.57 (d, *J* = 6.0 Hz, 1H), 3.55–3.40 (m, 6H), 2.36 (s, 6H), 2.17 (t, *J* = 6.4 Hz, 1H), 2.14 (s, 3H), 1.91 (t, *J* = 6.4 Hz, 1H), 1.86–1.82 (m, 2H), 1.40 (t, *J* = 7.2 Hz, 1H).

##### Step B: (*R*)-6-(5-((3-(Benzyloxy)­propoxy)­methyl)-1,3-dimethyl-1*H*-pyrazol-4-yl)-7-chloro-10-(3-(4-chloro-3,5-dimethylphenoxy)­propyl)-4-methyl-3,4-dihydropyrazino­[1,2-*a*]­indol-1­(2*H*)-one

The product from step A (4.15 g, 6.0 mmol, 1 equiv), compound **29** (2.41 g, 10.2 mmol, 1.7 equiv), and Cs_2_CO_3_ (5.85 g, 18.0 mmol, 3 equiv) were dissolved in MeCN (30 mL) and heated at 65 °C for 8 h. The reaction was cooled to RT and concentrated. The residue was dissolved in EtOAc and H_2_O, extracted with EtOAc, dried over MgSO_4_, filtered, and then concentrated. The crude product was dissolved in DCM (30 mL). Trifluoroacetic acid (4.6 mL, 60 mmol, 10 equiv) was added, and the reaction was stirred at 40 °C for 2 h. The reaction was concentrated, and the crude residue was dissolved in ethanol (20 mL). K_2_CO_3_ (2.48 g, 18 mmol, 3.0 equiv) was added, and the reaction was heated to 60 °C for 2 h. The solvent was removed and the reaction extracted with EtOAc, washed with brine, dried over MgSO_4_, filtered, and concentrated. The crude product was purified by flash column chromatography eluting with 0 to 70% EtOAc (with 5% of MeOH) in DCM to afford the white solid title compound (3.27 g, 77% yield over 3 steps). ^1^H NMR (MeOD, 400 MHz): δ (ppm) 7.71 (dd, *J* = 8.8, 2.4 Hz, 1H), 7.35–7.22 (m, 6H), 6.66 (s, 2H), 4.49–4.41 (m, 1H), 4.36 (s, 2H), 4.23 (d, *J* = 12.0 Hz, 1H), 4.14 (d, *J* = 12.0 Hz, 1H), 3.96 (t, *J* = 7.2 Hz, 2H), 3.89 (s, 3H), 3.75–3.68 (m, 1H), 3.59–3.55 (m, 1H), 3.50–3.47 (m, 2H), 3.42–3.37 (m, 2H), 3.22–3.14 (m, 2H), 2.31 (s, 6H), 2.20–2.13 (m, 2H), 2.13 (s, 3H), 1.75 (t, *J* = 6.4 Hz, 1H), 1.65 (t, *J* = 13.2 Hz, 1H), 0.97 (dd, *J* = 9.6, 6.8 Hz, 3H); LCMS (ESI) method 2: RT = 1.059 min, *m*/*z* = 703.6 [M + H]^+^.

##### Step C: Methyl (*R*)-7-(6-(5-((3-(Benzyloxy)­propoxy)­methyl)-1,3-dimethyl-1*H*-pyrazol-4-yl)-7-chloro-10-(3-(4-chloro-3,5-dimethylphenoxy)­propyl)-4-methyl-1-oxo-3,4-dihydropyrazino [1,2-*a*]­indol-2­(1*H*)-yl)-4,5-dimethoxy-1*H*-indole-2-carboxylate

The title compound (0.548 g, 59% yield) was prepared following general procedure A using the product from step B (0.703 g, 1.0 mmol, 1 equiv) and methyl 7-bromo-4,5-dimethoxy-1*H*-indole-2-carboxylate (0.90 g, 2.5 mmol, 2.5 equiv). The crude product was purified by flash column chromatography eluting with 0 to 80% EtOAc in DCM. LCMS (ESI) method 3: RT = 1.189 min, *m*/*z* = 936.6 [M + H]^+^.

##### Step D: Methyl (*R*)-7-(7-Chloro-10-(3-(4-chloro-3,5-dimethylphenoxy)­propyl)-6-(5-((3-hydroxypropoxy)­methyl)-1,3-dimethyl-1*H*-pyrazol-4-yl)-4-methyl-1-oxo-3,4-dihydropyrazino­[1,2-*a*]­indol-2­(1*H*)-yl)-4,5-dimethoxy-1*H*-indole-2-carboxylate

To a solution of the product from step C (281 mg, 0.3 mmol, 1 equiv) in a mixture of MeOH (6 mL) and THF (2 mL) was added Pd­(OH)_2_ (84 mg, 0.06 mmol, 0.2 equiv). The reaction mixture was sparged with balloon hydrogen for 5 min, stirred for 2 h at 38 °C. The reaction mixture was filtered through Celite and concentrated. The crude product was purified by flash column chromatography eluting with 0 to 50% EtOAc in DCM to afford the title compound (240 mg, 95% yield). LCMS (ESI) method 3: RT = 0.913, 0.938 min, *m*/*z* = 846.6 [M + H]^+^.

##### Step E: Methyl (*R*)-7-(6-(5-((3-Bromopropoxy)­methyl)-1,3-dimethyl-1*H*-pyrazol-4-yl)-7-chloro-10-(3-(4-chloro-3,5-dimethylphenoxy)­propyl)-4-methyl-1-oxo-3,4-dihydropyrazino [1,2-*a*]­indol-2­(1*H*)-yl)-4,5-dimethoxy-1*H*-indole-2-carboxylate

Bromine (18 μL, 0.354 mmol, 1.5 equiv) was slowly added to mixture of PPh_3_ (93 mg, 0.354 mmol, 1.5 equiv) and imidazole (29 mg, 0.425 mmol, 1.8 equiv) in DCM (3 mL) at 0 °C. After stirring 10 min, the product from step D (200 mg, 0.236 mmol, 1 equiv) in DCM (3 mL) was added to the reaction mixture dropwise for 5 min. The reaction mixture was stirred for 30 min at 0 °C, quenched with sat. NaHCO_3_ (10 mL), extracted with DCM (3 × 15 mL), dried over MgSO_4_, and concentrated. The crude product was purified by flash column chromatography eluting with 0 to 40% EtOAc in DCM to afford the title compound (154 mg, 74% yield). LCMS (ESI) method 3: RT = 1.133 min, *m*/*z* = 910.5 [M + H]^+^.

##### Step F: Methyl (2^6^3^4^ *S*_*a*_,1^7^2^2^ *R*_*a*_,24*R*)-2^7^-Chloro-2^10^-(3-(4-chloro-3,5-dimethylphenoxy) propyl)-1^4^,1^5^-dimethoxy-2^4^,3^1^,3^3^-trimethyl-2^1^-oxo-2^1^,2^2^,2^3^,2^4^-tetrahydro-1^1^ *H*,3^1^ *H*-5-oxa-2­(2,6)-pyrazino­[1,2-*a*]­indola-1­(7,1)-indola-3­(4,5)-pyrazolacyclooctaphane-1^2^-carboxylate

A mixture of the product from step E (158 mg, 0.174 mmol, 1 equiv), Cs_2_CO_3_ (170 mg, 0.521 mmol, 3 equiv) in DMF (3 mL) was heated at 45 °C for 7 h, diluted with EtOAc, washed with water, dried over MgSO_4_, and concentrated. The crude product was purified by flash column chromatography eluting with 0 to 15% EtOAc (premixed with 1% MeOH) in DCM to afford the white solid title compound (50 mg, 35% yield). LCMS (ESI) method 3: RT = 1.122 min, *m*/*z* = 828.6 [M + H]^+^.

##### Step G: (2^6^3^4^ *S*_*a*_,1^7^2^2^ *R*_*a*_,24*R*)-2^7^-Chloro-2^10^-(3-(4-chloro-3,5-dimethylphenoxy)­propyl)-1^4^,1^5^-dimethoxy-2^4^,3^1^,3^3^-trimethyl-2^1^-oxo-2^1^,2^2^,2^3^,2^4^-tetrahydro-1^1^ *H*,3^1^ *H*-5-oxa-2­(2,6)-pyrazino­[1,2-*a*]­indola-1­(7,1)-indola-3­(4,5)-pyrazolacyclooctaphane-1^2^-carboxylic Acid (**11**)

The title compound (22 mg, 56% yield) was prepared following general procedure F using the product from step F. The crude product was purified by Gilson reverse phase HPLC. ^1^H NMR (MeOD, 400 MHz): δ 7.75 (d, *J* = 8.4 Hz, 1H), 7.44 (s, 1H), 7.32 (d, *J* = 8.4 Hz, 1H), 7.05 (s, 1H), 6.57 (s, 2H), 4.73 (dd, *J* = 12.4, 5.6 Hz, 1H), 4.65–4.56 (m, 1H), 4.24 (d, *J* = 11.2 Hz, 2H), 4.04 (s, 3H), 4.02–3.89 (m, 4H), 3.93 (s, 3H), 3.87 (s, 3H), 3.72 (dd, *J* = 9.2, 4.0 Hz, 1H), 3.63 (d, *J* = 10.8 Hz, 1H), 3.58 (d, *J* = 10.0 Hz, 1H), 3.06 (t, *J* = 10.0 Hz, 1H), 2.31 (s, 3H), 2.27 (s, 6H), 2.22–2.10 (m, 3H), 2.05–1.98 (m, 1H), 1.66 (dd, *J* = 24.0, 10.8 Hz, 1H), 1.12 (*J* = 6.8 Hz, 3H); LCMS (ESI) method 3: RT = 0.981 min, *m*/*z* = 814.6 [M + H]^+^.

#### (2^6^3^4^ *S*_*a*_,1^7^2^2^ *R*_*a*_,24*R*)-2^7^-Chloro-2^10^-(3-(4-chloro-3,5-dimethylphenoxy)­propyl)-1^4^,1^5^-dimethoxy-2^4^,3^1^,3^5^-trimethyl-2^1^-oxo-2^1^,2^2^,2^3^,2^4^-tetrahydro-1^1^ *H*,3^1^ *H*-5-oxa-2­(2,6)-pyrazino­[1,2-*a*]­indola-1­(7,1)-indola-3­(4,3)-pyrazolacyclononaphane-1^2^-carboxylic Acid (**12**)

##### Step A: Ethyl 7-(3-((Benzyloxy)­methyl)-1,5-dimethyl-1*H*-pyrazol-4-yl)-6-chloro-3-(3-(4-chloro-3,5-dimethylphenoxy)­propyl)-1*H*-indole-2-carboxylate

In a microwave vial, compound **26** (3.5 g, 7.0 mmol, 1.0 equiv), 3-((benzyloxy)­methyl)-1,5-dimethyl-4-(4,4,5,5-tetramethyl-1,3,2-dioxaborolan-2-yl)-1*H*-pyrazole (3.6 g, 10.5 mmol, 1.5 equiv), K_2_CO_3_ (2.9 g, 21.0 mmol, 3.0 equiv), and Pd­(PPh_3_)_4_ (560 mg, 0.07 mmol, 0.07 equiv) were combined and dissolved in dioxane (60 mL) and H_2_O (15 mL). The reaction was sparged with argon for 5 min and then the reaction was irradiated in the microwave at 140 °C for 30 min. The reaction was extracted with EtOAc, washed with H_2_O, dried over MgSO_4_, filtered, and concentrated. The crude residue was purified by flash column chromatography eluting with 0 to 100% EtOAc in hexanes to afford the title compound (3.5 g, 79% yield).

##### Step B: Ethyl (*R*)-7-(3-((Benzyloxy)­methyl)-1,5-dimethyl-1*H*-pyrazol-4-yl)-1-(1-((*tert*-butoxycarbonyl)­amino)­propan-2-yl)-6-chloro-3-(3-(4-chloro-3,5-dimethylphenoxy)­propyl)-1*H*-indole-2-carboxylate

The product from step A (1.30 g, 2.05 mmol, 1.0 equiv) and *tert*-butyl (*S*)-5-methyl-1,2,3-oxathiazolidine-3-carboxylate 2,2-dioxide (778 mg, 3.28 mmol, 1.6 equiv) were dissolved in MeCN (18 mL). Cs_2_CO_3_ (1.33 g, 4.1 mmol, 2.0 equiv) was added, and the reaction was stirred at 80 °C for 24 h. The crude reaction was extracted with EtOAc, washed with H_2_O, dried over MgSO_4_, filtered, and concentrated. The crude residue was purified by flash column chromatography eluting with 0 to 100% EtOAc in hexanes to afford the title compound (1.62 g, quant. yield).

##### Step C: (*R*)-6-(3-((Benzyloxy)­methyl)-1,5-dimethyl-1*H*-pyrazol-4-yl)-7-chloro-10-(3-(4-chloro-3,5-dimethylphenoxy)­propyl)-4-methyl-3,4-dihydropyrazino­[1,2-*a*]­indol-1­(2*H*)-one

The product from step B (1.62 g, 2.05 mmol, 1.0 equiv) was dissolved in DCM (10 mL) and TFA (1.6 mL) was added. The reaction was allowed to stir for 2 h. The crude residue was taken up in MeOH (10 mL), and K_2_CO_3_ (5.65 g, 40.9 mmol, 20 equiv) was added. The reaction was stirred at room temperature for 60 h. The reaction mixture was extracted with DCM, washed with aq NH_4_Cl, dried over MgSO_4_, filtered, and concentrated. The crude residue was purified by flash column chromatography to afford the title compound (1.0 g, 76% yield).

##### StepD: Ethyl (*R*)-7-(6-(5-((Benzyloxy)­methyl)-1,3-dimethyl-1*H*-pyrazol-4-yl)-7-chloro-10-(3-(4-chloro-3,5-dimethylphenoxy)­propyl)-4-methyl-1-oxo-3,4-dihydropyrazino­[1,2-*a*]­indol-2­(1*H*)-yl)-4,5-dimethoxy-1*H*-indole-2-carboxylate

The title compound was prepared following general procedure A using the product from step C (780 mg 1.21 mmol) and methyl 7-iodo-4,5-dimethoxy-1*H*-indole-2-carboxylate (906 mg, 2.42 mmol). The crude residue was purified by reverse phase HPLC to afford the title compound (302 mg, 28% yield). LCMS (ESI) method 2: RT = 1.912, *m*/*z* = 891.9 (M + H). The undesired atropisomer (312 mg, 29% yield, LCMS (ESI) method 2: RT = 1.856 min, *m*/*z* = 891.9 (M + H)) was also isolated separately.

##### Step E: Ethyl (*R*)-7-(7-Chloro-10-(3-(4-chloro-3,5-dimethylphenoxy)­propyl)-6-(5-(hydroxymethyl)-1,3-dimethyl-1*H*-pyrazol-4-yl)-4-methyl-1-oxo-3,4-dihydropyrazino­[1,2-*a*]­indol-2­(1*H*)-yl)-4,5-dimethoxy-1*H*-indole-2-carboxylate

The title compound (216 mg, 79% yield) was prepared following general procedure C using the product from step D (302 mg, 0.34 mmol). LCMS (ESI) method 2: RT = 1.173 min, MS (ES) 801.9 (M + H).

##### Step F: Ethyl (*R*)-1-Allyl-7-(7-chloro-10-(3-(4-chloro-3,5-dimethylphenoxy)­propyl)-6-(5-(hydroxymethyl)-1,3-dimethyl-1*H*-pyrazol-4-yl)-4-methyl-1-oxo-3,4-dihydropyrazino­[1,2-*a*]­indol-2­(1*H*)-yl)-4,5-dimethoxy-1*H*-indole-2-carboxylate

In a reaction vessel, the product from step E (30 mg, 0.037 mmol) was dissolved in DMF (0.5 mL). Cs_2_CO_3_ (37.0 mg, 0.111 mmol) and allyl bromide (8.9 mg, 0.074 mmol) were added, and the reaction was heated to 60 °C for 16 h. Upon completion, the reaction was extracted with EtOAc, washed with H_2_O, dried over MgSO_4_, filtered, and concentrated. The crude residue was taken to the next step without further purification.

##### Step G: Ethyl (*R*)-1-Allyl-7-(6-(5-((allyloxy)­methyl)-1,3-dimethyl-1*H*-pyrazol-4-yl)-7-chloro-10-(3-(4-chloro-3,5-dimethylphenoxy)­propyl)-4-methyl-1-oxo-3,4-dihydropyrazino­[1,2-*a*]­indol-2­(1*H*)-yl)-4,5-dimethoxy-1*H*-indole-2-carboxylate

The product from step F (0.037 mmol) was dissolved in DMF (0.4 mL) and cooled to 0 °C. Sodium hydride (1.5 mg, 0.055 mmol, 90% pure) was added, followed by addition of allyl bromide (8.9 mg, 0.074 mmol). The reaction was warmed to RT and stirred until complete by LCMS. The reaction was extracted with EtOAc, washed with H_2_O, dried over Na_2_SO_4_, filtered, and concentrated. The crude residue was purified by flash column chromatography eluting with 0 to 100% EtOAc in hexanes to afford the title compound (14 mg, 43% yield over 2 steps). LCMS (ESI) method 2: RT = 2.015 min, 2.060 min *m*/*z* = 881.8 (M + H).

##### Step H: Ethyl (2^6^3^4^ *R*_*a*_,1^7^2^2^ *R*_*a*_,24*R*,*E*)-2^7^-Chloro-2^10^-(3-(4-chloro-3,5-dimethylphenoxy) propyl)-1^4^,1^5^-dimethoxy-2^4^,3^1^,3^3^-trimethyl-2^1^-oxo-2^1^,2^2^,2^3^,2^4^-tetrahydro-1^1^ *H*,3^1^ *H*-5-oxa-2­(2,6)-pyrazino­[1,2-*a*]­indola-1­(7,1)-indola-3­(4,5)-pyrazolacyclononaphan-7-ene-1^2^-carboxylate

The product from step G (8 mg, 9.4 μmol) was dissolved in DCM (3 mL) and sparged with argon for 5 min. Grubbs second generation catalyst (0.8 mg, 0.9 μmol) was added and the reaction was heated to 40 °C for 20 h. Upon completion, the reaction mixture was filtered through Celite and concentrated. The crude residue was purified by flash column chromatography eluting with 0 to 100% EtOAc in hexanes to afford the title compound (7.5 mg, 95% yield). LCMS (ESI) method 2: RT = 1.976 min, *m*/*z* = 853.8 (M + H).

##### Step I: (2^6^3^4^ *S*_*a*_,1^7^2^2^ *R*_*a*_,24*R*)-2^7^-Chloro-2^10^-(3-(4-chloro-3,5-dimethylphenoxy)­propyl)-1^4^,1^5^-dimethoxy-2^4^,3^1^,3^5^-trimethyl-2^1^-oxo-2^1^,2^2^,2^3^,2^4^-tetrahydro-1^1^ *H*,3^1^ *H*-5-oxa-2­(2,6)-pyrazino­[1,2-*a*]­indola-1­(7,1)-indola-3­(4,3)-pyrazolacyclononaphane-1^2^-carboxylic Acid (**12**)

The product from step H (14.0 mg, 0.016 mmol) was dissolved in 3:1 mixture of EtOH: THF and the resulting solution was flushed with argon. Pd/C (3.5 mg, 0.0033 mmol, 10 wt %) was added to the solution and flushed with H_2_ gas. Next an H_2_ balloon was affixed, and the reaction mixture was stirred at 45 °C for 14 h. Then the reaction mixture was filtered through Celite and concentrated. The crude reaction mixture was saponified following general procedure F and purified by reverse phase HPLC to provide the title compound (4 mg, 30% yield). LCMS (ESI) method 2: RT = 1.431 min, *m*/*z* = 827.8 (M + H). ^1^H NMR (MeOD, 400 MHz): δ 7.64 (d, *J* = 8.8 Hz, 1H), 7.24 (s, 1H), 7.16 (d, *J* = 8.8 Hz, 1H), 6.88 (s, 1H), 6.50 (s, 2H), 4.60 (dd, *J* = 12.8, 5.2 Hz, 1H), 4.54–4.39 (m, 1H), 4.21 (d, *J* = 10.4 Hz, 1H), 4.13 (quint, 6.0 Hz, 1H), 3.92 (s, 2H), 3.88 (m, 2H), 3.80 (s, 3H), 3.79 (s, 3H), 3.74–3.65 (m, 2H), 3.45–3.36 (m, 2H), 2.91 (s, 1H), 2.81–2.76 (m, 1H), 2.19 (s, 3H), 2.14 (s, 6H), 2.09–1.98 (m, 2H), 1.85–1.76 (m, 2H), 1.56–1.46 (m, 1H), 1.13 (d, *J* = 6.8 Hz, 3H), 0.83–0.71 (m, 2H), 0.41–0.29 (m, 1H).

#### (2^6^3^4^ *S*_*a*_,1^7^2^2^ *R*_*a*_,2^4^ *R*)-27-Chloro-210-(3-(4-chloro-3,5-dimethylphenoxy)­propyl)-1^4^,1^5^-dimethoxy-2^4^,3^1^-dimethyl-21-oxo-2^1^,2^2^,2^3^,2^4^-tetrahydro-1^1^ *H*,3^1^ *H*-5-oxa-2­(2,6)-pyrazino­[1,2-*a*]­indola-1­(7,1)-indola-3­(4,3)-pyrazolacyclooctaphane-12-carboxylic Acid (**13**)

##### Step A: Ethyl (*R*)-7-(6-(3-((3-(Benzyloxy)­propoxy)­methyl)-1-methyl-1*H*-pyrazol-4-yl)-7-chloro-10-(3-(4-chloro-3,5-dimethylphenoxy)­propyl)-4-methyl-1-oxo-3,4-dihydropyrazino [1,2-*a*]­indol-2­(1*H*)-yl)-4,5-dimethoxy-1*H*-indole-2-carboxylate (**39**)

Compound **39** was prepared following general procedure B using compound **38** (750 mg, 1.09 mmol, 1.0 equiv) and compound **31A** (430 mg, 1.3 mmol, 1.2 equiv) and heating to 100 °C for 2 h. The crude residue was purified by flash column chromatography eluting with 0 to 100% 95:5 EtOAc/MeOH in hexanes to afford the desired product (919 mg, 90% yield). LCMS (ESI) method 2: RT = 1.929 min, *m*/*z* = 936.0 (M + H).

##### Step B: Ethyl (*R*)-7-(7-Chloro-10-(3-(4-chloro-3,5-dimethylphenoxy)­propyl)-6-(3-((3-hydroxypropoxy)­methyl)-1-methyl-1*H*-pyrazol-4-yl)-4-methyl-1-oxo-3,4-dihydropyrazino [1,2-*a*]­indol-2­(1*H*)-yl)-4,5-dimethoxy-1*H*-indole-2-carboxylate (**40**)

Compound **40** (826 mg, quantitative yield) was prepared following general procedure C using compound **39** (919 mg, 1.1 mmol, 1.0 equiv). LCMS (ESI) method 2: RT = 1.528 min, 1.579 min, *m*/*z* = 846.0 (M + H).

##### Step C: Ethyl (*R*)-7-(7-Chloro-10-(3-(4-chloro-3,5-dimethylphenoxy)­propyl)-4-methyl-6-(1-methyl-3-((3-(tosyloxy)­propoxy)­methyl)-1*H*-pyrazol-4-yl)-1-oxo-3,4-dihydropyrazino­[1,2-*a*]­indol-2­(1*H*)-yl)-4,5-dimethoxy-1*H*-indole-2-carboxylate (**41**)

Compound **41** (783 mg, 87% yield) was prepared following general procedure D using compound **40** (825 mg, 0.97 mmol, 1.0 equiv). The crude residue was purified by flash column chromatography eluting with 0 to 90% EtOAc in hexanes. LCMS (ESI) method 2: RT = 1.843 min, *m*/*z* = 999.9 (M + H).

##### Step D: Ethyl (2^6^3^4^ *S*_*a*_,1^7^2^2^ *R*_*a*_,2^4^ *R*)-2^7^-Chloro-2^10^-(3-(4-chloro-3,5-dimethylphenoxy) propyl)-1^4^,1^5^-dimethoxy-2^4^,3^1^-dimethyl-2^1^-oxo-2^1^,2^2^,2^3^,2^4^-tetrahydro-1^1^ *H*,3^1^ *H*-5-oxa-2­(2,6)-pyrazino­[1,2-*a*]­indola-1­(7,1)-indola-3­(4,3)-pyrazolacyclooctaphane-1^2^-carboxylate (**42**)

Compound **42** (564 mg, 76% yield) was prepared following general procedure E using compound **41** (900 mg, 0.90 mmol, 1.0 equiv). The crude residue was purified by flash column chromatography eluting with 0 to 50% 95:5 EtOAc/MeOH in hexanes to afford the desired product. LCMS (ESI) method 2: RT = 1.843 min, *m*/*z* = 828.0 (M + H).

##### Step E: (2^6^3^4^ *S*_*a*_,1^7^2^2^ *R*_*a*_,2^4^ *R*)-2^7^-Chloro-2^10^-(3-(4-chloro-3,5-dimethylphenoxy)­propyl)-1^4^,1^5^-dimethoxy-2^4^,3^1^-dimethyl-2^1^-oxo-2^1^,2^2^,2^3^,2^4^-tetrahydro-1^1^ *H*,3^1^ *H*-5-oxa-2­(2,6)-pyrazino­[1,2-*a*]­indola-1­(7,1)-indola-3­(4,3)-pyrazolacyclooctaphane-1^2^-carboxylic Acid (**13**)

Compound **13** (490 mg, 90% yield) was prepared following general procedure F using compound **42** (564 mg, 0.68 mmol, 1.0 equiv). LCMS (ESI) method 2: RT = 1.522 min, *m*/*z* = 799.9 (M + H). ^1^H NMR (CDCl_3_, 400 MHz): δ 7.66 (d, *J* = 8.4 Hz, 1H), 7.59 (s, 1H), 7.34 (s, 1H), 7.27 (d, *J* = 9.6 Hz, 1H), 6.94 (s, 1H), 6.57 (s, 2H), 4.81 (dd, *J* = 12.8, 6.0 Hz, 1H), 4.56 (dt, *J* = 12.0, 4.4 Hz, 1H), 4.25–4.10 (m, 3H), 4.07 (s, 3H), 4.06 (s, 3H), 3.98–3.86 (m, 5H), 3.70 (d, *J* = 13.6 Hz, 1H), 3.58 (dd, *J* = 8.4, 4.4 Hz, 1H), 3.43–3.32 (m, 3H), 3.04 (t, *J* = 10.0 Hz, 1H), 2.27 (s, 6H), 2.23–2.03 (m, 3H), 1.63 (q, *J* = 12.4 Hz, 1H), 1.10 (d, *J* = 6.8 Hz, 3H).

#### (2^6^3^4^ *S*_*a*_,1^7^2^2^ *R*_*a*_,2^4^ *R*)-2^7^-Chloro-2^10^-(3-(4-chloro-3,5-dimethylphenoxy)­propyl)-1^5^,2^4^,3^1^-trimethyl-2^1^-oxo-2^1^,2^2^,2^3^,2^4^-tetrahydro-1^1^ *H*,3^1^ *H*-5-oxa-2­(2,6)-pyrazino­[1,2-*a*]­indola-1­(7,1)-indola-3­(4,3)-pyrazolacyclooctaphane-1^2^-carboxylic Acid (14)

##### Step A: Ethyl (*R*)-7-(6-(3-((3-(Benzyloxy)­propoxy)­methyl)-1-methyl-1*H*-pyrazol-4-yl)-7-chloro-10-(4-(4-chloro-3,5-dimethylphenyl)­butyl)-4-methyl-1-oxo-3,4-dihydropyrazino­[1,2-*a*]­indol-2­(1*H*)-yl)-5-methyl-1*H*-indole-2-carboxylate

The title compound (220 mg, 85% yield) was prepared following general procedure A using compound **38** (286 mg, 0.870 mmol) and ethyl 7-iodo-5-methyl-1*H*-indole-2-carboxylate (200 mg, 0.29 mmol). LCMS (ESI) method 2: RT = 1.264 min, *m*/*z* = 890.5 (M + H).

##### Step B: Ethyl (*R*)-7-(7-Chloro-10-(3-(4-chloro-3,5-dimethylphenoxy)­propyl)-6-(3-((3-hydroxypropoxy)­methyl)-1-methyl-1*H*-pyrazol-4-yl)-4-methyl-1-oxo-3,4-dihydropyrazino [1,2-*a*]­indol-2­(1*H*)-yl)-5-methyl-1*H*-indole-2-carboxylate

The title compound (100 mg, 48% yield) was prepared following general procedure C using the product from step A (220 mg, 0.26 mmol). LCMS (ESI) method 2: RT = 1.038 min, *m*/*z* = 800.5 (M + H).

##### Step C: Ethyl (*R*)-7-(6-(3-((3-Bromopropoxy)­methyl)-1-methyl-1*H*-pyrazol-4-yl)-7-chloro-10-(3-(4-chloro-3,5-dimethylphenoxy)­propyl)-4-methyl-1-oxo-3,4-dihydropyrazino­[1,2-*a*]­indol-2­(1*H*)-yl)-5-methyl-1*H*-indole-2-carboxylate

The product from step B (100 mg, 0.125 mmol) and triphenylphosphine (52.4 mg, 0.200 mmol) were dissolved in DCM (5 mL) and the reaction was cooled to 0 °C. CBr_4_ (62 mg, 0.187 mmol, 1.5 equiv) was added, and the reaction was stirred at 0 °C for 1 h, followed by 2 h at RT. The reaction was concentrated, and the crude residue was purified by flash column chromatography eluting with 0 to 100% EtOAc in hexanes to afford the title compound (50 mg, 46% yield). LCMS (ESI) method 2: RT = 1.984 min, *m*/*z* = 861.7 (M + H).

##### Step D: Ethyl (2^6^3^4^ *S*_*a*_,1^7^2^2^ *R*_*a*_,2^4^ *R*)-2^7^-Chloro-2^10^-(3-(4-chloro-3,5-dimethylphenoxy) propyl)-1^5^,2^4^,3^1^-trimethyl-2^1^-oxo-2^1^,2^2^,2^3^,2^4^-tetrahydro-1^1^ *H*,3^1^ *H*-5-oxa-2­(2,6)-pyrazino [1,2-*a*]­indola-1­(7,1)-indola-3­(4,3)-pyrazolacyclooctaphane-1^2^-carboxylate

The title compound (45.0 mg, 99% yield) was prepared following general procedure E using Cs_2_CO_3_ (56.6 mg, 0.17 mmol) and the product from step C (50.0 mg, 0.058 mmol). LCMS (ESI) method 2: RT = 1.940 min, *m*/*z* = 781.8 (M + H).

##### Step E: (2^6^3^4^ *S*_*a*_,1^7^2^2^ *R*_*a*_,2^4^ *R*)-2^7^-Chloro-2^10^-(3-(4-chloro-3,5-dimethylphenoxy)­propyl)-1^5^,2^4^,3^1^-trimethyl-2^1^-oxo-2^1^,2^2^,2^3^,2^4^-tetrahydro-1^1^ *H*,3^1^ *H*-5-oxa-2­(2,6)-pyrazino­[1,2-*a*]­indola-1­(7,1)-indola-3­(4,3)-pyrazolacyclooctaphane-1^2^-carboxylic Acid (**14**)

The title compound (20.0 mg, 46% yield) was prepared following general procedure F using the product from step D (45.0 mg, 0.057 mmol). LCMS (ESI) method 1: RT = 2.367 min, *m*/*z* = 753.9 (M + H). ^1^H NMR (MeOD, 400 MHz): δ 7.90 (s, 1H), 7.73 (d, *J* = 8.4 Hz, 1H), 7.47 (s, 1H), 7.28 (d, *J* = 8.4 Hz, 1H), 7.22 (s, 1H), 7.03 (s, 1H), 6.59 (s, 2H), 4.78 (dd, *J* = 12.4, 5.6 Hz, 1H), 4.70 (dt, *J* = 12.8, 6.0 Hz, 1H), 4.27–4.17 (m, 2H), 4.15–4.08 (m, 2H), 4.04 (s, 2H), 4.02–3.99 (m, 1H), 3.96–3.85 (m, 2H), 3.69 (d, *J* = 10.0 Hz, 1H), 3.60–3.52 (m, 2H), 3.39–3.34 (m, 1H), 3.10 (t, *J* = 10.0 Hz, 1H), 2.45 (s, 3H), 2.27 (s, 6H), 2.19–2.11 (m, 3H), 1.62 (q, *J* = 12.0 Hz, 1H), 1.14 (d, *J* = 6.8 Hz, 3H).

#### Methyl (2^6^3^4^ *S*_*a*_,1^7^2^2^ *R*_*a*_,2^4^ *R*)-2^7^-Chloro-2^10^-(3-(4-chloro-3,5-dimethylphenoxy) propyl)-1^5^-methoxy-2^4^,3^1^-dimethyl-2^1^-oxo-2^1^,2^2^,2^3^,2^4^-tetrahydro-1^1^ *H*,3^1^ *H*-5-oxa-2­(2,6)-pyrazino­[1,2-*a*]­indola-1­(7,1)-indola-3­(4,3)-pyrazolacyclooctaphane-1^2^-carboxylate (**15**)

##### Step A: Methyl (*R*)-7-(6-(3-((3-(Benzyloxy)­propoxy)­methyl)-1-methyl-1*H*-pyrazol-4-yl)-7-chloro-10-(3-(4-chloro-3,5-dimethylphenoxy)­propyl)-4-methyl-1-oxo-3,4-dihydropyrazino [1,2-*a*]­indol-2­(1*H*)-yl)-5-methoxy-1*H*-indole-2-carboxylate

The title compound (160 mg, 82% yield) was prepared following general procedure A using compound **38** (150 mg, 0.22 mmol) and methyl 7-iodo-5-methoxy-1*H*-indole-2-carboxylate (216 mg, 0.65 mmol). LCMS (ESI) method 2: RT = 1.861 min, *m*/*z* = 892.1 (M + H).

##### Step B: Methyl (*R*)-7-(7-Chloro-10-(3-(4-chloro-3,5-dimethylphenoxy)­propyl)-6-(3-((3-hydroxypropoxy)­methyl)-1-methyl-1*H*-pyrazol-4-yl)-4-methyl-1-oxo-3,4-dihydropyrazino [1,2-*a*]­indol-2­(1*H*)-yl)-5-methoxy-1*H*-indole-2-carboxylate

The title compound (120 mg, 83% yield) was prepared following general procedure C using the product from step A (160 mg, 0.18 mmol) LCMS (ESI) method 2: RT = 0.924 min, *m*/*z* = 802.6 (M + H).

##### Step C: Methyl (*R*)-7-(6-(3-((3-Bromopropoxy)­methyl)-1-methyl-1*H*-pyrazol-4-yl)-7-chloro-10-(3-(4-chloro-3,5-dimethylphenoxy)­propyl)-4-methyl-1-oxo-3,4-dihydropyrazino­[1,2-*a*]­indol-2­(1*H*)-yl)-5-methoxy-1*H*-indole-2-carboxylate

The product from step B (120 mg, 0.15 mmol) and triphenylphosphine (86 mg, 0.33 mmol) were dissolved in DCM (10 mL) and the reaction was cooled to 0 °C. CBr_4_ (109 mg, 0.33 mmol, 1.5 equiv) was added, and the reaction was stirred at 0 °C for 1 h, followed by 2 h at room temperature. The reaction was concentrated, and the crude residue was purified by flash column chromatography eluting with 0 to 100% EtOAc in hexanes to afford the title compound (100 mg, 77% yield). LCMS (ESI) method 2: RT = 1.092 min, *m*/*z* = 864.6 (M + H).

##### Step D: Methyl (2^6^3^4^ *S*_*a*_,1^7^2^2^ *R*_*a*_,2^4^ *R*)-2^7^-Chloro-2^10^-(3-(4-chloro-3,5-dimethylphenoxy) propyl)-1^5^-methoxy-2^4^,3^1^-dimethyl-2^1^-oxo-2^1^,2^2^,2^3^,2^4^-tetrahydro-1^1^ *H*,3^1^ *H*-5-oxa-2­(2,6)-pyrazino­[1,2-*a*]­indola-1­(7,1)-indola-3­(4,3)-pyrazolacyclooctaphane-1^2^-carboxylate

The title compound (90.0 mg, 99% yield) was prepared following general procedure E using the product from step C (100 mg, 0.12 mmol). LCMS (ESI) method 2: RT = 1.064 min, *m*/*z* = 784.5 (M + H).

##### Step E: (2^6^3^4^ *S*_*a*_,1^7^2^2^ *R*_*a*_,2^4^ *R*)-2^7^-Chloro-2^10^-(3-(4-chloro-3,5-dimethylphenoxy)­propyl)-1^5^-methoxy-2^4^,3^1^-dimethyl-2^1^-oxo-2^1^,2^2^,2^3^,2^4^-tetrahydro-1^1^ *H*,3^1^ *H*-5-oxa-2­(2,6)-pyrazino­[1,2-*a*]­indola-1­(7,1)-indola-3­(4,3)-pyrazolacyclooctaphane-1^2^-carboxylic Acid (**15**)

The title compound (18.0 mg, 20% yield) was prepared following general procedure F using the product from step D (90 mg, 0.12 mmol) and LiOH (8.2 mg, 344 μmol). LCMS (ESI) method 2: RT = 0.926 min, *m*/*z* = 770.6 (M + H)^+^. ^1^H NMR (MeOD, 400 MHz): δ 7.91 (s, 1H), 7.74 (d, *J* = 8.4 Hz, 1H), 7.32 (s, 1H), 7.29 (d, *J* = 8.4 Hz, 1H), 7.20 (d, *J* = 2.4 Hz, 1H), 6.91 (d, *J* = 2.4 Hz, 1H), 6.59 (s, 2H), 4.78 (dd, *J* = 12.8, 6.0 Hz, 1H), 4.65 (s, 1H), 4.25–4.15 (m, 3H), 4.04 (s, 3H), 3.94–3.85 (m, 5H), 3.70 (d, *J* = 10.0 Hz, 1H), 3.61–3.54 (m, 2H), 3.40–3.35 (m, 2H), 3.09 (t, *J* = 10.4 Hz, 1H), 2.27 (s, 6H), 2.19–2.08 (m, 3H), 1.63 (q, *J* = 12.0 Hz, 1H), 1.14 (d, *J* = 6.8 Hz, 3H).

#### (2^6^3^4^ *S*_*a*_,1^7^2^2^ *R*_*a*_,2^4^ *R*)-2^7^-Chloro-2^10^-(3-(4-chloro-3,5-dimethylphenoxy)­propyl)-1^4^-methoxy-2^4^,3^1^-dimethyl-2^1^-oxo-2^1^,2^2^,2^3^,2^4^-tetrahydro-1^1^ *H*,3^1^ *H*-5-oxa-2­(2,6)-pyrazino­[1,2-*a*]­indola-1­(7,1)-indola-3­(4,3)-pyrazolacyclooctaphane-1^2^-carboxylic Acid (**16**)

##### Step A: Ethyl (*R*)-7-(6-(3-((3-(Benzyloxy)­propoxy)­methyl)-1-methyl-1*H*-pyrazol-4-yl)-7-chloro-10-(3-(4-chloro-3,5-dimethylphenoxy)­propyl)-4-methyl-1-oxo-3,4-dihydropyrazino [1,2-*a*]­indol-2­(1*H*)-yl)-4-methoxy-1*H*-indole-2-carboxylate

The title compound (450 mg, 68% yield) was prepared following general procedure A using compound **38** (500 mg, 0.73 mmol) and ethyl 7-iodo-4-methoxy-1*H*-indole-2-carboxylate (1.00 g, 2.90 mmol). LCMS (ESI) method 2: RT = 1.970 min, *m*/*z* = 906.1 (M + H).

##### Step B: Ethyl (*R*)-7-(7-Chloro-10-(3-(4-chloro-3,5-dimethylphenoxy)­propyl)-6-(3-((3-hydroxypropoxy)­methyl)-1-methyl-1*H*-pyrazol-4-yl)-4-methyl-1-oxo-3,4-dihydropyrazino [1,2-*a*]­indol-2­(1*H*)-yl)-4-methoxy-1*H*-indole-2-carboxylate

The title compound (300 mg, 74% yield) was prepared following general procedure C using the product from step A (450 mg, 0.49 mmol). LCMS (ESI) method B: RT = 1.560 min, *m*/*z* = 816.1 (M + H).

##### Step C: Ethyl (*R*)-7-(6-(3-((3-Bromopropoxy)­methyl)-1-methyl-1*H*-pyrazol-4-yl)-7-chloro-10-(3-(4-chloro-3,5-dimethylphenoxy)­propyl)-4-methyl-1-oxo-3,4-dihydropyrazino­[1,2-*a*]­indol-2­(1*H*)-yl)-4-methoxy-1*H*-indole-2-carboxylate

The product from step B (300 mg, 0.37 mmol) and triphenylphosphine (145 mg, 0.55 mmol) was dissolved in DCM (10 mL) and the reaction was cooled to 0 °C. CBr_4_ (183 mg, 0.55 mmol, 1.5 equiv) was added, and the reaction was allowed to stir at 0 °C for 1 h, followed by 2 h at room temperature. The reaction was concentrated, and the crude residue was purified by flash column chromatography eluting with 0 to 100% EtOAc in hexanes to afford the title compound (170 mg, 53% yield). LCMS (ESI) method 2: RT = 1.902 min, *m*/*z* = 878.1 (M + H).

##### Step D: Ethyl (2^6^3^4^ *S*_*a*_,1^7^2^2^ *R*_*a*_,2^4^ *R*)-2^7^-Chloro-2^10^-(3-(4-chloro-3,5-dimethylphenoxy) propyl)-1^4^-methoxy-2^4^,3^1^-dimethyl-2^1^-oxo-2^1^,2^2^,2^3^,2^4^-tetrahydro-1^1^ *H*,3^1^ *H*-5-oxa-2­(2,6)-pyrazino­[1,2-*a*]­indola-1­(7,1)-indola-3­(4,3)-pyrazolacyclooctaphane-1^2^-carboxylate

The title compound (150 mg, 97% yield) was prepared following general procedure E using the product from step C (170 mg, 0.19 mmol). The crude reaction product was carried forward to step E without purification. LCMS (ESI) method 2: RT = 1.933 min, *m*/*z* = 798.1 (M + H).

##### Step E: (2^6^3^4^ *S*_*a*_,1^7^2^2^ *R*_*a*_,2^4^ *R*)-2^7^-Chloro-2^10^-(3-(4-chloro-3,5-dimethylphenoxy)­propyl)-1^4^-methoxy-2^4^,3^1^-dimethyl-2^1^-oxo-2^1^,2^2^,2^3^,2^4^-tetrahydro-1^1^ *H*,3^1^ *H*-5-oxa-2­(2,6)-pyrazino­[1,2-*a*]­indola-1­(7,1)-indola-3­(4,3)-pyrazolacyclooctaphane-1^2^-carboxylic Acid (**16**)

The title compound (35 mg, 24% yield) was prepared following general procedure F using the product from step D (150 mg, 0.19 mmol). LCMS (ESI) method 1: RT = 2.281 min, *m*/*z* = 770.1 (M + H). ^1^H NMR (MeOD, 400 MHz): δ 7.91 (s, 1H), 7.73 (d, *J* = 8.4 Hz, 1H), 7.41 (s, 1H), 7.28 (d, *J* = 8.4 Hz, 1H), 7.13 (d, *J* = 8.4 Hz, 1H), 6.65 (d, *J* = 8.4 Hz, 1H), 6.58 (s, 2H), 4.77 (dd, *J* = 12.4, 5.6 Hz, 1H), 4.67 (s, 1H), 4.30–4.12 (m, 3H), 4.04 (s, 3H), 3.98 (s, 3H), 3.95–3.86 (m, 2H), 3.69 (d, *J* = 10.0 Hz, 1H), 3.61–3.50 (m, 2H), 3.39–3.35 (m, 2H), 3.09 (t, *J* = 10.0 Hz, 1H), 2.27 (s, 6H), 2.19–2.10 (m, 3H), 1.65 (q, *J* = 13.2 Hz, 1H), 1.13 (d, *J* = 6.4 Hz, 3H).

#### (2^6^3^4^ *S*_*a*_,1^7^2^2^ *R*_*a*_,2^4^ *R*)-2^7^-Chloro-2^10^-(3-(4-chloro-3,5-dimethylphenoxy)­propyl)-1^5^-methoxy-1^4^,2^4^,3^1^-trimethyl-2^1^-oxo-2^1^,2^2^,2^3^,2^4^-tetrahydro-1^1^ *H*,3^1^ *H*-5-oxa-2­(2,6)-pyrazino­[1,2-*a*]­indola-1­(7,1)-indola-3­(4,3)-pyrazolacyclooctaphane-1^2^-carboxylic Acid (**17**)

##### Step A: Ethyl (*R*)-7-(6-(3-((3-(Benzyloxy)­propoxy)­methyl)-1-methyl-1*H*-pyrazol-4-yl)-7-chloro-10-(3-(4-chloro-3,5-dimethylphenoxy)­propyl)-4-methyl-1-oxo-3,4-dihydropyrazino [1,2-*a*]­indol-2­(1*H*)-yl)-5-methoxy-4-methyl-1*H*-indole-2-carboxylate

The title compound (311 mg, 80% yield) was prepared following general procedure B using compound **38** (300 mg, 0.43 mmol, 1.0 equiv) and ethyl 7-bromo-5-methoxy-4-methyl-1*H*-indole-2-carboxylate (136 mg, 0.46 mmol, 1.05 equiv). LCMS (ESI) method 2: RT = 1.762 min, *m*/*z* = 906.3 (M + H)^+^.

##### Step B: Ethyl (*R*)-7-(7-Chloro-10-(3-(4-chloro-3,5-dimethylphenoxy)­propyl)-6-(3-((3-hydroxypropoxy)­methyl)-1-methyl-1*H*-pyrazol-4-yl)-4-methyl-1-oxo-3,4-dihydropyrazino [1,2-*a*]­indol-2­(1*H*)-yl)-5-methoxy-4-methyl-1*H*-indole-2-carboxylate

The title compound (280 mg, quant. yield) was prepared following general procedure C using the product from step A (310 mg, 0.34 mmol, 1.0 equiv). LCMS (ESI) method 2: RT = 1.479 min, 1.528 min (mixture of rotamers), *m*/*z* = 816.3 (M + H)^+^.

##### Step C. Ethyl (*R*)-7-(7-Chloro-10-(3-(4-chloro-3,5-dimethylphenoxy)­propyl)-4-methyl-6-(1-methyl-3-((3-(tosyloxy)­propoxy)­methyl)-1*H*-pyrazol-4-yl)-1-oxo-3,4-dihydropyrazino­[1,2-*a*]­indol-2­(1*H*)-yl)-5-methoxy-4-methyl-1*H*-indole-2-carboxylate

The product from step B (280 mg, 0.33 mmol, 1.0 equiv) was added to a reaction vessel and dissolved in DCM (10 mL). Tosyl chloride (250 mg, 1.32 mmol, 4 equiv), TEA (0.290 mL, 2.01 mmol, 6 equiv), and DMAP (40 mg, 0.33 mmol, 1 equiv) were added, and the reaction as allowed to stir at 30 °C for 24 h. The reaction mixture extracted with DCM, washed with water, 1 M HCl, brine, dried over MgSO_4_, filtered, and concentrated. The crude reaction mixture was purified by flash column chromatography eluting with 0 to 100% EtOAc in hexanes to afford the desired product (255 mg, 68% yield). LCMS method 2: RT = 1.666 min, *m*/*z* = 970.1 (M + H)^+^.

##### Step D: Ethyl (2^6^3^4^ *S*_*a*_,1^7^2^2^ *R*_*a*_,2^4^ *R*)-2^7^-Chloro-2^10^-(3-(4-chloro-3,5-dimethylphenoxy) propyl)-1^5^-methoxy-1^4^,2^4^,3^1^-trimethyl-2^1^-oxo-2^1^,2^2^,2^3^,2^4^-tetrahydro-1^1^ *H*,3^1^ *H*-5-oxa-2­(2,6)-pyrazino [1,2-*a*]­indola-1­(7,1)-indola-3­(4,3)-pyrazolacyclooctaphane-1^2^-carboxylate

The title compound (138 mg, 75% yield) was prepared following general procedure E using the product from step C (225 mg, 0.23 mmol, 1.0 equiv). The crude residue was purified by flash column chromatography eluting with 0 to 80% EtOAc in hexanes. LCMS (ESI) method 2: RT = 1.768 min, *m*/*z* = 798.2 (M + H)^+^.

##### Step E: (2^6^3^4^ *S*_*a*_,1^7^2^2^ *R*_*a*_,2^4^ *R*)-2^7^-Chloro-2^10^-(3-(4-chloro-3,5-dimethylphenoxy)­propyl)-1^5^-methoxy-1^4^,2^4^,3^1^-trimethyl-2^1^-oxo-2^1^,2^2^,2^3^,2^4^-tetrahydro-1^1^ *H*,3^1^ *H*-5-oxa-2­(2,6)-pyrazino­[1,2-*a*]­indola-1­(7,1)-indola-3­(4,3)-pyrazolacyclooctaphane-1_2_-carboxylic Acid (**17**)

The title compound (60 mg, 48% yield) was prepared following general procedure F using the product form step D (128 mg, 0.16 mmol, 1.0 equiv). LCMS (ESI) method 1: RT = 2.327 min, *m*/*z* = 784.2 (M + H)^+^. ^1^H NMR (MeOD, 400 MHz): δ 7.81 (s, 1H), 7.63 (d, *J* = 8.4 Hz, 1H), 7.18 (d, *J* = 8.4 Hz, 1H), 7.09 (s, 1H), 6.82 (s, 1H), 6.62 (s, 2H), 4.72–4.59 (m, 3H), 4.15–4.07 (m, 2H), 4.05 (d, *J* = 10.4 Hz, 1H), 3.96 (s, 3H), 3.87–3.80 (m, 2H), 3.79 (s, 3H), 3.61 (d, *J* = 10.4 Hz, 1H), 3.49 (d, *J* = 12.4 Hz, 2H), 3.30–3.26 (m, 1H), 3.03 (t, *J* = 10.4 Hz, 1H), 2.32 (s, 3H), 2.19 (s, 6H), 2.11–2.01 (m, 3H), 1.54 (quart, *J* = 12.4 Hz, 1H), 1.06 (d, *J* = 6.8 Hz, 3H).

#### (2^6^3^4^ *S*_*a*_,1^7^2^2^ *R*_*a*_,2^4^ *R*)-2^7^-Chloro-2^10^-(3-(4-chloro-3,5-dimethylphenoxy)­propyl)-1^4^-methoxy-1^5^,2^4^,3^1^-trimethyl-2^1^-oxo-2^1^,2^2^,2^3^,2^4^-tetrahydro-1^1^ *H*,3^1^ *H*-5-oxa-2­(2,6)-pyrazino­[1,2-*a*] Indola-1­(7,1)-indola-3­(4,3)-pyrazolacyclooctaphane-1^2^-carboxylic Acid (**18**)

##### Step A: Ethyl (*R*)-7-(6-(3-((3-(Benzyloxy)­propoxy)­methyl)-1-methyl-1*H*-pyrazol-4-yl)-7-chloro-10-(3-(4-chloro-3,5-dimethylphenoxy)­propyl)-4-methyl-1-oxo-3,4-dihydropyrazino [1,2-*a*]­indol-2­(1*H*)-yl)-4-methoxy-5-methyl-1*H*-indole-2-carboxylate

The title compound (250 mg, 63% yield) was prepared following general procedure B using compound **38** (300 mg, 0.44 mmol) and ethyl 7-iodo-4-methoxy-5-methyl-1*H*-indole-2-carboxylate (625 mg, 1.74 mmol). LCMS (ESI) method 2: RT = 2.308 min, *m*/*z* = 920.1 (M + H).

##### Step B: Ethyl (*R*)-7-(7-Chloro-10-(3-(4-chloro-3,5-dimethylphenoxy)­propyl)-6-(3-((3-hydroxypropoxy)­methyl)-1-methyl-1*H*-pyrazol-4-yl)-4-methyl-1-oxo-3,4-dihydropyrazino [1,2-*a*]­indol-2­(1*H*)-yl)-4-methoxy-5-methyl-1*H*-indole-2-carboxylate

The title compound (200 mg, 89% yield) was prepared following general procedure C using the product from step A (250 mg, 0.27 mmol) and Pd/C (10 wt %, 29.0 mg, 0.027 mmol). LCMS (ESI) method 2: RT = 1.523 min, *m*/*z* = 830.1 9 (M + H).

##### Step C: Ethyl (*R*)-7-(6-(3-((3-Bromopropoxy)­methyl)-1-methyl-1*H*-pyrazol-4-yl)-7-chloro-10-(3-(4-chloro-3,5-dimethylphenoxy)­propyl)-4-methyl-1-oxo-3,4-dihydropyrazino­[1,2-*a*]­indol-2­(1*H*)-yl)-4-methoxy-5-methyl-1*H*-indole-2-carboxylate

The product from step B (200 mg, 0.241 mmol) and triphenylphosphine (126 mg, 481 μmol) were dissolved in DCM (10 mL) and cooled to 0 °C. CBr_4_ (160 mg, 0.48 mmol, 1.5 equiv) was added, and the reaction was allowed to stir at 0 °C for 1 h, followed by 2 h at room temperature. The reaction was concentrated, and the crude residue was purified by flash column chromatography eluting with 0 to 100% EtOAc in hexanes to afford the title compound (85 mg, 40% yield). LCMS (ESI) method 2: RT = 1.942 min, *m*/*z* = 892.0 (M + H).

##### Step D: Ethyl (2^6^3^4^ *S*_*a*_,1^7^2^2^ *R*_*a*_,2^4^ *R*)-2^7^-Chloro-2^10^-(3-(4-chloro-3,5-dimethylphenoxy) propyl)-1^4^-methoxy-1^5^,2^4^,3^1^-trimethyl-2^1^-oxo-2^1^,2^2^,2^3^,2^4^-tetrahydro-1^1^ *H*,3^1^ *H*-5-oxa-2­(2,6)-pyrazino­[1,2-*a*]­indola-1­(7,1)-indola-3­(4,3)-pyrazolacyclooctaphane-1^2^-carboxylate

The title compound (75 mg, 97% yield) was prepared following general procedure F using the product from step C (85.0 mg, 0.095 mmol) and Cs_2_CO_3_ (93.0 mg, 0.290 mmol). The crude reaction product was carried to step E without purification. LCMS (ESI) method 2: RT = 1.948 min, *m*/*z* = 812.2 (M + H).

##### Step E: (2^6^3^4^ *S*_*a*_,1^7^2^2^ *R*_*a*_,2^4^ *R*)-2^7^-Chloro-2^10^-(3-(4-chloro-3,5-dimethylphenoxy)­propyl)-1^4^-methoxy-1^5^,2^4^,3^1^-trimethyl-2^1^-oxo-2^1^,2^2^,2^3^,2^4^-tetrahydro-1^1^ *H*,3^1^ *H*-5-oxa-2­(2,6)-pyrazino [1,2-*a*]­indola-1­(7,1)-indola-3­(4,3)-pyrazolacyclooctaphane-1^2^-carboxylic Acid (**18**)

The title compound (36.5 mg, 50% yield) was prepared following general procedure F using the product from step D (75.0 mg, 0.092 mmol) and LiOH (6.6 mg, 0.28 mmol). Following workup, the reaction was purified by reverse phase HPLC eluting with H_2_O/MeCN with 0.1% TFA additive. LCMS (ESI) method 2: RT = 0.966 min, *m*/*z* = 784.6 (M + H). ^1^H NMR (MeOD, 400 MHz): δ 7.81 (s, 1H), 7.63 (d, *J* = 8.4 Hz, 1H), 7.37 (s, 1H), 7.19 (d, *J* = 8.4 Hz, 1H), 6.98 (s, 1H), 6.48 (s, 2H), 4.66 (dd, *J* = 12.0, 5.6 Hz, 1H) 4.54–4.42 (m, 1H), 4.20–4.00 (m, 3H), 3.95 (s, 3H), 3.90 (s, 3H), 3.87–3.75 (m, 2H), 3.59 (d, *J* = 10.0 Hz, 1H), 3.51–3.42 (m, 2H), 3.31–3.24 (m, 2H), 2.98 (t, *J* = 10.0 Hz, 1H), 2.25 (s, 3H), 2.16 (s, 6H), 2.10–2.00 (m, 3H), 1.54 (q, *J* = 12.0 Hz, 1H), 1.04 (d, *J* = 6.8 Hz, 3H).

#### (2^6^3^4^ *S*_*a*_,1^7^2^2^ *R*_*a*_,2^4^ *R*)-2^7^-Chloro-2^10^-(3-(4-chloro-3,5-dimethylphenoxy)­propyl)-1^4^-methoxy-1^5^-(2-methoxyethoxy)-2^4^,3^1^-dimethyl-2^1^-oxo-2^1^,2^2^,2^3^,2^4^-tetrahydro-1^1^ *H*,3^1^ *H*-5-oxa-2­(2,6)-pyrazino­[1,2-*a*]­indola-1­(7,1)-indola-3­(4,3)-pyrazolacyclooctaphane-1^2^-carboxylic Acid (**19**)

##### Step A: Ethyl (2^6^3^4^ *S*_*a*_,1^7^2^2^ *R*_*a*_,2^4^ *R*)-2^7^-Chloro-2^10^-(3-(4-chloro-3,5-dimethylphenoxy) propyl)-1^4^-methoxy-1^5^-(2-methoxyethoxy)-2^4^,3^1^-dimethyl-2^1^-oxo-2^1^,2^2^,2^3^,2^4^-tetrahydro-1^1^ *H*,3^1^ *H*-5-oxa-2­(2,6)-pyrazino­[1,2-*a*]­indola-1­(7,1)-indola-3­(4,3)-pyrazolacyclooctaphane-1^2^-carboxylate

In a reaction vessel, compound **48** (20 mg, 0.025 mmol, 1.0 equiv) was dissolved in DMF (0.5 mL). 2-Bromoethyl methyl ether (10 mg, 0.072, 3.0 equiv) and Cs_2_CO_3_ (38 mg, 0.12 mmol, 5.0 equiv) were added, and the reaction was heated to 80 °C for 3 h. The reaction was extracted with EtOAc, washed with H_2_O, washed with brine, dried over MgSO_4_, filtered, and concentrated. The crude residue was purified by flash column chromatography eluting with 0 to 100% EtOAc in hexanes to afford the title compound (14 mg, 65% yield). LCMS (ESI) method 2: RT = 1.816 min, *m*/*z* = 871.9 (M + H).

##### Step B: (2^6^3^4^ *S*_*a*_,1^7^2^2^ *R*_*a*_,2^4^ *R*)-2^7^-Chloro-2^10^-(3-(4-chloro-3,5-dimethylphenoxy)­propyl)-1^4^-methoxy-1^5^-(2-methoxyethoxy)-2^4^,3^1^-dimethyl-2^1^-oxo-2^1^,2^2^,2^3^,2^4^-tetrahydro-1^1^ *H*,3^1^ *H*-5-oxa-2­(2,6)-pyrazino­[1,2-*a*]­indola-1­(7,1)-indola-3­(4,3)-pyrazolacyclooctaphane-1^2^-carboxylic Acid (**19**)

The title compound (12 mg, 89% yield) was prepared following general procedure F using the product from step A (14 mg, 0.016 mmol). The crude product was purified by reverse phase HPLC. LCMS (ESI) method 2: RT = 1.352 min, *m*/*z* = 843.9 (M + H). ^1^H NMR (MeOD, 400 MHz): δ 7.91 (s, 1H), 7.74 (d, *J* = 8.8 Hz, 1H), 7.43 (s, 1H), 7.29 (d, *J* = 8.4 Hz, 1H), 7.10 (s, 1H), 6.58 (s, 2H), 4.76 (dd, *J* = 12.4, 5.6 Hz, 1H), 4.56 (dt, *J* = 13.6, 6.0 Hz, 1H), 4.28–4.18 (m, 4H), 4.14 (d, *J* = 10.0 Hz, 1H), 4.08 (s, 3H), 4.04 (s, 3H), 4.02–3.85 (m, 3H), 3.79–3.73 (m, 2H), 3.70 (d, *J* = 10.0 Hz, 1H), 3.59 (d, *J* = 12.4 Hz, 2H), 3.46 (s, 3H), 3.43–3.36 (m, 1H), 3.08 (t, *J* = 10.0 Hz, 1H), 2.27 (s, 6H), 2.19–2.08 (m, 3H), 1.67 (t, *J* = 11.2 Hz, 1H), 1.15 (d, *J* = 6.8 Hz, 3H).

#### (2^6^3^4^ *S*_*a*_,1^7^2^2^ *R*_*a*_,2^4^ *R*)-2^7^-Chloro-2^10^-(3-(4-chloro-3,5-dimethylphenoxy)­propyl)-1^4^-methoxy-2^4^,3^1^-dimethyl-2^1^-oxo-1^5^-((tetrahydro-2*H*-pyran-4-yl)­methoxy)-2^1^,2^2^,2^3^,2^4^-tetrahydro-1^1^ *H*,3^1^ *H*-5-oxa-2­(2,6)-pyrazino­[1,2-*a*]­indola-1­(7,1)-indola-3­(4,3)-pyrazolacyclooctaphane-1^2^-carboxylic Acid (**20**)

##### Step A: Ethyl (2^6^3^4^ *S*_*a*_,1^7^2^2^ *R*_*a*_,2^4^ *R*)-2^7^-Chloro-2^10^-(3-(4-chloro-3,5-dimethylphenoxy) propyl)-1^4^-methoxy-2^4^,3^1^-dimethyl-2^1^-oxo-1^5^-((tetrahydro-2*H*-pyran-4-yl)­methoxy)-2^1^,2^2^,2^3^,2^4^-tetrahydro-1^1^ *H*,3^1^ *H*-5-oxa-2­(2,6)-pyrazino­[1,2-*a*]­indola-1­(7,1)-indola-3­(4,3)-pyrazolacyclooctaphane-1^2^-carboxylate

Compound **48** (250 mg, 0.310 mmol, 1.0 equiv) was dissolved in DMF (5 mL). Cs_2_CO_3_ (450 mg, 1.40 mmol, 4.5 equiv) was added, followed by (tetrahydro-2*H*-pyran-4-yl)­methyl 4-methylbenzenesulfonate (165 mg, 0.62 mmol, 2.0 equiv). The reaction was heated to 80 °C and allowed to stir for 6 h. The reaction was extracted with EtOAc, washed with H_2_O, brine, dried over MgSO_4_, filtered, and concentrated. The crude material was purified by flash column chromatography eluting with 0 to 100% EtOAc in hexanes to afford the title compound (226 mg, 81% yield). LCMS (ESI) method 2: RT = 1.917 min, *m*/*z* = 912.0 (M + H)^+^.

##### Step B: (2^6^3^4^ *S*_*a*_,1^7^2^2^ *R*_*a*_,2^4^ *R*)-2^7^-Chloro-2^10^-(3-(4-chloro-3,5-dimethylphenoxy)­propyl)-1^4^-methoxy-2^4^,3^1^-dimethyl-2^1^-oxo-1^5^-((tetrahydro-2*H*-pyran-4-yl)­methoxy)-2^1^,2^2^,2^3^,2^4^-tetrahydro-1^1^ *H*,3^1^ *H*-5-oxa-2­(2,6)-pyrazino­[1,2-*a*]­indola-1­(7,1)-indola-3­(4,3)-pyrazola-cyclooctaphane-1^2^-carboxylic Acid (**20**)

The title compound (210 mg, 96% yield) was prepared following general Procedure F using the product from step A (226 mg, 0.25 mmol). LCMS (ESI) method 2: RT = 1.455 min, *m*/*z* = 884.0 (M + H)^+^. ^1^H NMR (MeOD, 400 MHz): δ 7.90 (s, 1H), 7.73 (d, *J* = 8.8 Hz, 1H), 7.42 (s, 1H), 7.29 (d, *J* = 8.8 Hz, 1H), 7.07 (s, 1H), 6.57 (s, 2H), 4.74 (dd, *J* = 12.4, 5.6 Hz, 1H), 4.57 (dt, *J* = 13.6, 6.0 Hz, 1H), 4.27–4.15 (m, 2H), 4.13 (d, *J* = 10.0 Hz, 1H), 4.08–4.01 (m, 5H), 4.00–3.97 (m, 1H), 3.96 (d, *J* = 6.0 Hz, 2H), 3.93–3.85 (m, 2H), 3.69 (d, *J* = 10.0 Hz, 1H), 3.61–3.54 (m, 2H), 3.53–3.46 (m, 2H), 3.43–3.28 (m, 4H), 3.07 (t, *J* = 10.0 Hz, 1H), 2.26 (s, 6H), 2.18–2.04 (m, 4H), 1.87–1.76 (m, 2H), 1.64 (q, *J* = 11.2 Hz, 1H), 1.51 (qt, *J* = 12.4, 4.4 Hz, 2H), 1.14 (d, *J* = 6.8 Hz, 3H).

#### (2^6^3^4^ *S*_*a*_,1^7^2^2^ *R*_*a*_,2^4^ *R*)-2^7^-Chloro-2^10^-(3-(4-chloro-3,5-dimethylphenoxy)­propyl)-1^4^,1^5^-dimethoxy-2^4^-methyl-2^1^-oxo-2^1^,2^2^,2^3^,2^4^-tetrahydro-1^1^ *H*-5-oxa-2­(2,6)-pyrazino­[1,2-*a*]­indola-1­(7,1)-indola-3­(3,2)-pyridinacyclooctaphane-1^2^-carboxylic Acid (**21**)

The title compound was prepared analogously to compound **13**, substituting (4-bromo-1-methyl-1*H*-pyrazol-3-yl)­methanol with (3-bromopyridin-2-yl)­methanol. The complete synthesis is reported in the Supporting Information. The crude product was purified by reverse phase HPLC eluting with MeCN/H_2_O/TFA. The fractions containing product were diluted into DCM, neutralized with saturated NaHCO_3_ solution, and concentrated to afford the title compound. LCMS (ESI) method 1: RT = 2.237 min, *m*/*z* = 797.2 [M + H]^+^. ^1^H NMR (MeOD, 400 MHz): δ 8.76 (dd, *J* = 5.2, 2.0 Hz, 1H), 8.25 (dd, *J* = 7.6 Hz, 1.2 Hz, 1H), 7.80–7.41 (m, 2H), 7.32 (s, 1H), 7.28 (d, *J* = 8.0 Hz, 1H), 6.99 (s, 1H), 6.51 (s, 2H), 4.48–4.37 (m, 2H), 4.09 (d, *J* = 9.2 Hz, 1H), 4.01 (dt, *J* = 13.6, 3.2 Hz, 1H), 3.94 (s, 3H), 3.86–3.79 (m, 5H), 3.75 (d, *J* = 9.2 Hz, 1H), 3.63–3.57 (m, 1H), 3.51 (quint, *J* = 6.0 Hz, 1H), 3.45 (d, *J* = 12.4 Hz, 1H), 3.37–3.25 (m, 2H), 2.94 (t, *J* = 10.0 Hz, 1H), 2.18 (s, 6H), 2.12–2.02 (m, 3H), 1.55 (quart., *J* = 13.2 Hz, 1H), 1.06 (d, *J* = 6.8 Hz, 3H).

#### (2^6^3^4^ *S*_*a*_,1^7^2^2^ *R*_*a*_,2^4^ *R*)-2^7^-Chloro-2^10^-(3-(4-chloro-3,5-dimethylphenoxy)­propyl)-1^4^,1^5^-dimethoxy-2^4^-methyl-2^1^-oxo-2^1^,2^2^,23,24-tetrahydro-11*H*-5-oxa-2­(2,6)-pyrazino­[1,2-*a*]­indola-1­(7,1)-indola-3­(3,4)-pyridinacyclooctaphane-1^2^-carboxylic Acid (**22**)

The title compound was prepared analogously to compound **13**, substituting (4-bromo-1-methyl-1*H*-pyrazol-3-yl)­methanol with (3-bromopyridin-4-yl)­methanol. The complete synthesis is reported in the Supporting Information. The crude product was purified by reverse phase HPLC eluting with MeCN/H_2_O/TFA. The fractions containing product were diluted into DCM, neutralized with saturated NaHCO_3_ solution, and concentrated to afford the title compound. LCMS (ESI) method 1: RT = 2.092 min, *m*/*z* = 797.2 [M + H]^+^. ^1^H NMR (MeOD, 400 MHz): δ 8.78 (s, 1H), 8.74 (d, *J* = 5.2 Hz, 1H), 7.86 (d, *J* = 8.8 Hz, 1H), 7.66 (d, *J* = 5.2 Hz, 1H), 7.37–7.33 (m, 2H), 7.05 (s, 1H), 6.61 (s, 2H), 4.61–4.57 (m, 1H), 4.55–4.49 (m, 1H), 4.07–4.03 (m, 2H), 4.02 (s, 3H), 3.97–3.92 (m, 2H), 3.91 (s, 3H), 3.89–3.84 (m, 2H), 3.67–3.63 (m, 1H), 3.54 (d, *J* = 12.4 Hz, 1H), 3.51–3.38 (m, 2H), 2.99 (t, *J* = 9.6 Hz, 1H), 2.28 (s, 6H), 2.24–2.08 (m, 3H), 1.65–1.56 (m, 1H), 1.17 (d, *J* = 6.8 Hz, 3H).

#### (2^6^3^4^ *R*_*a*_,1^7^2^2^ *R*_*a*_,2^4^ *R*)-2^10^-(3-(4-Chloro-3,5-dimethylphenoxy)­propyl)-1^4^,1^5^-dimethoxy-2^4^,3^1^-dimethyl-2^1^-oxo-2^1^,2^2^,2^3^,2^4^-tetrahydro-1^1^ *H*,3^1^ *H*-5-oxa-2­(2,6)-pyrazino­[1,2-*a*]­indola-1­(7,1)-indola-3­(4,3)-pyrazolacyclooctaphane-1^2^-carboxylic Acid (**23**)

The title compound was prepared analogously to compound **13**, substituting compound **26** with ethyl 7-bromo-3-(3-(4-chloro-3,5-dimethylphenoxy)­propyl)-1*H*-indole-2-carboxylate.^51^ The complete synthesis is reported in the Supporting Information. LCMS (ESI) method 2: RT = 1.442 min, *m*/*z* = 766.3 (M + H). ^1^H NMR (DMSO-*d*_6_, 400 MHz): δ 7.96 (s, 1H), 7.73 (d, *J* = 8.0 Hz, 1H), 7.29 (s, 1H), 7.17 (t, *J* = 7.2 Hz, 1H), 7.14 (s, 1H), 7.07 (d, *J* = 7.2 Hz, 1H), 6.67 (s, 2H), 4.58–4.43 (m, 3H), 4.40–4.33 (m, 1H), 4.26–4.16 (M, 2H), 4.08 (d, *J* = 9.2 Hz, 1H), 3.95 (s, 3H), 3.94 (s, 3H), 3.93–3.89 (m, 1H), 3.86 (s, 3H), 3.64 (d, *J* = 10.0 Hz, 2H), 3.30–3.22 (m, 2H), 3.02 (t, *J* = 10.0 Hz, 1H), 2.22 (s, 6H), 2.07–1.94 (m, 3H), 1.66–1.55 (m, 1H), 1.02 (d, *J* = 6.4 Hz, 3H).

#### (*R*)-7-(7-chloro-10-(3-(4-chloro-3,5-dimethylphenoxy)­propyl)-4-methyl-1-oxo-6-(2,4,6-trimethylpyrimidin-5-yl)-3,4-dihydropyrazino­[1,2-*a*]­indol-2­(1*H*)-yl)-4-methoxy-*N*,*N*,1-trimethyl-5-((tetrahydro-2*H*-pyran-4-yl)­methoxy)-1*H*-indole-2-carboxamide (**24**)

In a reaction vessel, compound **1** (56 mg, 0.064 mmol, 1.0 equiv) was dissolved in DMF (2 mL). DIPEA (48 mg, 0.38 mmol, 6.0 equiv) and HATU (35 mg, 0.092 mmol, 1.5 equiv) were added, and the reaction was allowed to stir for 5 min. Dimethylamine hydrochloride (20 mg, 0.24 mmol, 4.0 equiv) was added, and the reaction was allowed to stir overnight at room temperature. The reaction was extracted with DCM, washed with H_2_O, dried over MgSO_4_, filtered and concentrated. The crude residue was purified by flash column chromatography eluting with 0 to 100% EtOAc in hexanes to afford the title compound (32 mg, 55% yield). LCMS (ESI) method 2: RT = 1.641 min, *m*/*z* = 895.1 (M + H). ^1^H NMR (MeOD, 400 MHz): δ 7.90 (d, *J* = 8.8 Hz, 0.34H), 7.89 (d, *J* = 8.8 Hz, 0.66H), 7.38 (d, *J* = 8.8 Hz, 0.34H), 7.37 (d, *J* = 8.8 Hz, 0.66H), 7.02 (s, 0.34H), 6.85 (s, 0.66H), 6.78 (s, 0.66H), 6.73 (s, 0.34H), 6.63 (s, 1.3H), 6.60 (s, 0.7H), 4.61 (dd, *J* = 12.8, 4.4 Hz, 0.3H), 4.37 (dd, *J* = 13.2, 6.8 Hz, 0.7H), 4.01 (s, 2H), 4.00–3.98 (m, 2H), 3.98–3.93 (m, 3H), 3.92–3.84 (m, 3H), 3.78 (s, 1H), 3.64 (s, 2.33H), 3.61–3.58 (m, 0.66H), 3.52–3.35 (m, 4H), 3.16 (br s, 3H), 3.14 (br s, 3H), 2.79 (s, 3H), 2.43 (s, 1H), 2.40 (s, 2H), 2.28 (s, 3.3H), 2.70 (s, 3H), 2.25 (s, 2H), 2.21–2.12 (m, 2.4H), 2.11–2.00 (m, 1.3H), 1.83–1.72 (m, 2H), 1.54–1.40 (m, 2H), 1.31 (d, *J* = 6.0 Hz, 2H), 1.21 (d, *J* = 6.0 Hz, 1H).

#### (2^6^3^4^ *S*_*a*_,1^7^2^2^ *R*_*a*_,2^4^ *R*)-2^7^-chloro-2^10^-(3-(4-chloro-3,5-dimethylphenoxy)­propyl)-1^4^,1^5^-dimethoxy-*N*,*N*,2^4^,3^1^-tetramethyl-2^1^-oxo-2^1^,2^2^,2^3^,2^4^-tetrahydro-1^1^ *H*,3^1^ *H*-5-oxa-2­(2,6)-pyrazino­[1,2-*a*]­indola-1­(7,1)-indola-3­(4,3)-pyrazolacyclooctaphane-1^2^-carboxamide (**25**)

In a reaction vessel, compound **13** (15 mg, 0.019 mmol, 1.0 equiv) was dissolved in DMF (2 mL). DIPEA (15 mg, 0.11 mmol, 6.0 equiv) and HATU (14 mg, 0.038 mmol, 2.0 equiv) were added, and the reaction was allowed to stir for 5 min. Dimethylamine hydrochloride (6 mg, 0.075 mmol, 4.0 equiv) was added, and the reaction was allowed to stir overnight at room temperature. The reaction was extracted with DCM, washed with H_2_O, dried over MgSO_4_, filtered and concentrated. The crude residue was purified by flash column chromatography eluting with 0 to 100% EtOAc in hexanes to afford the title compound (6 mg, 38% yield). LCMS (ESI) method 2: RT = 1.281 min, *m*/*z* = 766.0 (M + H). ^1^H NMR (CDCl_3_, 400 MHz): δ 7.67 (d, *J* = 8.4 Hz, 1H), 7.61 (s, 1H), 7.30 (s, 1H), 6.88 (s, 1H), 6.77 (s, 1H), 6.60 (s, 2H), 4.77 (dd, *J* = 12.0, 5.6 Hz, 1H), 4.26–4.14 (m, 4H), 4.08 (s, 3H), 4.03 (s, 3H), 3.98–3.95 (m, 1H), 3.94 (s, 3H), 3.75 (d, *J* = 10.0 Hz, 1H), 3.64–3.58 (m, 1H), 3.42–3.28 (m, 3H), 3.09 (s, 6H), 3.06–3.03 (m, 1H), 3.83 (s, 1H), 2.33 (s, 6H), 2.20–2.11 (m, 2H), 2.07–1.97 (m, 1H), 1.78 (q, *J* = 12.0 Hz, 1H), 1.13 (d, *J* = 6.8 Hz, 3H).

## Supplementary Material





## Notes

Molecular formula strings (CSV)
X-ray collection data, Glide docking scores of macrocyclic tethers, data from K562 proliferation assay, caspase 3/7 activation in H929, A427, and K562, HPLC-MS traces for compounds **8**–**25**, and complete synthesis of compounds **21**–**23** (PDF)

## ⊥

Molecular Desing and Synthesis Center, Vanderbilt Institute of Chemical Biology, Vanderbilt University, Nashville, TN 37323-0146, USA

## #

AbbVie Bioresearch Center, 381 Plantation St., Worcester, MA. 01605-2323, USA.

## ¶

Cleveland Clinic, Center for Therapeutics Discovery Lerner Research Institute, Cleveland Clinic Foundation, Cleveland, OH. 44106, USA.

## ∇

Department of Molecular Medicine, Cleveland Clinic Lerner College of Medicine, Case Western Reserve University, Cleveland, OH, 44195, USA.

## Abbreviations

APS: Advanced Photon Source
Bcl-2: B-cell lymphoma 2
BH3: BCL-2 homology domain 3
CL: clearance
CLL: chronic lymphocytic leukemia
DTT: dithiothreitol
DLBCL: diffuse large B-cell lymphoma
DIPEA: diisopropylethylamine
ELSD: evaporative light scattering detector
EtOAc: ethyl acetate
*F*: percent oral bioavailability
FITC: fluorescein isothiocyanate
GI_50_: half maximal growth inhibition concentration
HATU: hexafluorophosphate azabenzotriazole tetramethyl uranium
HP-β-CD: hydroxypropyl beta-cyclodextrin
LS-CAT: life sciences collaborative access team
Mcl-1: myeloid cell leukemia 1
MeCN: acetonitrile
MeOH: methanol
MM: multiple myeloma
MOMP: mitochondrial outer membrane permeabilization
NSCLC: nonsmall cell lung cancer
PEG: polyethylene glycol
q14d: once every 14 days
RLU: relative luminescence units
SCLC: small cell lung cancer
TEA: triethylamine
TGI: tumor growth inhibition
RT: retention time
TR-FRET: time-resolved fluorescence resonance energy transfer
TRIS: tris­(hydroxymethyl)­aminomethane
