# Supplementary material for: Discovery of Macrocyclic Myeloid Cell Leukemia 1 (Mcl-1) Inhibitors that Demonstrate Potent Cellular Efficacy and In Vivo Activity in a Mouse Solid Tumor Xenograft Model
Source: J Med Chem. 2025 Aug 27;68(17):18553–78. doi: 10.1021/acs.jmedchem.5c01376 (PMC12434668; doi:10.1021/acs.jmedchem.5c01376)
Supplement: Supplementary file 2 [file jm5c01376_si_002.pdf]

Supporting Information

Discovery of Macrocyclic Myeloid Cell Leukemia 1 (Mcl-1) Inhibitors that Demonstrate Potent Cellular Efficacy and *In Vivo* Activity in a Mouse Solid Tumor Xenograft Model

*James C. Tarr,[1] Kyuok Jeon,[1] Nagarathanam Veerasamy,[1] Martin Aichinger,[2] James M. Salovich,[1] Bin Zhao,[1] John L. Sensintaffar,[1] Heribert Arnhof,[2] Tobias Wunberg,[2] Danielle Sgubin,[1] Allison Arnold,[1] Rakesh H. Vekariya,[1] Plamen P. Christov,[3] Kwangho Kim,[3] Julian Emanuel Fuchs,[2] Pol Karier[4] Bodo Betzemeier,[4] Mayme Van Meveren,[1] Nagaraju Miriyala,[1] Edward T. Olejniczak,[1] Harald Engelhardt,[2] Taekyu Lee,[1] Darryl McConnell,[2] Stephen W. Fesik[1]**

1. Department of Biochemistry, Vanderbilt University School of Medicine, 2215 Garland Avenue, 607 Light Hall, Nashville, TN 37232-0146, United States.

2. Discovery Research, Boehringer Ingelheim Regional Center Vienna GmbH & Co KG, 1120 Vienna, Austria.

3. Molecular Design and Synthesis Center, Vanderbilt Institute of Chemical Biology, Vanderbilt University, Nashville, TN 37323-0146, United States.

4. Boehringer Ingelheim Pharma GmbH & Co. KG, Chemical Development Germany, Birkendorfer Straße 65, 88397, Biberach an der Riß, Germany

* *Corresponding Author: stephen.fesik@vanderbilt.edu*

Table of Contents

S1. X-ray Collection Data and Refinement Statistics (**8**, **13**) (S3)

S2. GLIDE Docking Scores of Various Tether Links (S4)

S3. Tabulation of GI_{50} in Cell Proliferation Assay for H929, A427, and K562 Cell Lines (S5)

S4. Tabulation of Caspase 3/7 EC_{50} and Fold Change in H929, A427, and K562 Cell Lines (S6)

S5. HPLC-MS for compounds **8**-**25** (S7-S23)

S6. Synthesis of Intermediates, compounds **21**, **22**, and **23** (S24-S40)

Table S1. X-ray data collection and refinement statistics for compounds **8** and **13**.

Compound<br>PDB Accession code	**8**	**13**
**Data Collection**		
Space Group	P 2_1	P 2_1 2_1 2
Cell Dimensions		
a, b, c (Å)	38.351, 86.865, 49.637	97.787, 136.366, 38.440
α, β, γ (°)	90.00, 102.60, 90.00	90.00, 90.00, 90.00
Resolution (Å)	28.38-2.06 (2.10-2.06)	29.50-1.95 (1.98-1.95)
R_{merge} (%)	0.078 (0.357)	0.110 (0.511)
Mean I / σI	12.2 (1.9)	20.4 (2.3)
Completeness (%)	97.79 (94)	99.52 (99)
Redundancy	2.2 (1.6)	7.0 (5.2)
**Structure Refinement**		
No. Reflections	18,204	38,279
R_{work}/R_{free}	0.1954/0.2433	0.1909/0.2229
R.m.s. deviations		
Bond lengths (%)	0.008	0.003
Bond angles (%)	1.076	0.647
Ramachandran		
Preferred regions (%)	94.95	97.83
Allowed regions (%)	4.38	2.17
Disallowed regions (%)	0.67	0

*High resolution shells are in parentheses.

S2: Glide Docking Scores of Various Tether Links

Structure	Compound #	# Atoms in Tether	Glide Docking Score[a]
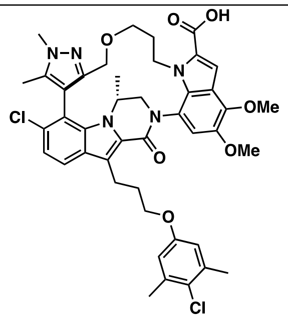	**10**	5	-13.948
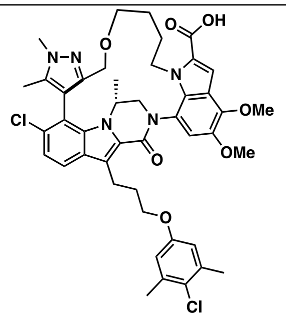	**12**	6	-10.010
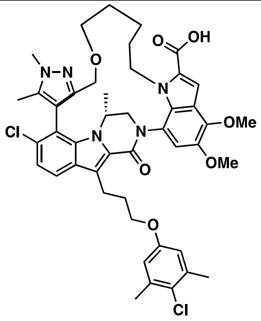	Not synthesized	7	-12.857
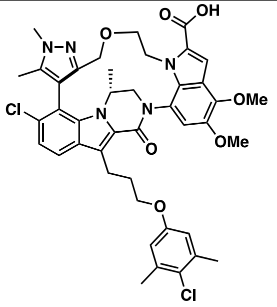	Not synthesized	4	-13.349

a. Docking score generated using Schodinger Maestro GLIDE function.

Table S3: Comparison of GI_{50} in Mcl-1 sensitive (H929, A427) and Mcl-1 insensitive (K562) tumor cell lines

Entry	H929 GI_{50} (nM)	A427 GI_{50} (nM)	K562 GI_{50} (nM)
**10**	20±4	48±16	>9700±4200
**11**	24±6	64±20	>12500
**12**	39±10	87±60	>7800±3500
**13**	14±4	25±9	>12500
**14**	64±17	195±14	>10200
**15**	38±11	60±24	>12500
**16**	27±3	45±17	>12500
**17**	23±5	32±7	>12500
**18**	35±16	51±20	>12500
**19**	10±3	18±6	>11200±2500
**20**	31±13	55±26	>12500
**21**	17±8	39±7	>12500
**22**	29±6	58±4	>12500
**23**	15±4	32±4	>12500
**24**	1100±380	3600±140	>12500
**25**	39±7	105±24	>12500

All data points are an average of at least n = 2, run in triplicate. Top concentration tested for each compound was 12.5 µM.

Table S4: Caspase 3/7 Activity in H929, A427, and K562

Entry	H929 EC_{50} (nM)[a]	H929 FoV[b]	A427 EC_{50} (nM)[a]	A427 FoV[b]	K562 EC_{50} (nM)[a]	K562 FoV[b]
**1**	8.8	16	11	13	>3125	1.4
**10**	9	16	11	13	>3125	1.4
**13**	13	16	7.8	16	>3125	1.3
**14**	45	17	35	16	>3125	1.3
**15**	43	17	34	15	>3125	1.3
**16**	16	16	24	15	>3125	1.3
**17**	16	16	12	16	>3125	1.3
**18**	36	17	28	16	>3125	1.4
**19**	11	16	13	14	>3125	1.4
**20**	23	16	31	14	>3125	1.3
**21**	12	16	17	14	>3125	1.4
**25**	17	16	24	14	>3125	1.4

a. All data points are an average of at least n = 2, run in triplicate. Top concentration tested for each compound was 12.5 μM. b) Fold over vehicle.

S5. LCMS Spectra of Final Compounds

Compound 8

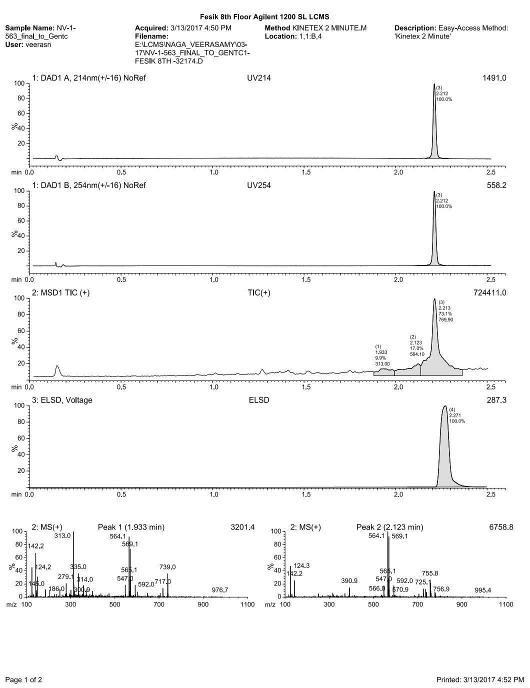

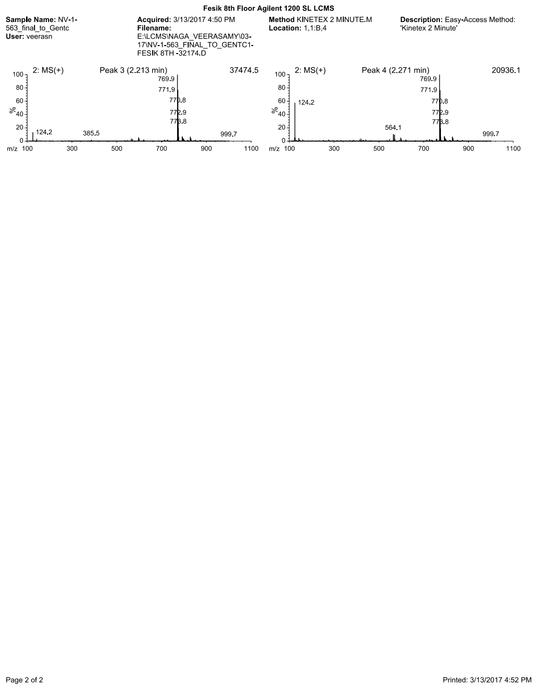

Compound 10

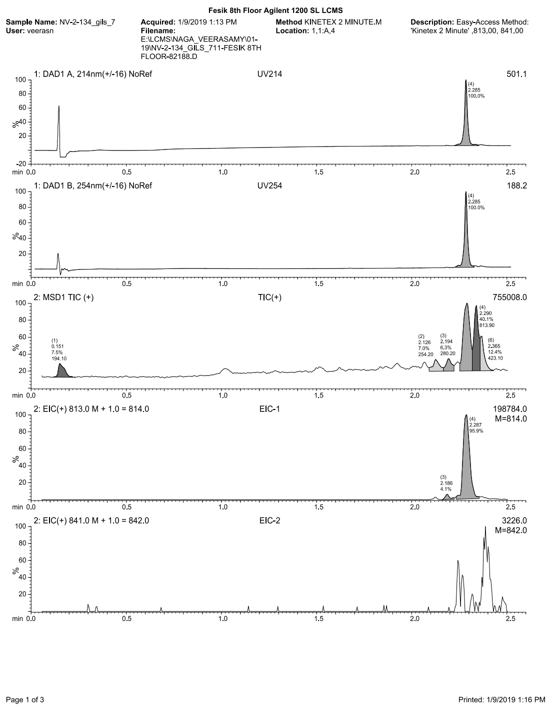

Fesik 8th Floor Agilent 1200 SL LCMS
Sample Name: NV-2-134_gils_7
User: veerasn
Acquired: 1/9/2019 1:13 PM
Filename: E:\LCMS\NAGA_VEERASAMY\01-19\NV-2-134_GILS_711-FESIK 8TH FLOOR-82188.D
Method KINETEX 2 MINUTE.M
Location: 1,1:A,4
Description: Easy-Access Method: 'Kinetex 2 Minute' ,813.00, 841.00
1: DAD1 A, 214nm(+/-16) NoRef
UV214
501.1
1: DAD1 B, 254nm(+/-16) NoRef
UV254
188.2
2: MSD1 TIC (+)
TIC(+)
755008.0
2: EIC(+) 813.0 M + 1.0 = 814.0
EIC-1
198784.0
M=814.0
2: EIC(+) 841.0 M + 1.0 = 842.0
EIC-2
3226.0
M=842.0
Page 1 of 3
Printed: 1/9/2019 1:16 PM

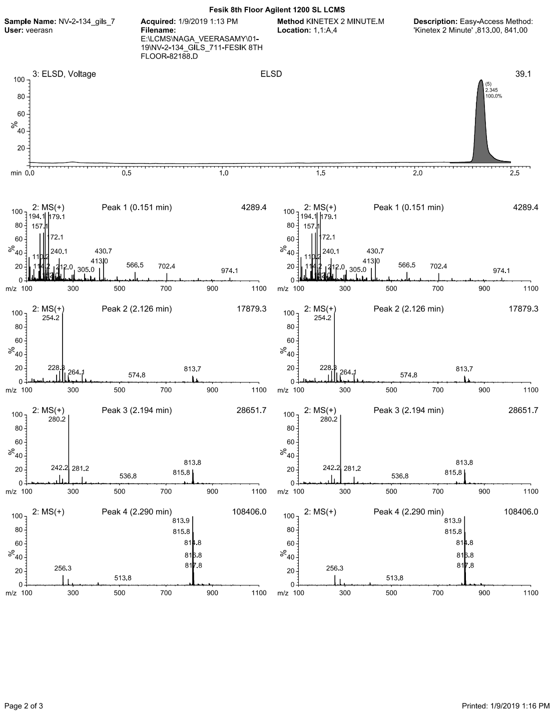

Fesik 8th Floor Agilent 1200 SL LCMS
Sample Name: NV-2-134_gils_7
User: veerasn
Acquired: 1/9/2019 1:13 PM
Filename: E:\LCMS\NAGA_VEERASAMY\01-19\NV-2-134_GILS_711-FESIK 8TH FLOOR-82188.D
Method KINETEX 2 MINUTE.M
Location: 1,1:A,4
Description: Easy-Access Method: 'Kinetex 2 Minute' ,813.00, 841.00
3: ELSD, Voltage
ELSD
39.1
2: MS(+) Peak 1 (0.151 min) 4289.4
2: MS(+) Peak 2 (2.126 min) 17879.3
2: MS(+) Peak 3 (2.194 min) 28651.7
2: MS(+) Peak 4 (2.290 min) 108406.0
Page 2 of 3
Printed: 1/9/2019 1:16 PM

Compound 11

VCNDD/Syncore MRB4-12410 Agilent 1200 LCMS

Sample Name: KK-27-023-BS
User: kimk3

Acquired: 4/4/2019 1:36 PM
Filename: C:\LCMS\DATA\KWANGHO_KIM\04-19\KK-27-023-BS11-12410 SYNCORE LCMS-.D

Method 1 MIN NONPOLAR HIGH MASS.M
Location: 1,2:E,2

Description: Easy-Access Method: '1 Minute Nonpolar High Mass' ,814.00, 815.00

Peak #	Time	Area % UV215	Area % UV254	Area % TIC(+)	BPM	Area Abs UV215	Area Abs UV254	Area Abs TIC(+)
1	0.093	0.3	2.8	13.8	413.5	12.78	11.73	247419.58
1	0.093	0.3	2.8	13.8	413.5	12.78	11.73	247419.58
2	0.145	73.8	0.0	0.0	530.7	3031.69	0	0
2	0.145	73.8	0.0	0.0	530.7	3031.69	0	0
3	0.983	25.5	97.2	65.9	814.6	1045.64	404.73	1178191.63
3	0.983	25.5	97.2	65.9	814.6	1045.64	404.73	1178191.63
4	1.134	0.4	0.0	13.2	828.6	16.19	0	236062.94
4	1.134	0.4	0.0	13.2	828.6	16.19	0	236062.94
5	1.287	0.0	0.0	7.0	391.6	0	0	125476.29
5	1.287	0.0	0.0	7.0	391.6	0	0	125476.29

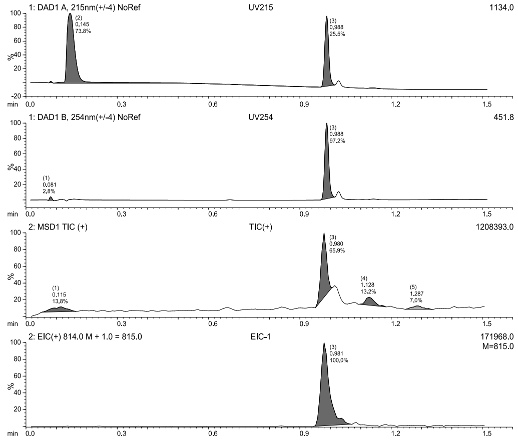

Page 1 of 3

Printed: 4/4/2019 1:38 PM

VCNDD/Syncore MRB4-12410 Agilent 1200 LCMS

Sample Name: KK-27-023-BS
User: kimk3

Acquired: 4/4/2019 1:36 PM
Filename: C:\LCMS\DATA\KWANGHO_KIM\04-19\KK-27-023-BS11-12410 SYNCORE LCMS-.D

Method 1 MIN NONPOLAR HIGH MASS.M
Location: 1,2:E,2

Description: Easy-Access Method: '1 Minute Nonpolar High Mass' ,814.00, 815.00

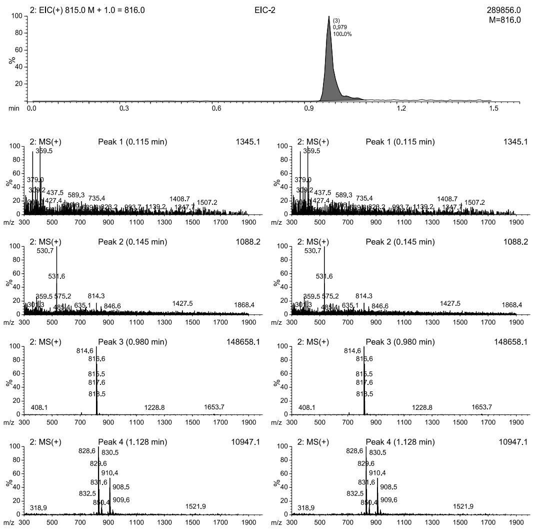

Page 2 of 3

Printed: 4/4/2019 1:38 PM

Compound 12

Fesik 8th Floor Agilent 1200 SL LCMS

Sample Name: NV-2-040_gils_4
User: veerasn
Acquired: 8/14/2018 2:16 PM
Filename: E:\LCMS\NAGA_VEERASAMY\08-18\NV-2-040_GILS_411-FESIK 8TH FLOOR-72629.D
Method ULTRA2_AQUEOUS.M
Location: 1,1:C,4
Description: Easy-Access Method: 'Ultra II Aqueous' ,827.00, 793.00

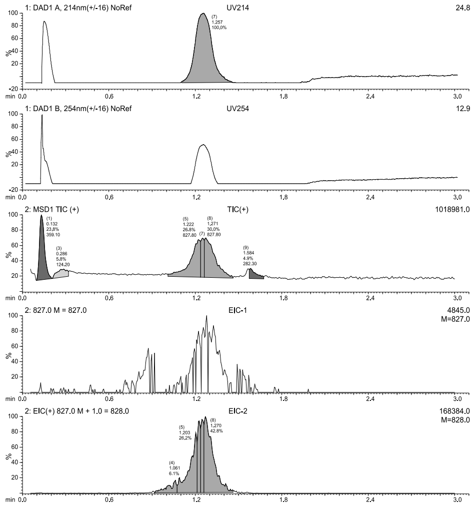

Printed: 8/14/2018 2:20 PM

Fesik 8th Floor Agilent 1200 SL LCMS

Sample Name: NV-2-040_gils_4
User: veerasn
Acquired: 8/14/2018 2:16 PM
Filename: E:\LCMS\NAGA_VEERASAMY\08-18\NV-2-040_GILS_411-FESIK 8TH FLOOR-72629.D
Method ULTRA2_AQUEOUS.M
Location: 1,1:C,4
Description: Easy-Access Method: 'Ultra II Aqueous' ,827.00, 793.00

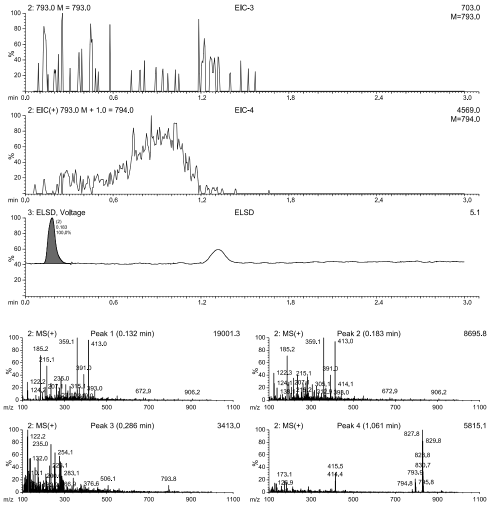

Printed: 8/14/2018 2:20 PM

Fesik 8th Floor Agilent 1200 SL LCMS

Sample Name: NV-2-040_gils_4
User: veerasn
Acquired: 8/14/2018 2:16 PM
Filename: E:\LCMS\NAGA_VEERASAMY\08-18\NV-2-040_GILS_411-FESIK 8TH FLOOR-72629.D
Method ULTRA2_AQUEOUS.M
Location: 1,1:C,4
Description: Easy-Access Method: 'Ultra II Aqueous' ,827.00, 793.00

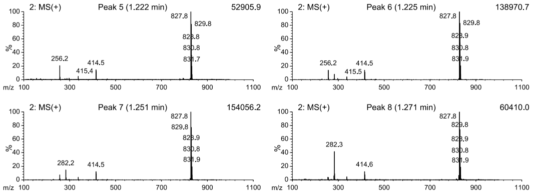

Compound 13

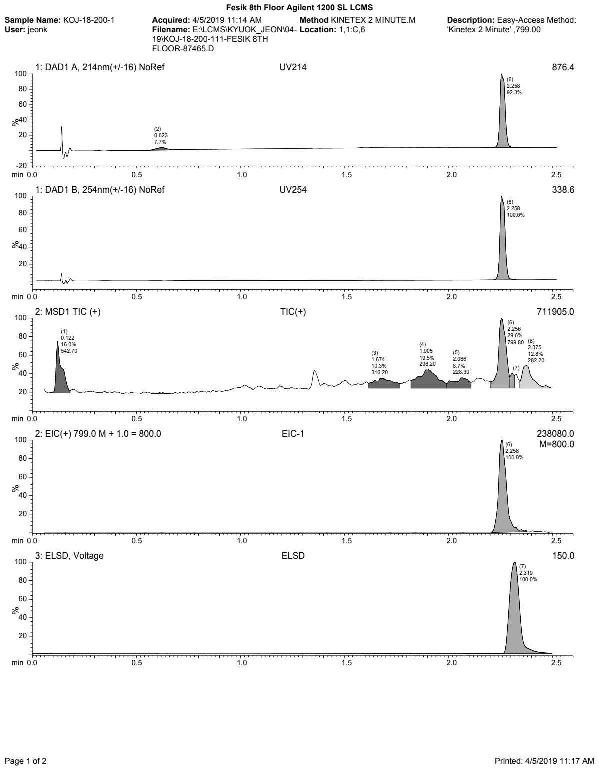
Fesik 8th Floor Agilent 1200 SL LCMS
Sample Name: KOJ-18-200-1
User: jeonk
Acquired: 4/5/2019 11:14 AM
Filename: E:\LCMS\KYUOK_JEON\04-19\KOJ-18-200-111-FESIK 8TH FLOOR-87465.D
Method KINETEX 2 MINUTE.M
Location: 1,1:C,6
Description: Easy-Access Method: 'Kinetex 2 Minute' ,799.00
1: DAD1 A, 214nm(+/-16) NoRef
UV214
876.4
1: DAD1 B, 254nm(+/-16) NoRef
UV254
338.6
2: MSD1 TIC (+)
TIC(+)
711905.0
2: EIC(+) 799.0 M + 1.0 = 800.0
EIC-1
238080.0
M=800.0
3: ELSD, Voltage
ELSD
150.0
Page 1 of 2
Printed: 4/5/2019 11:17 AM

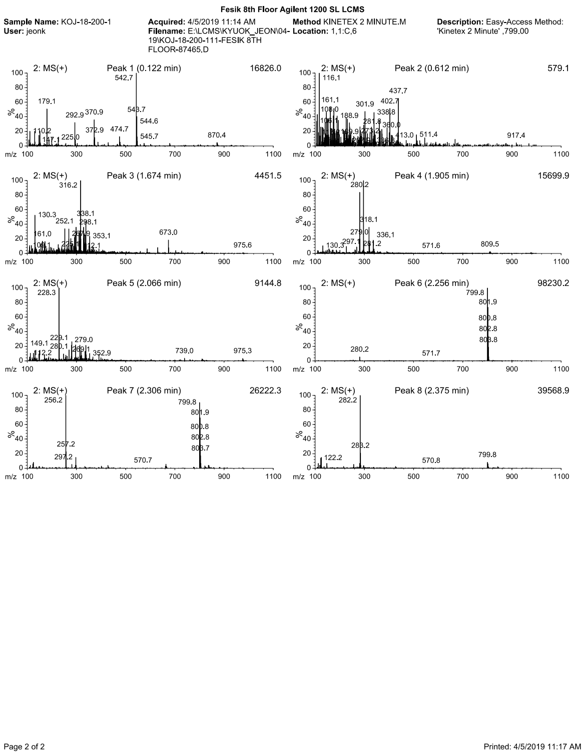
Fesik 8th Floor Agilent 1200 SL LCMS
Sample Name: KOJ-18-200-1
User: jeonk
Acquired: 4/5/2019 11:14 AM
Filename: E:\LCMS\KYUOK_JEON\04-19\KOJ-18-200-111-FESIK 8TH FLOOR-87465.D
Method KINETEX 2 MINUTE.M
Location: 1,1:C,6
Description: Easy-Access Method: 'Kinetex 2 Minute' ,799.00
2: MS(+) Peak 1 (0.122 min) 16826.0
2: MS(+) Peak 2 (0.612 min) 579.1
2: MS(+) Peak 3 (1.674 min) 4451.5
2: MS(+) Peak 4 (1.905 min) 15699.9
2: MS(+) Peak 5 (2.066 min) 9144.8
2: MS(+) Peak 6 (2.256 min) 98230.2
2: MS(+) Peak 7 (2.306 min) 26222.3
2: MS(+) Peak 8 (2.375 min) 39568.9
Page 2 of 2
Printed: 4/5/2019 11:17 AM

Compound 14

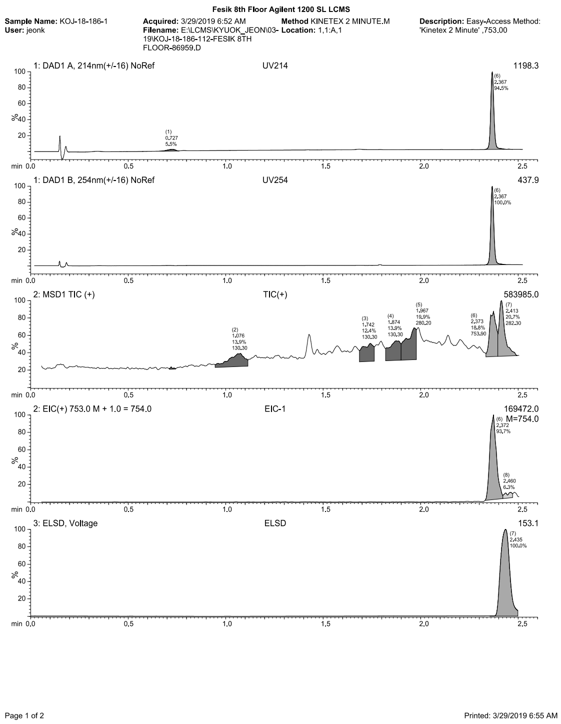
Fesik 8th Floor Agilent 1200 SL LCMS
Sample Name: KOJ-18-186-1
User: jeonk
Acquired: 3/29/2019 6:52 AM
Filename: E:\LCMS\KYUOK_JEON\03-19\KOJ-18-186-112-FESIK 8TH FLOOR-86959.D
Method KINETEX 2 MINUTE,M
Location: 1,1:A,1
Description: Easy-Access Method: 'Kinetex 2 Minute' ,753,00
1: DAD1 A, 214nm(+/-16) NoRef
UV214
1198.3
1: DAD1 B, 254nm(+/-16) NoRef
UV254
437.9
2: MSD1 TIC (+)
TIC(+)
583985.0
2: EIC(+) 753.0 M + 1,0 = 754.0
EIC-1
169472.0
M=754.0
3: ELSD, Voltage
ELSD
153.1
Page 1 of 2
Printed: 3/29/2019 6:55 AM

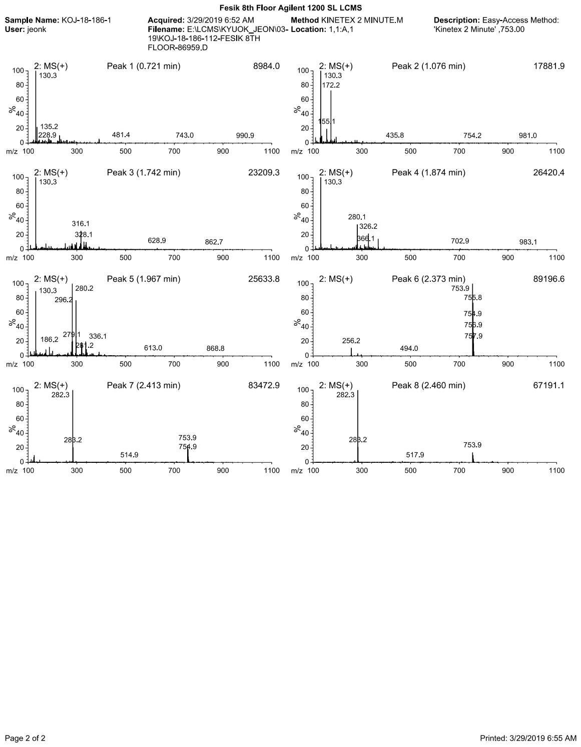
Fesik 8th Floor Agilent 1200 SL LCMS
Sample Name: KOJ-18-186-1
User: jeonk
Acquired: 3/29/2019 6:52 AM
Filename: E:\LCMS\KYUOK_JEON\03-19\KOJ-18-186-112-FESIK 8TH FLOOR-86959.D
Method KINETEX 2 MINUTE,M
Location: 1,1:A,1
Description: Easy-Access Method: 'Kinetex 2 Minute' ,753,00
2: MS(+) Peak 1 (0.721 min) 8984.0
2: MS(+) Peak 2 (1.076 min) 17881.9
2: MS(+) Peak 3 (1.742 min) 23209.3
2: MS(+) Peak 4 (1.874 min) 26420.4
2: MS(+) Peak 5 (1.967 min) 25633.8
2: MS(+) Peak 6 (2.373 min) 89196.6
2: MS(+) Peak 7 (2.413 min) 83472.9
2: MS(+) Peak 8 (2.460 min) 67191.1
Page 2 of 2
Printed: 3/29/2019 6:55 AM

Compound 15

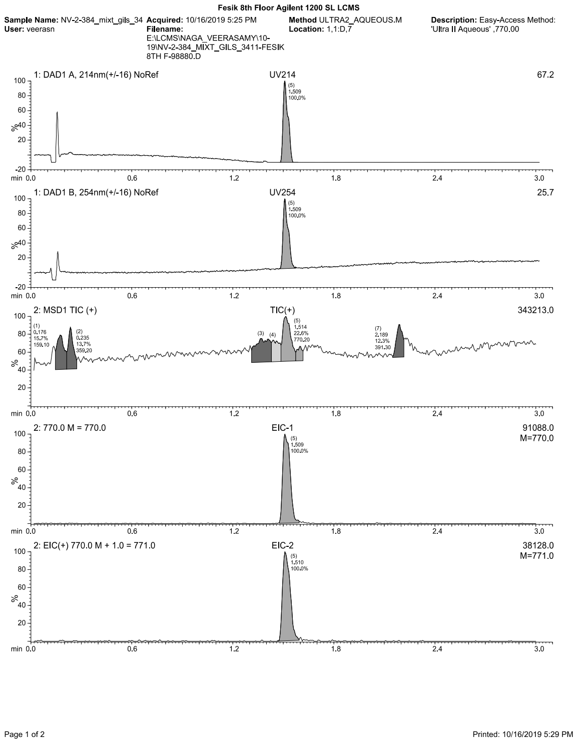

Fesik 8th Floor Agilent 1200 SL LCMS
Sample Name: NV-2-384_mixt_gils_34
User: veerasn
Acquired: 10/16/2019 5:25 PM
Filename: E:\LCMS\NAGA_VEERASAMY\10-19\NV-2-384_MIXT_GILS_3411-FESIK 8TH F-98880.D
Method ULTRA2_AQUEOUS.M
Location: 1,1:D,7
Description: Easy-Access Method: 'Ultra II Aqueous' ,770.00
1: DAD1 A, 214nm(+/-16) NoRef
UV214
67.2
1: DAD1 B, 254nm(+/-16) NoRef
UV254
25.7
2: MSD1 TIC (+)
TIC(+)
343213.0
2: 770.0 M = 770.0
EIC-1
91088.0
M=770.0
2: EIC(+) 770.0 M + 1.0 = 771.0
EIC-2
38128.0
M=771.0
Page 1 of 2
Printed: 10/16/2019 5:29 PM

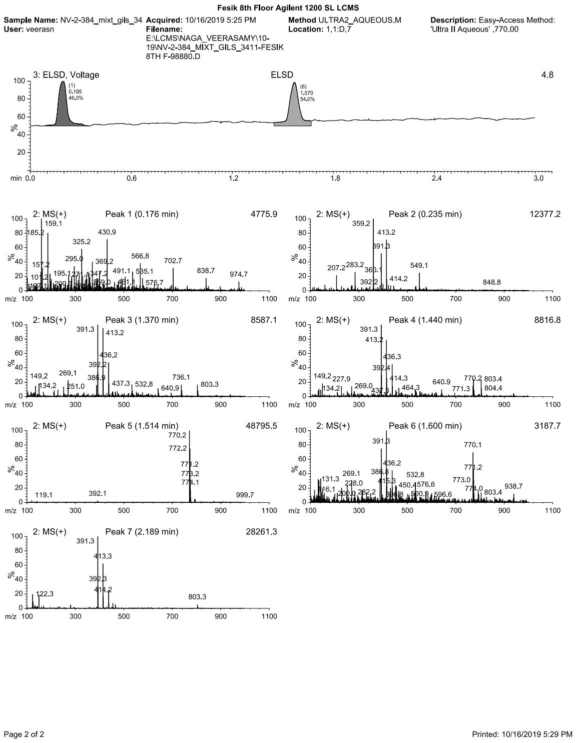

Fesik 8th Floor Agilent 1200 SL LCMS
Sample Name: NV-2-384_mixt_gils_34
User: veerasn
Acquired: 10/16/2019 5:25 PM
Filename: E:\LCMS\NAGA_VEERASAMY\10-19\NV-2-384_MIXT_GILS_3411-FESIK 8TH F-98880.D
Method ULTRA2_AQUEOUS.M
Location: 1,1:D,7
Description: Easy-Access Method: 'Ultra II Aqueous' ,770.00
3: ELSD, Voltage
ELSD
4.8
2: MS(+) Peak 1 (0.176 min) 4775.9
2: MS(+) Peak 2 (0.235 min) 12377.2
2: MS(+) Peak 3 (1.370 min) 8587.1
2: MS(+) Peak 4 (1.440 min) 8816.8
2: MS(+) Peak 5 (1.514 min) 48795.5
2: MS(+) Peak 6 (1.600 min) 3187.7
2: MS(+) Peak 7 (2.189 min) 28261.3
Page 2 of 2
Printed: 10/16/2019 5:29 PM

Compound 16

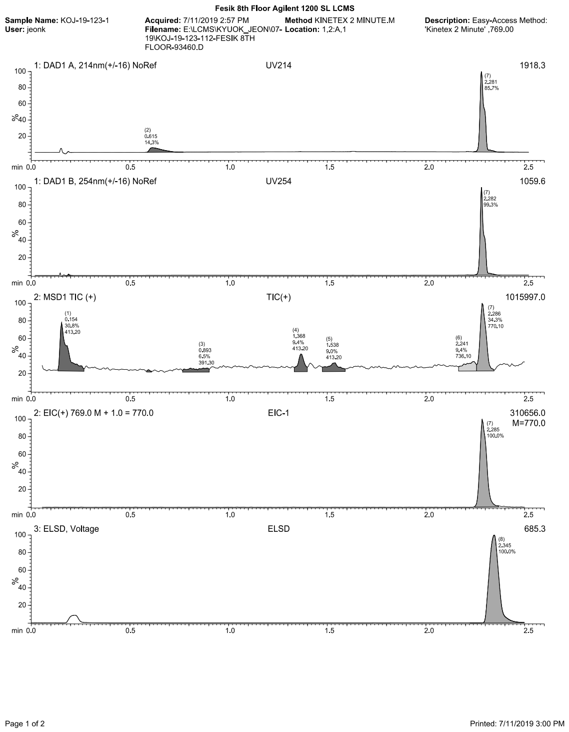
Fesik 8th Floor Agilent 1200 SL LCMS
Sample Name: KOJ-19-123-1
User: jeonk
Acquired: 7/11/2019 2:57 PM
Method KINETEX 2 MINUTE.M
Description: Easy-Access Method: 'Kinetex 2 Minute' ,769.00
1: DAD1 A, 214nm(+/-16) NoRef
UV214
1: DAD1 B, 254nm(+/-16) NoRef
UV254
2: MSD1 TIC (+)
TIC(+)
2: EIC(+) 769.0 M + 1.0 = 770.0
EIC-1
3: ELSD, Voltage
ELSD
Page 1 of 2
Printed: 7/11/2019 3:00 PM

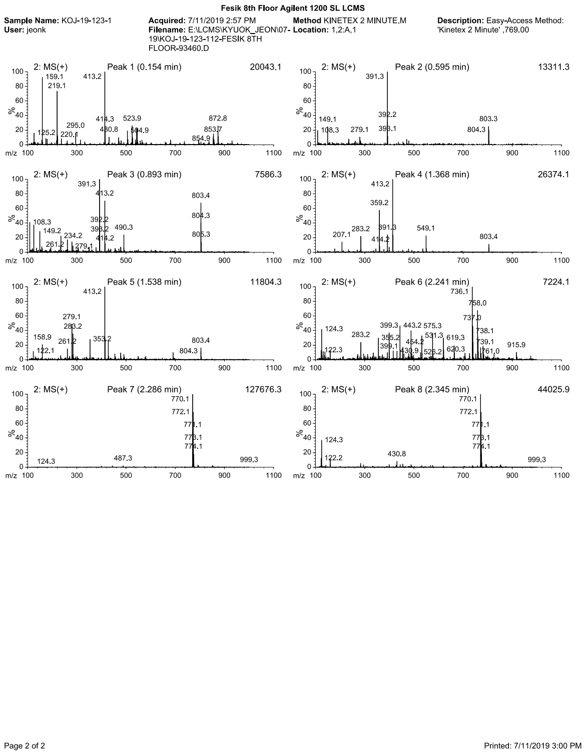
Fesik 8th Floor Agilent 1200 SL LCMS
Sample Name: KOJ-19-123-1
User: jeonk
Peak 1 (0.154 min)
Peak 2 (0.595 min)
Peak 3 (0.893 min)
Peak 4 (1.368 min)
Peak 5 (1.538 min)
Peak 6 (2.241 min)
Peak 7 (2.286 min)
Peak 8 (2.345 min)
Page 2 of 2
Printed: 7/11/2019 3:00 PM

Compound 17

Fesik 8th Floor Agilent 1200 SL LCMS

Sample Name: JMS-Z-152-1FB
User: salovijm
Acquired: 10/5/2019 5:29 AM
Filename: E:\LCMS\JAMES_SALOVICH\10-19\JMS-Z-152-1FB11-FESIK 8TH FLOOR-98158.D
Method KINETEX 2 MINUTE.M
Location: 1,1:E,8
Description: Easy-Access Method: 'Kinetex 2 Minute' ,783.00

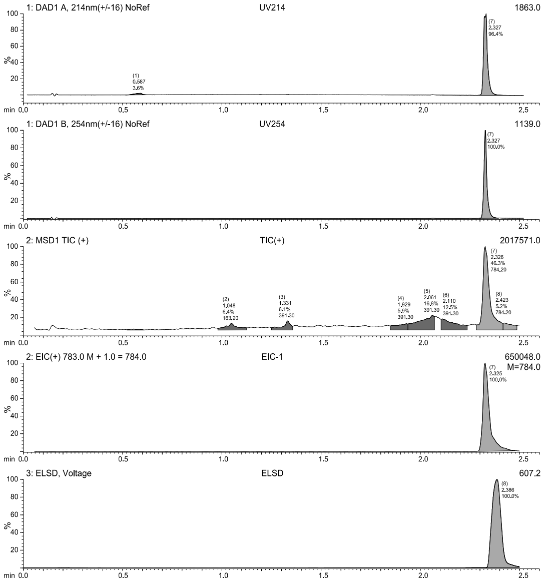

Page 1 of 2 Printed: 3/10/2025 11:03 AM

Fesik 8th Floor Agilent 1200 SL LCMS

Sample Name: JMS-Z-152-1FB
User: salovijm
Acquired: 10/5/2019 5:29 AM
Filename: E:\LCMS\JAMES_SALOVICH\10-19\JMS-Z-152-1FB11-FESIK 8TH FLOOR-98158.D
Method KINETEX 2 MINUTE.M
Location: 1,1:E,8
Description: Easy-Access Method: 'Kinetex 2 Minute' ,783.00

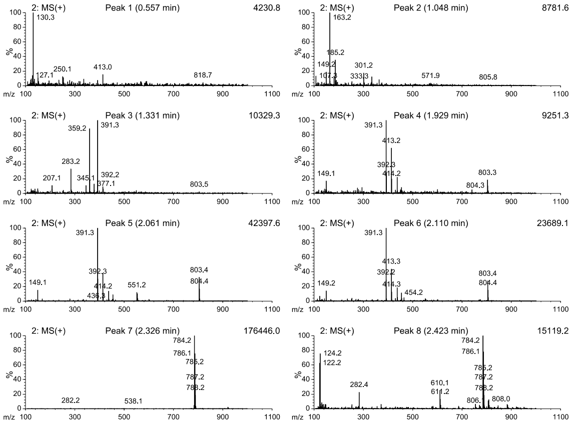

Page 2 of 2 Printed: 3/10/2025 11:03 AM

Compound 18

VCNDD/Syncore MRB4-12410 Agilent 1200 LCMS

Sample Name: KOJ-19-130-GF15
User: jeonk
Acquired: 7/17/2019 4:13 PM
Filename: C:\LCMS\DATA\KYU_JEON\07-19\KOJ-19-130-GF1511-12410 SYNCORE LCMS-.D
Method 1 MIN NONPOLAR.M
Location: 1,1:A,3
Description: Easy-Access Method: '1 Minute Nonpolar' ,783.00

Peak #	Time	Area % UV215	Area % UV254	Area % TIC(+)	BPM	Area Abs UV215	Area Abs UV254	Area Abs TIC(+)
1	0.964	100.0	100.0	100.0	784.6	2128.02	988.00	2959722.00

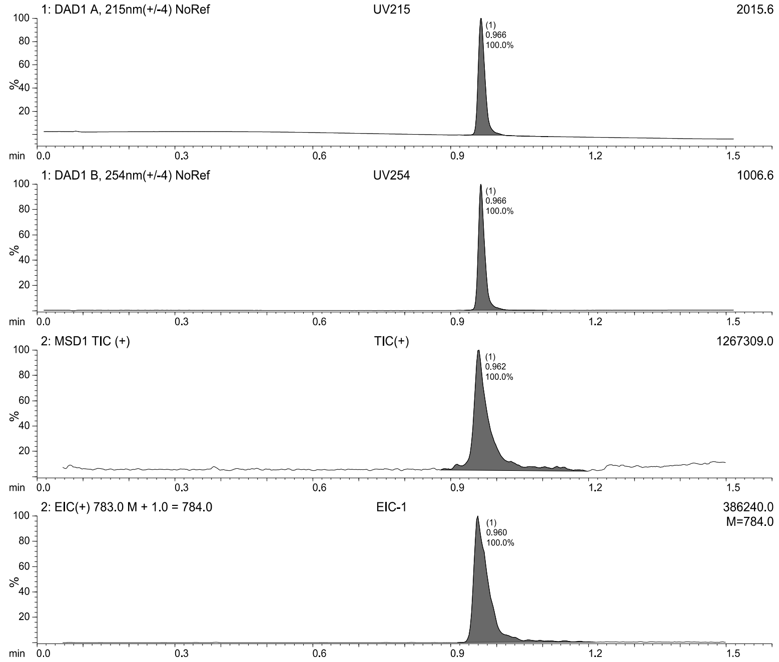

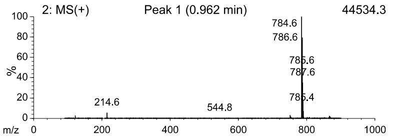

Page 1 of 1

Printed: 7/17/2019 4:15 PM

Compound 19

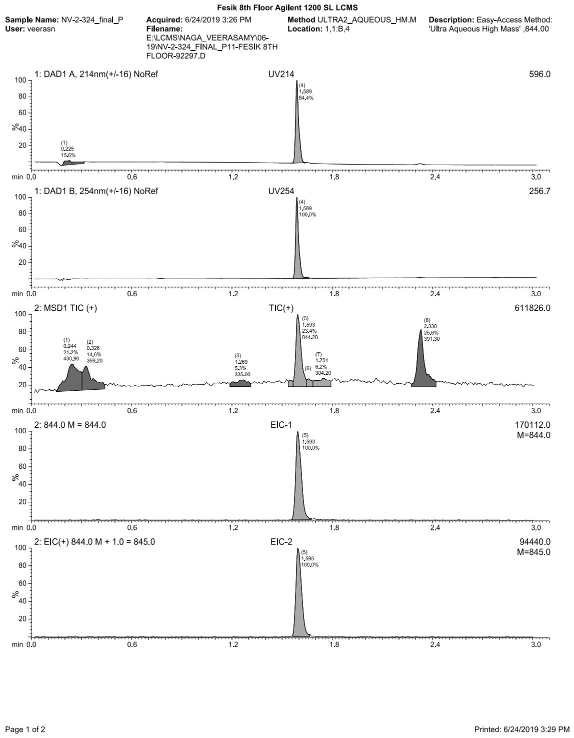
Fesik 8th Floor Agilent 1200 SL LCMS
Sample Name: NV-2-324_final_P
User: veerasn
Acquired: 6/24/2019 3:26 PM
Filename: E:\LCMS\NAGA_VEERASAMY\06-19\NV-2-324_FINAL_P11-FESIK 8TH FLOOR-92297.D
Method ULTRA2_AQUEOUS_HM.M
Location: 1,1:B,4
Description: Easy-Access Method: 'Ultra Aqueous High Mass' ,844.00
1: DAD1 A, 214nm(+/-16) NoRef
UV214
1: DAD1 B, 254nm(+/-16) NoRef
UV254
2: MSD1 TIC (+)
TIC(+)
2: 844.0 M = 844.0
EIC-1
2: EIC(+) 844.0 M + 1.0 = 845.0
EIC-2
Page 1 of 2
Printed: 6/24/2019 3:29 PM

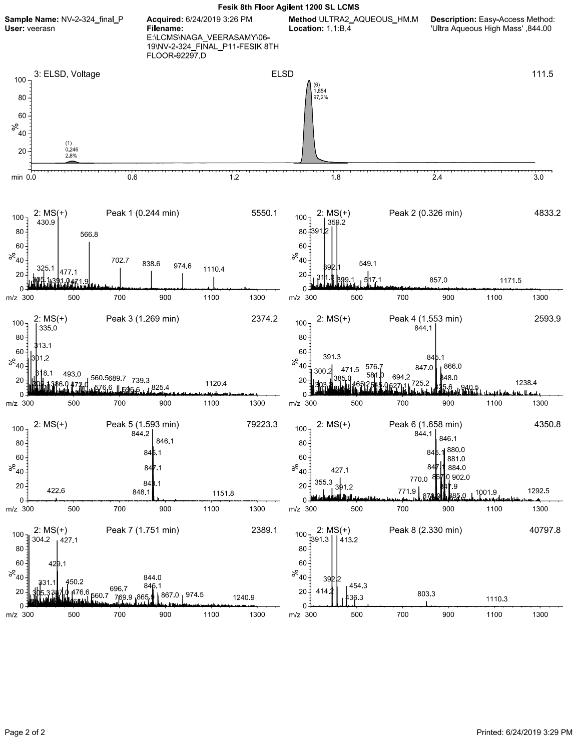
Fesik 8th Floor Agilent 1200 SL LCMS
Sample Name: NV-2-324_final_P
User: veerasn
Acquired: 6/24/2019 3:26 PM
Filename: E:\LCMS\NAGA_VEERASAMY\06-19\NV-2-324_FINAL_P11-FESIK 8TH FLOOR-92297.D
Method ULTRA2_AQUEOUS_HM.M
Location: 1,1:B,4
Description: Easy-Access Method: 'Ultra Aqueous High Mass' ,844.00
3: ELSD, Voltage
ELSD
Page 2 of 2
Printed: 6/24/2019 3:29 PM

Compound 20

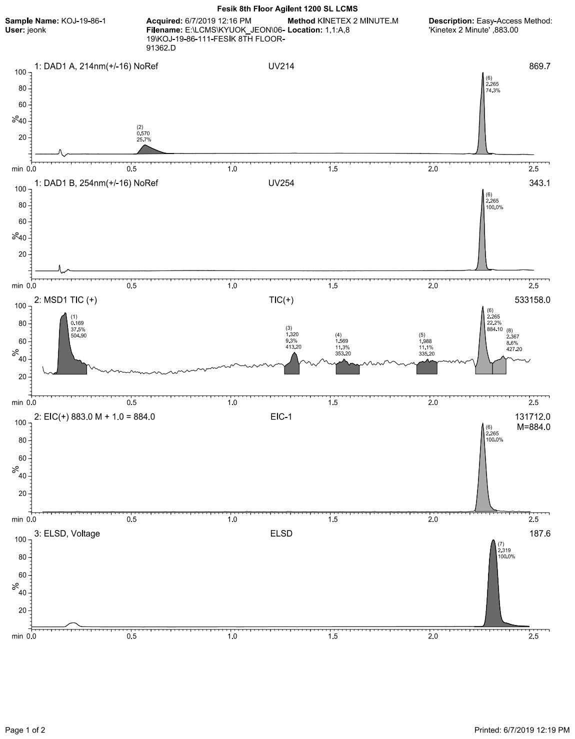
Fesik 8th Floor Agilent 1200 SL LCMS
Sample Name: KOJ-19-86-1
User: jeonk
Acquired: 6/7/2019 12:16 PM
Filename: E:\LCMS\KYUOK_JEON\06-19\KOJ-19-86-111-FESIK 8TH FLOOR-91362.D
Method KINETEX 2 MINUTE.M
Location: 1,1:A,8
Description: Easy-Access Method: 'Kinetex 2 Minute' ,883.00
1: DAD1 A, 214nm(+/-16) NoRef
UV214
869.7
1: DAD1 B, 254nm(+/-16) NoRef
UV254
343.1
2: MSD1 TIC (+)
TIC(+)
533158.0
2: EIC(+) 883.0 M + 1.0 = 884.0
EIC-1
131712.0
M=884.0
3: ELSD, Voltage
ELSD
187.6
Page 1 of 2
Printed: 6/7/2019 12:19 PM

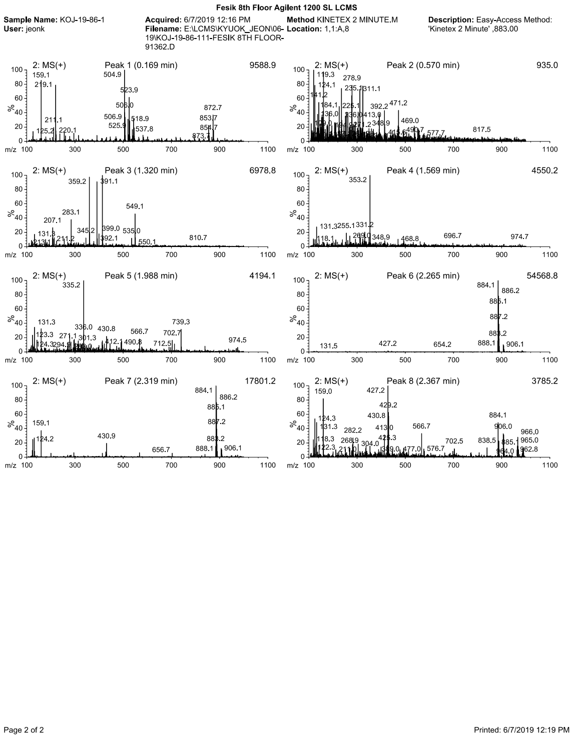
Fesik 8th Floor Agilent 1200 SL LCMS
Sample Name: KOJ-19-86-1
User: jeonk
Acquired: 6/7/2019 12:16 PM
Filename: E:\LCMS\KYUOK_JEON\06-19\KOJ-19-86-111-FESIK 8TH FLOOR-91362.D
Method KINETEX 2 MINUTE.M
Location: 1,1:A,8
Description: Easy-Access Method: 'Kinetex 2 Minute' ,883.00
2: MS(+) Peak 1 (0.169 min) 9588.9
2: MS(+) Peak 2 (0.570 min) 935.0
2: MS(+) Peak 3 (1.320 min) 6978.8
2: MS(+) Peak 4 (1.569 min) 4550.2
2: MS(+) Peak 5 (1.988 min) 4194.1
2: MS(+) Peak 6 (2.265 min) 54568.8
2: MS(+) Peak 7 (2.319 min) 17801.2
2: MS(+) Peak 8 (2.367 min) 3785.2
Page 2 of 2
Printed: 6/7/2019 12:19 PM

Compound 21

Fesik 8th Floor Agilent 1200 SL LCMS

Sample Name: jct-M-frac-12
User: tarrjc

Acquired: 11/15/2019 11:58 AM
Filename: C:\EZXDATA\CHRIS_TARR\11-19\JCT-M-FRAC-121-KINETEX 2 MINUTE-00643.D

Method KINETEX 2 MINUTE.M
Location: 1,1:A,1

Description: Easy-Access Method: 'Kinetex 2 Minute' ,796.00

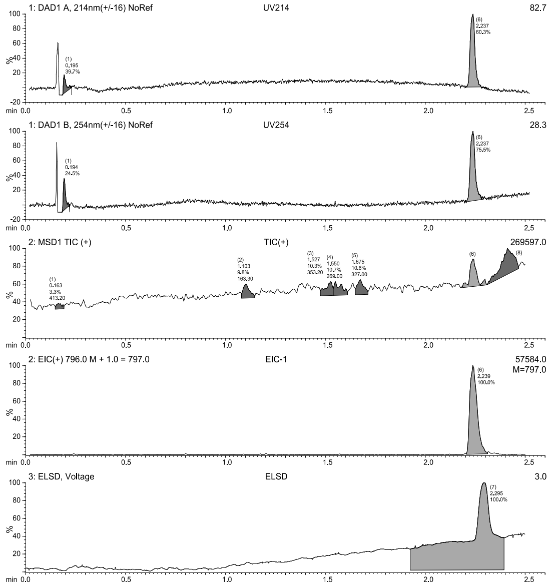

Page 1 of 2 Printed: 3/19/2025 10:19 AM

Fesik 8th Floor Agilent 1200 SL LCMS

Sample Name: jct-M-frac-12
User: tarrjc

Acquired: 11/15/2019 11:58 AM
Filename: C:\EZXDATA\CHRIS_TARR\11-19\JCT-M-FRAC-121-KINETEX 2 MINUTE-00643.D

Method KINETEX 2 MINUTE.M
Location: 1,1:A,1

Description: Easy-Access Method: 'Kinetex 2 Minute' ,796.00

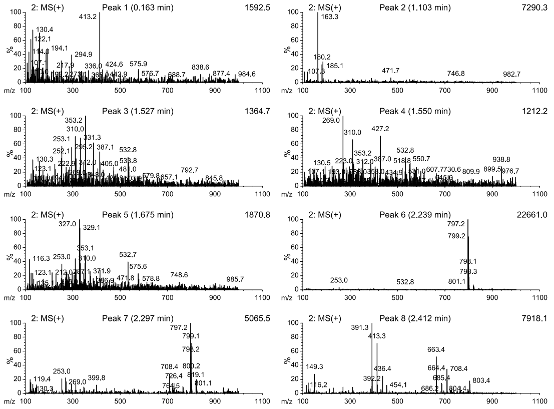

Page 2 of 2 Printed: 3/19/2025 10:19 AM

Compound 22

Fesik 8th Floor Agilent 1200 SL LCMS

Sample Name: jct-M-frac-32
User: tarrjc
Acquired: 11/18/2019 2:44 PM
Filename: C:\EZXDATA\CHRIS_TARR\11-19\JCT-M-FRAC-321-ULTRA2_AQUEOUS-00797.D
Method ULTRA2_AQUEOUS.M
Location: 1,1:A,8
Description: Easy-Access Method: 'Ultra Aqueous'

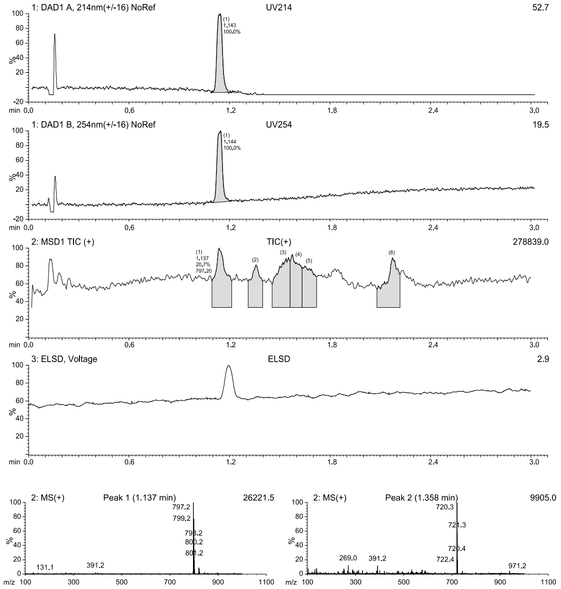

Printed: 3/19/2025 10:21 AM

Fesik 8th Floor Agilent 1200 SL LCMS

Sample Name: jct-M-frac-32
User: tarrjc
Acquired: 11/18/2019 2:44 PM
Filename: C:\EZXDATA\CHRIS_TARR\11-19\JCT-M-FRAC-321-ULTRA2_AQUEOUS-00797.D
Method ULTRA2_AQUEOUS.M
Location: 1,1:A,8
Description: Easy-Access Method: 'Ultra Aqueous'

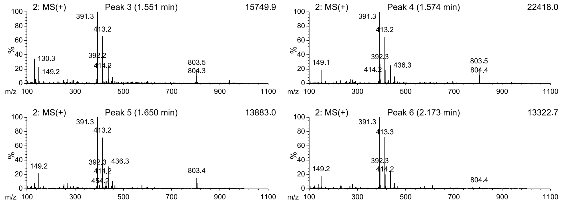

Printed: 3/19/2025 10:21 AM

Compound 23

Fesik 8th Floor Agilent 1200 SL LCMS

Sample Name: jct-o-914846
User: tarrjc
Acquired: 3/18/2025 3:07 PM
Filename: C:\EZXDATA\CHRIS_TARR\03-25\JCT-O-9148461-ULTRA2_AQUEOUS-77294.D
Method ULTRA2_AQUEOUS.M
Location: 1,1:F,5
Description: Easy-Access Method: 'Ultra Aqueous'

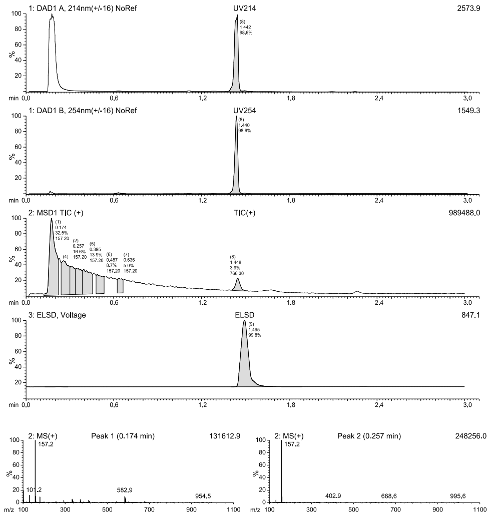

Printed: 3/18/2025 3:12 PM

Fesik 8th Floor Agilent 1200 SL LCMS

Sample Name: jct-o-914846
User: tarrjc
Acquired: 3/18/2025 3:07 PM
Filename: C:\EZXDATA\CHRIS_TARR\03-25\JCT-O-9148461-ULTRA2_AQUEOUS-77294.D
Method ULTRA2_AQUEOUS.M
Location: 1,1:F,5
Description: Easy-Access Method: 'Ultra Aqueous'

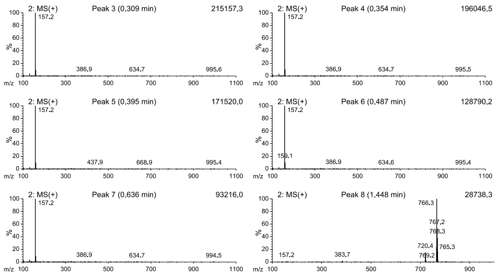

Printed: 3/18/2025 3:12 PM

Compound 24

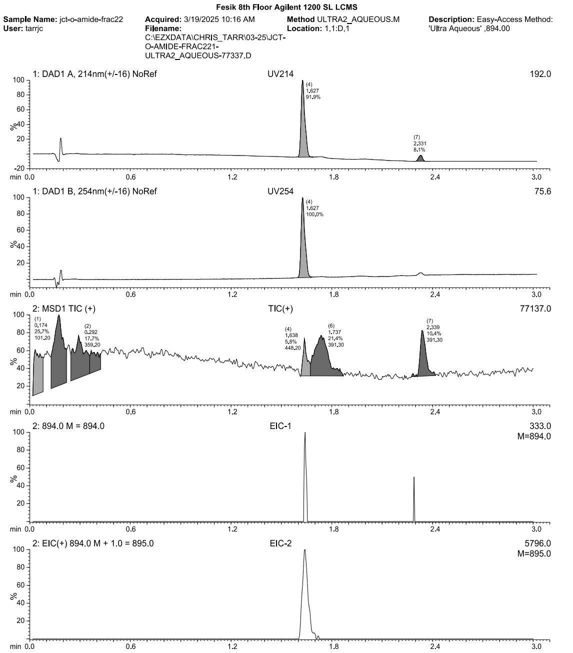

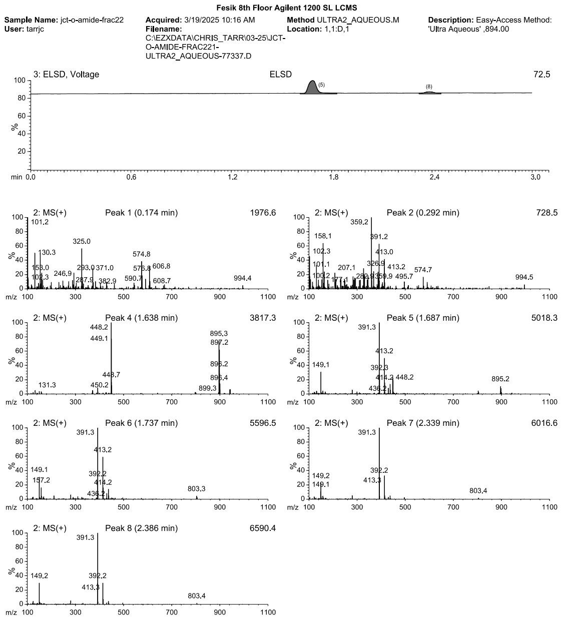

Page 1 of 2 Printed: 3/19/2025 10:20 AM

Page 2 of 2 Printed: 3/19/2025 10:20 AM

Compound 25

Fesik 8th Floor Agilent 1200 SL LCMS

Sample Name: jct-m-nme2-frac-a
User: tarrjc
Acquired: 9/27/2019 2:19 PM
Filename: E:\LCMS\CHRIS_TARR\09-19\JCT-M-NME2-FRAC-A11-FESIK 8TH FLOOR-97644.D
Method ULTRA2_AQUEOUS.M
Location: 1,1:C,9
Description: Easy-Access Method: 'Ultra II Aqueous'

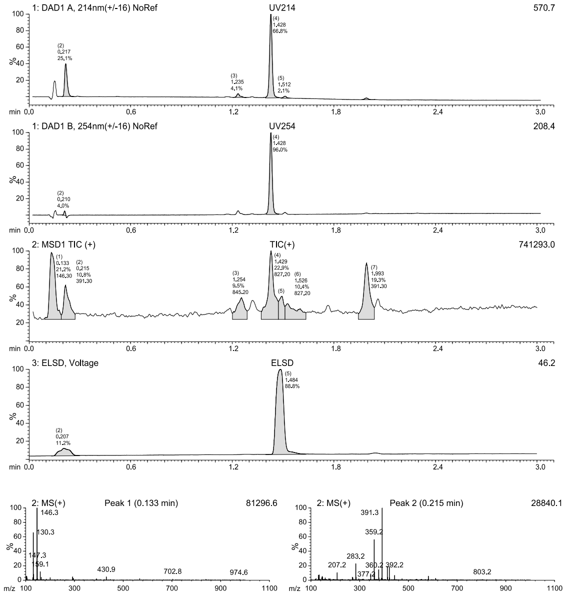

Printed: 3/10/2025 3:54 PM

Fesik 8th Floor Agilent 1200 SL LCMS

Sample Name: jct-m-nme2-frac-a
User: tarrjc
Acquired: 9/27/2019 2:19 PM
Filename: E:\LCMS\CHRIS_TARR\09-19\JCT-M-NME2-FRAC-A11-FESIK 8TH FLOOR-97644.D
Method ULTRA2_AQUEOUS.M
Location: 1,1:C,9
Description: Easy-Access Method: 'Ultra II Aqueous'

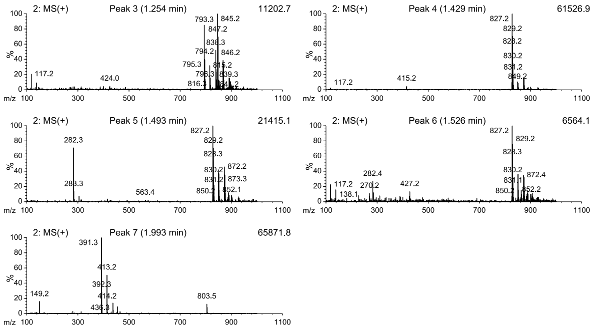

Printed: 3/10/2025 3:54 PM

S6. Synthesis of Intermediates, Compounds 21, 22, and 23

General Procedure A: Ullmann Cross Coupling. In a reaction vessel, the (*R*)-methyl-dihydropyrazinoindolone core (e.g., **30**, **38**, or **43**) (1.0 eq), 7-bromoindole or 7-iodoindole (1.5 eq), CuI (0.5 eq), (*trans*)-1,2-*N*,*N*'-dimethylaminocyclohexane (0.5 eq), and K_2CO_3 (2.0 eq) were weighed. The reaction vessel was charged with toluene (0.4 M) and sparged with argon for 5 min. The vessel was then sealed and heated to 100 °C for 48 h. The reaction was cooled to room temperature and diluted with 1:1 EtOAc/H_2O. The aqueous layer was separated and extracted with EtOAc (2x). The combined organic layers were washed with sat. NH_4Cl, sat. $NaHCO_3$, water, and brine. The organic layer was dried over $MgSO_4$, filtered, and concentrated. The crude residue was purified by flash column chromatography eluting with EtOAc/hexanes to afford the desired compound.

General Procedure B: Buchwald Cross Coupling. In a reaction vessel, the (*R*)-methyl-dihydropyrazinoindolone core (e.g., **30**, **38**, or **43**) (1.0 eq), 7-bromoindole or 7-iodoindole (1.2 eq), $[Pd(cinnamyl)Cl]_2$ (0.05 eq), ^{t}Bu-BrettPhos (0.1 eq), and Cs_2CO_3 (4.0 eq) were weighed. The reaction vessel was charged with toluene (0.2 M) and sparged with argon for 5 min. The reaction was sealed and heated to 100 °C for 2-24 h. The reaction was cooled to room temperature and diluted with 1:1 EtOAc/H_2O. The aqueous layer was separated and extracted with EtOAc (2x). The combined organic layers were washed with sat. NH_4Cl, sat. $NaHCO_3$, water, and brine. The organic layer was dried over $MgSO_4$, filtered, and concentrated. The crude residue was purified by flash column chromatography eluting with EtOAc/hexanes to afford the desired compound.

General Procedure C: Hydrogenolysis of Benzyl Ether. In a reaction vessel, the benzylic ether (e.g., **32, 38, 39,** or **47**) (1.0 eq) was dissolved in THF/^{i}PrOH (3:1) and the resultant mixture

was sparged with argon for 5 min. Pd/C (10% wt., 0.1 eq) and $Pd(OH)_2/C$ (20% wt., 0.1 eq) were added to the reaction vessel, and the reaction was flushed with argon. The reaction mixture was allowed to stir under an atmosphere of H_2 at 40 °C until complete by LCMS. The reaction mixture was filtered through a pad of Celite, rinsed with DCM, and concentrated. The crude residue was used without further purification.

General Procedure D: Tosylation of Alcohol. In a reaction vessel, the alcohol (e.g. **40**) (1.0 eq) was dissolved in DCM (0.08 M). TEA (10 eq) and DMAP (0.10 eq) were added and the reaction was stirred for 5 min at RT. The reaction was cooled to 0 °C and p-toluenesulfonic anhydride (4.0 eq) was added. The reaction was allowed to stir at 0 °C for 10 minutes and then warmed to RT for 2 h. The reaction was extracted with DCM, washed with brine, dried over $MgSO_4$, filtered, and concentrated. The crude residue was purified by flash column chromatography eluting with 0 to 100% EtOAc in hexanes to afford the desired product.

General Procedure E: Macrocyclization. In a reaction vessel, the tosylate (e.g. **41**, **46**) was dissolved in DMF (0.01 M) under an atmosphere of argon. Cesium carbonate (3.0 eq) was added, and the reaction was heated to 60 °C and stirred for 16 h. The reaction was extracted with EtOAc, washed with H_2O, washed with brine, dried over $MgSO_4$, filtered, and concentrated. The crude residue was purified by flash column chromatography eluting with 0 to 50% 95:5 EtOAc/MeOH in hexanes to afford the desired product.

General Procedure F: Saponification of Indole Ester. In a reaction vessel, the ester (e.g., **35**, **42**, **49**) was dissolved in THF/MeOH/H_2O (5:1:1, 0.2 M). LiOH (10 eq.) was added, and the reaction was heated at 50 °C for 3-24 h until the LCMS shows complete conversion. The reaction was extracted with DCM, acidified with 1M HCl, washed with H_2O, washed with brine, dried over

$MgSO_4$, filtered, and concentrated. The crude residue was purified by reverse phase HPLC eluting with MeCN/H_2O with 0.1% TFA additive. The resultant compound was concentrated, dissolved in DCM, washed with aq. $NaHCO_3$, dried with $MgSO_4$, filtered, and concentrated to afford the desired product.

Intermediate Synthesis

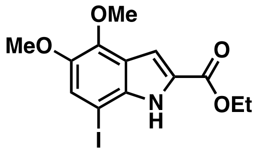

Ethyl 7-iodo-4,5-dimethoxy-1*H*-indole-2-carboxylate (31)

Compound **31A** (2.6 g, 7.92 mmol, 1.0 eq) was dissolved in 1,4-dioxane (7 mL). NaI (3.56 g, 23.8 mmol, 3.0 eq), CuI (75 mg, 0.4 mmol, 0.05 eq), and *trans*-(1*R*,2*R*)-*N*1,*N*2-dimethylcyclohexane-1,2-diamine (113 mg, 0.79 mmol, 0.1 eq) were added, and the reaction mixture was degassed with argon. The reaction was stirred at 110 °C for 60 h. The reaction was extracted with EtOAc, washed with 30% NH_4OH, washed with brine, dried over $MgSO_4$, filtered, and concentrated. The crude residue was purified by flash column chromatography eluting with 0 to 10% EtOAc in hexanes to afford the title compound (2.25 g, 76% yield). LCMS (ESI) Method 1: R_T = 1.783 min, m/z = 375.90 $[M+H]^+$.

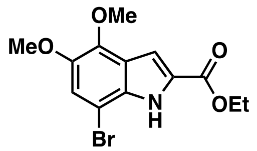

Ethyl 7-bromo-4,5-dimethoxy-1*H*-indole-2-carboxylate (31A)

Step A: Ethyl (*Z*)-2-azido-3-(5-bromo-2,3-dimethoxyphenyl)acrylate. In a round bottomed flask, NaOEt (36 mL, 21% wt., 111 mmol, 2.3 eq) was charged followed by EtOH (100 mL) and cooled to 0 °C. 5-Bromo-2,3-dimethoxybenzaldehyde (12 g, 48 mmol, 1.0 eq), ethyl azidoacetate (12.7 g, 99 mmol, 2.0 eq), and ethyl trifluoroacetate (14 g, 99 mmol, 2.0 eq) were dissolved in THF/EtOH (40 mL; 1:1) and added to the NaOEt solution at 0 °C. The reaction was allowed to stir for 2 h at 0 °C, and then warmed to RT for 1 h. The reaction was cooled to 0 °C and filtered and rinsed with cold EtOH to afford the desired product as a white solid. The crude reaction product was purified by flash column chromatography eluting with EtOAc in hexanes to afford the title compound (11 g, 63% yield). LCMS (ESI) Method 1: R_T = 2.051 min, product mass not observed.

Step B: Ethyl 7-bromo-4,5-dimethoxy-1*H*-indole-2-carboxylate. Ethyl (*Z*)-2-azido-3-(5-bromo-2,3-dimethoxyphenyl)acrylate (11 grams, 31 mmol) was dissolved in toluene (120 mL) and irradiated for 10 minutes in a laboratory microwave at 180 °C. The reaction was concentrated, and the crude product was purified by flash column chromatography eluting with 0 to 30% EtOAc in hexanes to afford the title compound (5.5 grams, 54% yield). LCMS (ESI) Method 1: R_T = 1.708 min, *m/z* = 327.9 $[M+H]^+$.

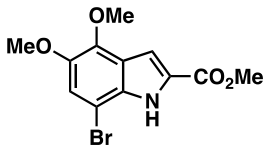

Methyl 7-bromo-4,5-dimethoxy-1*H*-indole-2-carboxylate (31B)

Step A: Methyl (*Z*)-2-azido-3-(5-bromo-2,3-dimethoxyphenyl)acrylate. In a reaction vessel 5-bromo-2,3-dimethoxybenzaldehyde (1.0 g, 4.1 mmol, 1.0 eq) was dissolved in MeOH (2 mL) and THF (2 mL) at -20 °C under argon. Methyl azidoacetate (1.6 mL, 16.3 mmol, 4.0 eq) in MeOH (2 mL) was added, followed by NaOMe (881 mg, 16.3 mmol. 4.0 eq) in MeOH (12 mL) added dropwise. The reaction was warmed to 0 °C and allowed to stir overnight. The reaction was quenched with aq. NH_4Cl, extracted with EtOAc, washed with H_2O, dried over $MgSO_4$, filtered, and concentrated. The crude residue was purified by flash column chromatography eluting with 0 to 15% EtOAc in hexanes to afford the title compound (903 mg, 65%). LCMS (ESI) Method 1: R_T = 2.045 min, *m/z* = 235.9 (fragment).

Step B: Methyl 7-bromo-4,5-dimethoxy-1*H*-indole-2-carboxylate. The title compound (356 mg, 74% yield) was prepared following General Procedure G using methyl (*Z*)-2-azido-3-(5-bromo-2,3-dimethoxyphenyl)acrylate (523 mg, 1.53 mmol). Upon completion, the crude residue was purified by flash column chromatography eluting with 0 to 10% EtOAc in hexanes. LCMS (ESI) Method 1: R_T = 1.704 min, *m/z* = 313.9 (M+H).

Synthesis of Compound 21

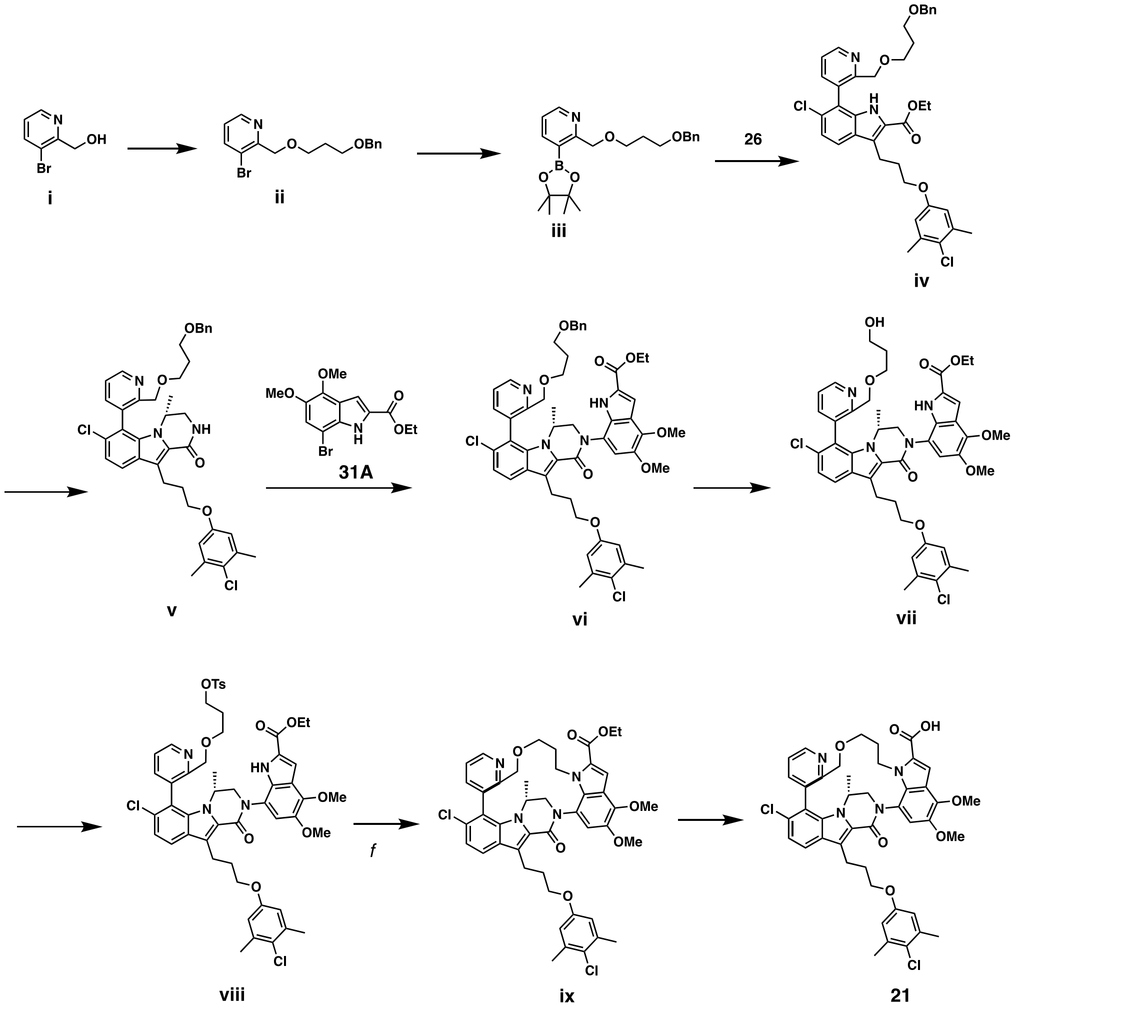

2-((3-(benzyloxy)propoxy)methyl)-3-bromopyridine (ii). (3-Bromopyridin-2-yl)methanol (**i**) (2.0 g, 10.6 mmol, 1.0 eq) was dissolved in DMF/THF (1:1, 20 mL). NaH (425 mg, 16.0 mmol, 1.5 eq) was added at room temperature and the reaction was stirred for 20 min. ((3-Bromopropoxy)methyl)benzene (4.39 g, 19.1 mmol, 1.8 eq) was then added, and the reaction was allowed to stir for 16 h at RT. The reaction was incomplete as determined by LCMS chromatography. Sodium hydride (141 mg, 5.33 mmol, 0.5 eq) was added again at RT, and the reaction allowed to stir an additional 4 h. The reaction was quenched with MeOH (5 mL), extracted with DCM, washed with brine, dried over $MgSO_4$, filtered, and then concentrated. The

crude product was purified by flash column chromatography eluting with 0 to 50% EtOAc in hexanes to afford the title compound (2.49 g, 70% yield). LCMS (ESI) Method 1: R_T = 1.511 min, *m/z* = 336.10 $[M+H]^+$.

2-((3-(benzyloxy)propoxy)methyl)-3-(4,4,5,5-tetramethyl-1,3,2-dioxaborolan-2-yl)pyridine (iii). In a round-bottomed flask, 2-((3-(benzyloxy)propoxy)methyl)-3-bromopyridine (**ii**) (1.55 g, 4.61 mmol, 1.0 eq) was dissolved in THF (20 mL) and cooled to -78 °C. 2-Isopropoxy-4,4,5,5-tetramethyl-1,3,2-dioxaborolane (4.29 g, 23.0 mmol, 5.0 eq) was added, followed by addition of *n*-BuLi (4.79 mL, 12.0 mmol, 2.6 eq) over 20 min. The reaction was stirred for 1 h at -78 °C, then quenched with MeOH. The reaction was extracted with EtOAc, washed with brine, dried over $MgSO_4$, filtered, and concentrated. The crude product was purified by flash column chromatography eluting with 0 to 50% EtOAc in hexanes, followed by 20% MeOH in DCM to afford the title compound (894 mg, 51% yield).

Ethyl 7-(2-((3-(benzyloxy)propoxy)methyl)pyridin-3-yl)-6-chloro-3-(3-(4-chloro-3,5-dimethylphenoxy)propyl)-1*H*-indole-2-carboxylate (iv). In a heavy walled vial, compound **26** (1.60 g, 3.21 mmol, 1.0 eq), compound **iii** (1.42 g, 3.71 mmol, 1.15 eq), K_2CO_3 (1.33 g, 9.6 mmol, 3.0 eq), and $Pd(PPh_3)_4$ (0.37 g, 0.32 mmol, 0.1 eq) were added and dissolved in dioxane (25 mL) and H_2O (5 mL). The solution was sparged with argon for 5 min, then the reaction was sealed and heated to 100 °C for 8 h. The reaction was cooled to RT, extracted with EtOAc, dried over MgSO4, filtered, and concentrated. The crude material was purified by flash column chromatography, eluting with 0 to 100% EtOAc in hexanes to afford the title compound (2.09 g, 95% yield). LCMS (ESI) Method 1: R_T = 2.296 min, *m/z* = 675.20 $[M+H]^+$.

(4*R*)-6-(2-((3-(Benzyloxy)propoxy)methyl)pyridin-3-yl)-7-chloro-10-(3-(4-chloro-3,5-dimethylphenoxy)propyl)-4-methyl-3,4-dihydropyrazino[1,2-*a*]indol-1(2*H*)-one (v). Compound **iv** (2.09 g, 3.09 mmol, 1 eq) was dissolved in MeCN (30 mL). *tert*-Butyl (*S*)-5-methyl-1,2,3-oxathiazolidine-3-carboxylate 2,2-dioxide (2.20 g, 9.28 mmol, 3.0 eq) and cesium carbonate (5.04 g, 15.5 mmol, 5.0 eq) were added, and the reaction was heated to 70 °C for 16 h. The reaction was extracted with DCM, dried over $MgSO_4$, filtered, and then concentrated. The crude reaction was then purified by flash column chromatography eluting with 0 to 100% EtOAc in hexanes, then 0 to 10% MeOH in DCM. The product was isolated and taken up in DCM (20 mL). Trifluoroacetic acid (2 mL) was added, and the reaction was stirred at RT for 3 h. The reaction was concentrated, and the crude residue was taken up in MeOH (20 mL). Potassium carbonate (2.14 g, 15.5 mmol, 5.0 eq) was added, and the reaction was heated to 50 °C for 2 h. The solvent was removed and the crude residue taken up in DCM, washed with brine, dried over $MgSO_4$, filtered, and concentrated. The crude product was purified by flash column chromatography eluting with 0 to 20% MeOH in DCM to afford the title compound (545 mg, 26% yield) as the second eluting peak with the same mass. LCMS (ESI) Method 1: R_T = 2.164 min, *m/z* = 686.20 $[M+H]^+$.

Ethyl 7-((4*R*)-6-(2-((3-(benzyloxy)propoxy)methyl)pyridin-3-yl)-7-chloro-10-(3-(4-chloro-3,5-dimethylphenoxy)propyl)-4-methyl-1-oxo-3,4-dihydropyrazino[1,2-*a*]indol-2(1*H*)-yl)-4,5-dimethoxy-1*H*-indole-2-carboxylate (vi). The title compound (306 mg, 91% yield) was prepared following General Procedure B using compound **v** (250 mg, 0.36 mmol, 1.0 eq) and compound **31A** (143 mg, 0.45 mmol, 1.2 eq). LCMS (ESI) Method 2: R_T = 1.733 min, *m/z* = 933.3 $[M+H]^+$.

Ethyl 7-((4*R*)-7-chloro-10-(3-(4-chloro-3,5-dimethylphenoxy)propyl)-6-(2-((3-hydroxypropoxy)methyl)pyridin-3-yl)-4-methyl-1-oxo-3,4-dihydropyrazino[1,2-*a*]indol-

2(1*H*)-yl)-4,5-dimethoxy-1*H*-indole-2-carboxylate (vii). The title compound was prepared following General Procedure C using compound **vi** (306 mg, 0.33 mmol, 1.0 eq). Upon workup, the reaction was carried forward without further purification. LCMS (ESI) Method 4: R_T = 0.882 min, 0.934 min, *m/z* = 843.3 $[M+H]^+$.

Ethyl 7-((4*R*)-7-chloro-10-(3-(4-chloro-3,5-dimethylphenoxy)propyl)-4-methyl-1-oxo-6-(2-((3-(tosyloxy)propoxy)methyl)pyridin-3-yl)-3,4-dihydropyrazino[1,2-*a*]indol-2(1*H*)-yl)-4,5-dimethoxy-1*H*-indole-2-carboxylate (viii). The title compound (221 mg, 67% yield over Steps F and G) was prepared following General Procedure D using compound **vii** (0.33 mmol, 1.0 eq). The crude product was purified by flash column chromatography eluting with 0 to 100% EtOAc in hexanes then 0 to 10% MeOH in DCM. LCMS (ESI) Method 2: R_T = 1.618 min, *m/z* = 997.2 $[M+H]^+$.

Ethyl ($2^63^4S_a$,$1^72^2R_a$,2^4R)-2^7-chloro-2^{10}-(3-(4-chloro-3,5-dimethylphenoxy) propyl)-1^4,1^5-dimethoxy-2^4-methyl-2^1-oxo-2^1,2^2,2^3,2^4-tetrahydro-1^1H-5-oxa-2(2,6)-pyrazino[1,2-*a*]indola-1(7,1)-indola-3(3,2)-pyridinacyclooctaphane-1^2-carboxylate (ix). The title compound (113 mg, 62% yield) was prepared following General Procedure E using compound **viii** (221 mg, 0.23 mmol, 1.0 eq). The crude product was purified by flash column chromatography to afford the title compound (113 mg, 62% yield). LCMS (ESI) Method 2: R_T = 1.496 min, *m/z* = 825.3 $[M+H]^+$.

($2^63^4S_a$,$1^72^2R_a$,2^4R)-2^7-Chloro-2^{10}-(3-(4-chloro-3,5-dimethylphenoxy)propyl)-1^4,1^5-dimethoxy-2^4-methyl-2^1-oxo-2^1,2^2,2^3,2^4-tetrahydro-1^1H-5-oxa-2(2,6)-pyrazino[1,2-*a*]indola-1(7,1)-indola-3(3,2)-pyridinacyclooctaphane-1^2-carboxylic acid (21) The title compound (47 mg, 42% yield) was prepared following General Procedure F using compound **ix** (113 mg, 0.14 mmol, 1.0 eq). The crude product was purified by reverse phase HPLC eluting with

MeCN/H_2O/TFA. The fractions containing product were diluted into DCM, neutralized with saturated $NaHCO_3$ solution, and concentrated to afford the title compound. LCMS (ESI) Method 1: R_T = 2.237 min, m/z = 797.2 $[M+H]^+$.

Synthesis of Compound 22

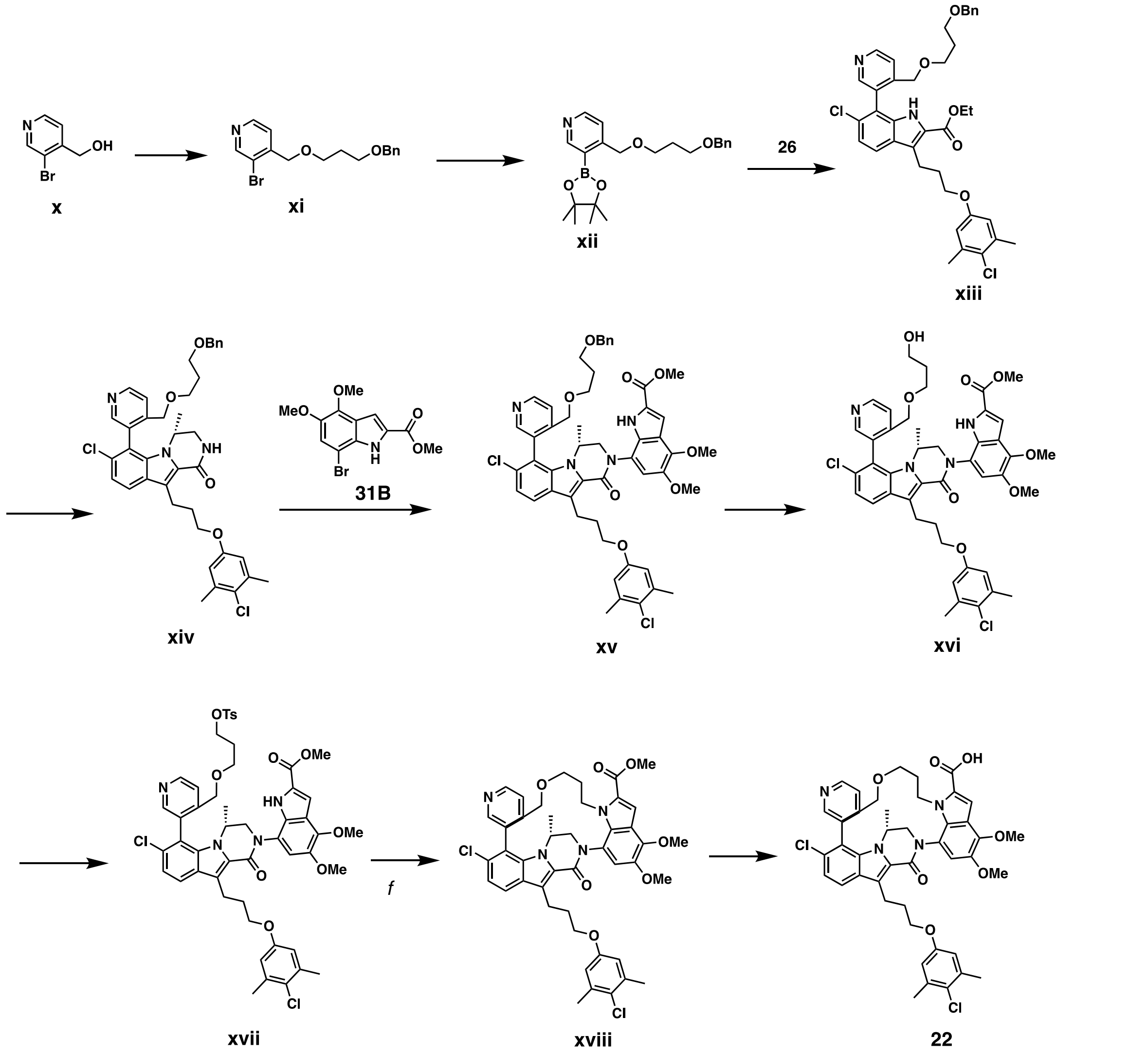

4-((3-(benzyloxy)propoxy)methyl)-3-bromopyridine (xi). (3-Bromopyridin-4-yl)methanol (**i**) (500 mg, 2.65 mmol, 1.0 eq) was dissolved in DMF/THF (1:1, 6 mL). NaH (212 mg, 8.0 mmol, 3.0 eq) was added at room temperature and the reaction was stirred for 20 min. ((3-

Bromopropoxy)methyl)benzene (1.10 g, 4.8 mmol, 1.8 eq) was then added, and the reaction was allowed to stir for 6 h at RT. The reaction was quenched with MeOH (5 mL), extracted with DCM, washed with brine, dried over $MgSO_4$, filtered, and then concentrated. The crude product was purified by reverse phase HPLC to afford the title compound (375 mg, 42% yield). LCMS (ESI) Method 1: R_T = 1.802 min, *m/z* = 336.10 $[M+H]^+$.

4-((3-(benzyloxy)propoxy)methyl)-3-(4,4,5,5-tetramethyl-1,3,2-dioxaborolan-2-yl)pyridine (xii). In a round-bottomed flask, 2-((3-(benzyloxy)propoxy)methyl)-3-bromopyridine (**xi**) (375 mg, 1.12 mmol, 1.0 eq) was dissolved in THF (20 mL) and cooled to -78 °C. 2-Isopropoxy-4,4,5,5-tetramethyl-1,3,2-dioxaborolane (1.0 g, 5.4 mmol, 5.0 eq) was added, followed by addition of *n*-BuLi (1.3 mL, 2.8 mmol, 2.6 eq) over 20 min. The reaction was stirred for 1 h at -78 °C, then quenched with MeOH. The reaction was extracted with EtOAc, washed with brine, dried over $MgSO_4$, filtered, and concentrated. The crude product was purified by flash column chromatography eluting with 0 to 50% EtOAc in hexanes, followed by 20% MeOH in DCM to afford the title compound (190 mg, 44% yield).

Ethyl 7-(4-((3-(benzyloxy)propoxy)methyl)pyridin-3-yl)-6-chloro-3-(3-(4-chloro-3,5-dimethylphenoxy)propyl)-1*H*-indole-2-carboxylate (xiii). In a heavy walled vial, compound **26** (0.55 g, 1.11 mmol, 1.0 eq), compound **xii** (0.67 g, 2.22 mmol, 2 eq), K_2CO_3 (0.46 g, 3.3 mmol, 3.0 eq), and $Pd(PPh_3)_4$ (0.13 g, 0.11 mmol, 0.1 eq) were added and dissolved in dioxane (15 mL) and H_2O (3 mL). The solution was sparged with argon for 5 min, then the reaction was sealed and heated to 100 °C for 8 h. The reaction was cooled to room temperature, extracted with EtOAc, dried over $MgSO_4$, filtered, and concentrated. The crude material was purified by flash column chromatography, eluting with 0 to 100% EtOAc in hexanes to afford the title compound (56 g, 75% yield). LCMS (ESI) Method 2: R_T = 1.02 min, *m/z* = 675.60 $[M+H]^+$.

(4*R*)-6-(4-((3-(Benzyloxy)propoxy)methyl)pyridin-3-yl)-7-chloro-10-(3-(4-chloro-3,5-dimethylphenoxy)propyl)-4-methyl-3,4-dihydropyrazino[1,2-*a*]indol-1(2*H*)-one (xiv). Compound **xiii** (0.56 g, 0.83 mmol, 1 eq) was dissolved in MeCN (15 mL). *tert*-Butyl (*S*)-5-methyl-1,2,3-oxathiazolidine-3-carboxylate 2,2-dioxide (0.4 g, 1.66 mmol, 2 eq.) and cesium carbonate (0.8 g, 2.47 mmol, 3.0 eq) were added, and the reaction was heated to 70 °C for 16 h. The reaction was concentrated, diluted with EtOAc, dried over $MgSO_4$, filtered, and then concentrated. The product was dissolved in DCM (10 mL). Trifluoroacetic acid (2 mL) was added, and the reaction was stirred at RT for 3 h. The reaction was concentrated, and the crude residue was dissolved in ethanol (20 mL). K_2CO_3 (0.58 g, 4.15 mmol, 5.0 eq) was added, and the reaction was heated to 60 °C for 2 h. The solvent was removed and the crude residue was dissolved in EtOAc and H_2O, extracted with ethyl acetate, washed with brine, dried over $MgSO_4$, filtered, and concentrated. The crude product was purified by flash column chromatography eluting with 0 to 5% MeOH in DCM to afford the title compound (144 mg, 26% over yield of the 3 reactions). LCMS (ESI) Method 2: R_T = 1.28 min, *m/z* = 832.6 $[M+H]^+$.

Methyl 7-((4*R*)-6-(4-((3-(benzyloxy)propoxy)methyl)pyridin-3-yl)-7-chloro-10-(3-(4-chloro-3,5-dimethylphenoxy)propyl)-4-methyl-1-oxo-3,4-dihydropyrazino[1,2-*a*]indol-2(1*H*)-yl)-4,5-dimethoxy-1*H*-indole-2-carboxylate (xv). The title compound (155 mg, 80% yield) was prepared following General Procedure B using compound **xiv** (144 mg, 0.21 mmol, 1.0 eq) and methyl 7-bromo-4,5-dimethoxy-1H-indole-2-carboxylate (**31B**) (80 mg, 0.25 mmol, 1.2 eq). The crude product was purified by flash column chromatography eluting with 0 to 100% EtOAc to afford the title compound. LCMS (ESI) Method 4: R_T = 1.113 min, *m/z* = 919.6 $[M+H]^+$.

Methyl 7-((4*R*)-7-chloro-10-(3-(4-chloro-3,5-dimethylphenoxy)propyl)-6-(4-((3-hydroxypropoxy)methyl)pyridin-3-yl)-4-methyl-1-oxo-3,4-dihydropyrazino[1,2-*a*]indol-

2(1*H*)-yl)-4,5-dimethoxy-1*H*-indole-2-carboxylate (xvi). The title compound (quantitative yield) was prepared following General Procedure C using compound **xv** (155 mg, 0.17 mmol, 1.0 eq). LCMS (ESI) Method 4: R_T = 0.843 min, *m/z* = 829.6 $[M+H]^+$.

Methyl 7-((4*R*)-7-chloro-10-(3-(4-chloro-3,5-dimethylphenoxy)propyl)-4-methyl-1-oxo-6-(4-((3-(tosyloxy)propoxy)methyl)pyridin-3-yl)-3,4-dihydropyrazino[1,2-*a*]indol-2(1*H*)-yl)-4,5-dimethoxy-1*H*-indole-2-carboxylate (xvii). The title compound (123 mg, 74% yield over Steps E and F) was prepared following General Procedure D using compound **xvi** (0.17 mmol, 1.0 eq). LCMS (ESI) Method 2: R_T = 1.492 min, *m/z* = 983.1 $[M+H]^+$.

Methyl ($2^63^4S_a,1^72^2R_a,2^4R$)-2^7-chloro-2^{10}-(3-(4-chloro-3,5-dimethylphenoxy) propyl)-$1^4,1^5$-dimethoxy-2^4-methyl-2^1-oxo-$2^1,2^2,2^3,2^4$-tetrahydro-1^1H-5-oxa-2(2,6)-pyrazino[1,2-*a*]indola-1(7,1)-indola-3(3,4)-pyridinacyclooctaphane-1^2-carboxylate (xviii). The title compound (19 mg, 59% yield) was prepared following General Procedure E using compound **xvii** (39 mg, 0.040 mmol, 1.0 eq). Following aqueous workup, the crude product was purified by flash column chromatography eluting with 0 to 100% EtOAc in hexanes to afford the desired product. LCMS (ESI) Method 2: R_T = 1.254 min, *m/z* = 811.1 $[M+H]^+$.

($2^63^4S_a,1^72^2R_a,2^4R$)-2^7-Chloro-2^{10}-(3-(4-chloro-3,5-dimethylphenoxy)propyl)-$1^4,1^5$-dimethoxy-2^4-methyl-2^1-oxo-$2^1,2^2,2^3,2^4$-tetrahydro-1^1H-5-oxa-2(2,6)-pyrazino[1,2-*a*]indola-1(7,1)-indola-3(3,4)-pyridinacyclooctaphane-1^2-carboxylic acid (22). The title compound (9 mg, 47% yield) was prepared following General Procedure F using compound **xviii** (19 mg, 0.023 mmol, 1.0 eq). Following aqueous workup, the crude product was purified by reverse phase HPLC eluting with MeCN/H_2O/TFA. The fractions containing product were diluted into DCM,

neutralized with saturated $NaHCO_3$ solution, and concentrated to afford the title compound. LCMS (ESI) Method 1: R_T = 2.092 min, *m/z* = 797.2 $[M+H]^+$.

Synthesis of Compound 23

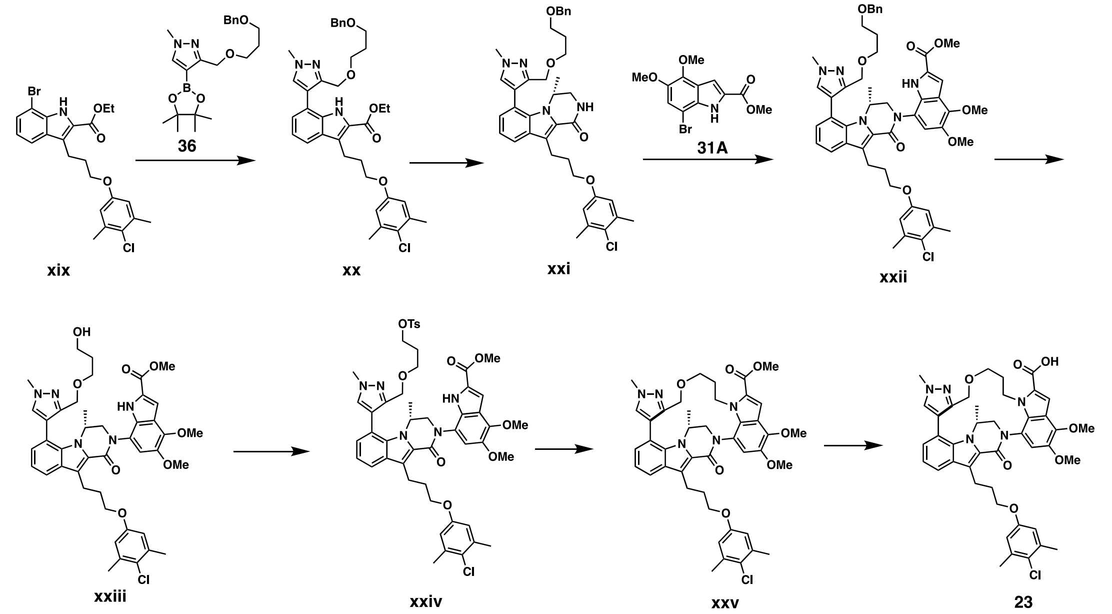

Ethyl 7-(3-((3-(benzyloxy)propoxy)methyl)-1-methyl-1*H*-pyrazol-4-yl)-3-(3-(4-chloro-3,5-dimethylphenoxy)propyl)-1*H*-indole-2-carboxylate (xx). In a heavy walled vial, ethyl 7-bromo-3-(3-(4-chloro-3,5-dimethylphenoxy)propyl)-1*H*-indole-2-carboxylate (**xix**) (12 g, 25.8 mmol, 1.0 eq) compound **36** (15 g, 38.7 mmol, 1.5 eq), K_2CO_3 (10.7 g, 77.5 mmol, 3.0 eq), and $Pd(PPh_3)_4$ (2.98 g, 2.58 mmol, 0.1 eq) were dissolved in dioxane (150 mL) and water (30 mL). The reaction was sparged with argon for 5 minutes, then heated to 90 °C for 10 h. The reaction was cooled to RT, extracted with EtOAc, dried over $MgSO_4$, filtered, and concentrated. The crude material was purified by flash column chromatography, eluting with 0 to 100% EtOAc in hexanes to afford the title compound (14.12 g, 85% yield). LCMS (ESI) Method 2: R_T = 1.36 min, *m/z* = 646.6 (M+H).

(*R*)-6-(3-((3-(Benzyloxy)propoxy)methyl)-1-methyl-1*H*-pyrazol-4-yl)-10-(3-(4-chloro-3,5-dimethylphenoxy)propyl)-4-methyl-3,4-dihydropyrazino[1,2-*a*]indol-1(2*H*)-one (xxi). Compound **xx** (14.12 g, 21.9 mmol, 1.0 eq) was dissolved in MeCN (130 mL). *tert*-Butyl (*S*)-5-methyl-1,2,3-oxathiazolidine-3-carboxylate 2,2-dioxide (9.2 g, 38.7 mmol, 1.5 eq.) and Cs_2CO_3 (18.5 g, 2.2 mmol, 2.2 eq) were added, and the reaction was heated to 70 °C for 16 h. The reaction was cooled to RT and concentrated. The residue was dissolved in EtOAc, washed with H_2O, dried over $MgSO_4$, filtered, and then concentrated. The crude residue was flashed by flash column chromatography eluting with 0 to 100% EtOAc in hexanes (17.5 g, quant. yield). LCMS (ESI) Method 2: R_T = 1.33 min, *m/z* = 801.7 $[M+H]^+$. The resultant material was dissolved in DCM (150 mL) and TFA (20 mL). The reaction was allowed to stir for 2 h, and the reaction was concentrated. The crude residue was taken up in EtOH (150 mL) and K_2CO_3 (18 g, 130 mmol, 5.9 eq) and the reaction was heated at 50 °C for 3 h. The reaction mixture was concentrated and the residue taken up in EtOAc/H_2O. The mixture was extracted with EtOAc, washed with H_2O, dried over $MgSO_4$, filtered, and concentrated. The crude residue was purified by flash column chromatography eluting with 0 to 100% DCM in EtOAc to afford the title compound (7.64 g, 53% yield). LCMS (ESI) Method 2: R_T = 0.99 min, *m/z* = 655.7 $[M+H]^+$.

Methyl (*R*)-7-(6-(3-((3-(benzyloxy)propoxy)methyl)-1-methyl-1*H*-pyrazol-4-yl)-10-(3-(4-chloro-3,5-dimethylphenoxy)propyl)-4-methyl-1-oxo-3,4-dihydropyrazino[1,2-*a*]indol-2(1*H*)-yl)-4,5-dimethoxy-1*H*-indole-2-carboxylate (xxii). The title compound (1.34 g, 98% yield) was prepared following General Procedure B using compound **xxi** (1.0 g, 1.53 mmol, 1.0 eq) and compound **31A** (600 mg, 1.91 mmol, 1.25 eq). Following workup, the crude residue was purified by flash column chromatography eluting with 0 to 100% EtOAc in hexanes. LCMS (ESI) Method 2: R_T = 1.852 min, *m/z* = 888.0 (M+H).

Methyl (*R*)-7-(10-(3-(4-chloro-3,5-dimethylphenoxy)propyl)-6-(3-((3-hydroxy-propoxy)methyl)-1-methyl-1*H*-pyrazol-4-yl)-4-methyl-1-oxo-3,4-dihydropyrazino[1,2-*a*] indol-2(1*H*)-yl)-4,5-dimethoxy-1*H*-indole-2-carboxylate (xxiii). The title compound (900 mg, 75% yield) was prepared following General Procedure C using compound **xxii** (1.34 g, 1.51 mmol, 1.0 eq). Following workup, the crude product was purified by flash column chromatography eluting with 0 to 100% EtOAc:MeOH (95:5) in hexanes. LCMS (ESI) Method 2: R_T = 1.310 min, *m/z* = 798.0 (M+H).

Methyl (*R*)-7-(10-(3-(4-chloro-3,5-dimethylphenoxy)propyl)-4-methyl-6-(1-methyl-3-((3-(tosyloxy)propoxy)methyl)-1*H*-pyrazol-4-yl)-1-oxo-3,4-dihydropyrazino[1,2-a]indol-2(1*H*)-yl)-4,5-dimethoxy-1H-indole-2-carboxylate (xxiv). The title compound (920 mg, 86% yield) was prepared following General Procedure D using compound **xxiii** (900 mg, 1.13 mmol, 1.0 eq). Following workup, the crude residue was purified by flash column chromatography eluting with 0 to 100% EtOAc in hexanes. LCMS (ESI) Method 2: R_T = 1.643 min, *m/z* = 952.0 (M+H).

Methyl ($2^63^4R_a,1^72^2R_a,2^4R$)-2^{10}-(3-(4-chloro-3,5-dimethylphenoxy)propyl)-$1^4,1^5$-dimethoxy-$2^4,3^1$-dimethyl-2^1-oxo-$2^1,2^2,2^3,2^4$-tetrahydro-$1^1H,3^1H$-5-oxa-2(2,6)-pyrazino[1,2-*a*]indola-1(7,1)-indola-3(4,3)-pyrazolacyclooctaphane-1^2-carboxylate (xxv). The title compound (690 mg, 80% yield) was prepared following General Procedure E using compound **xxiv** (920 mg, 1.11 mmol, 1.0 eq). Following workup, the crude residue was purified by flash column chromatography eluting with 0 to 100% EtOAc in hexanes. LCMS (ESI) Method 2: R_T = 1.653 min, *m/z* = 780.0 (M+H).

($2^63^4R_a,1^72^2R_a,2^4R$)-2^{10}-(3-(4-Chloro-3,5-dimethylphenoxy)propyl)-$1^4,1^5$-dimethoxy-$2^4,3^1$-dimethyl-2^1-oxo-$2^1,2^2,2^3,2^4$-tetrahydro-$1^1H,3^1H$-5-oxa-2(2,6)-pyrazino[1,2-*a*]indola-1(7,1)-

indola-3(4,3)-pyrazolacyclooctaphane-1^2-carboxylic acid (23). The title compound (660 mg, 97% yield) was prepared following General Procedure F using compound **xxv** (690 mg, 0.88 mmol). LCMS (ESI) Method 2: R_T = 1.281 min, *m/z* = 766.0 (M+H). ^{1}H NMR (DMSO-$_{d6}$, 400 MHz) δ 7.96 (s, 1H), 7.73 (d, *J* = 8.0 Hz, 1H), 7.29 (s, 1H), 7.17 (t, *J* = 7.2 Hz, 1H), 7.14 (s, 1H), 7.07 (d, *J* = 7.2 Hz, 1H), 6.67 (s, 2H), 4.58-4.43 (m, 3H), 4.40-4.33 (m, 1H), 4.26-4.16 (m, 2H), 4.08 (d, *J* = 9.2 Hz, 1H), 3.95 (s, 3H), 3.94 (s, 3H), 3.93-3.89 (m, 1H), 3.86 (s, 3H), 3.64 (d, *J* = 10.0 Hz, 2H), 3.30-3.22 (m, 2H), 3.02 (t, *J* = 10.0 Hz, 1H), 2.22 (s, 6H), 2.07-1.94 (m, 3H), 1.66-1.55 (m, 1H), 1.02 (d, *J* = 6.4 Hz, 3H).
